# Selective Iron Catalyzed Synthesis of N‐Alkylated Indolines and Indoles

**DOI:** 10.1002/chem.202201809

**Published:** 2022-07-22

**Authors:** Jiajun Wu, Satawat Tongdee, Marie Cordier, Christophe Darcel

**Affiliations:** ^1^ Univ Rennes CNRS ISCR (Institut des Sciences Chimiques de Rennes) UMR 6226 35000 Rennes France

**Keywords:** hydrogen auto-transfer, indole, indoline, iron, N-alkylation, oxidation

## Abstract

Whereas iron catalysts usually promote catalyzed C3‐alkylation of indole derivatives via a borrowing‐hydrogen methodology using alcohols as the electrophilic partners, this contribution shows how to switch the selectivity towards N‐alkylation. Thus, starting from indoline derivatives, N‐alkylation was efficiently performed using a tricarbonyl(cyclopentadienone) iron complex as the catalyst in trifluoroethanol in the presence of alcohols leading to the corresponding N‐alkylated indoline derivatives in 31–99 % yields (28 examples). The one‐pot, two‐step strategy for the selective N‐alkylation of indolines is completed by an oxidation to give the corresponding N‐alkylated indoles in 31–90 % yields (15 examples). This unprecedented oxidation methodology involves an iron salt catalyst associated with (2,2,6,6‐tetramethylpiperidin‐1‐yl)oxyl (TEMPO) and a stoichiometric amount of *t‐*BuOOH at room temperature.

## Graphical abstract

**In contrast to classical iron‐catalyzed** C3‐alkylations of indoles via the borrowing‐hydrogen methodology, this contribution describes the switch of the selectivity towards N‐alkylation. Starting from indolines, N‐alkylation was efficiently done using a tricarbonyl(cyclopentadienone) iron complex as the catalyst in 2,2,2‐trifluoroethanol. Notably, performing the one pot N‐alkylation of indolines, followed by oxidation using a FeBr_3_/TEMPO catalyst with *t‐*BuOOH, the corresponding N‐alkylated indoles were obtained.

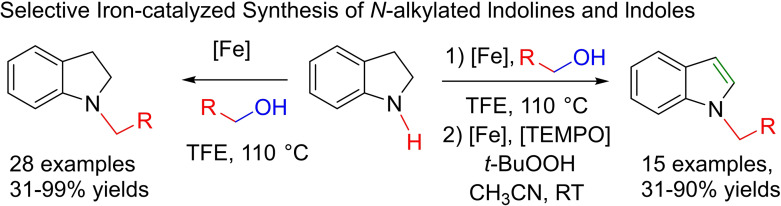

Among N‐heterocycles, indole and indoline scaffolds are core moieties due to their occurrence in numerous natural products, and bioactive compounds.^[1]^ Indeed, there are eight indole containing commercial drugs in the Top‐200 Small Molecules Pharmaceuticals Best Selling Drugs by Retail Sales in 2018.^[2]^ As representative examples, indole and indoline derivatives exhibit a broad spectrum of biological activities as highlighted in indole series by Arbidol **1**, a drug with a broad antiviral spectrum against influenza A and B,^[3]^ Ondansetron **2** used to prevent nausea and vomiting caused by cancer chemotherapy, radiation therapy or surgery,^[4]^ or Fluvastatin **3**, prescribed against hypercholes‐terolemia and to prevent cardiovascular diseases.^[5]^

Indoline motif is also an interesting substructure as demonstrated with Prajmaline **4**, an antiarrhythmic agent to treat cardiac disorders,^[6]^ Eserine (Physostigmine) **5**, used to treat glaucoma and delayed gastric emptying,^[7]^ or the compound **6**, a potent Cholesterol Ester Transfer Protein (CETP) Inhibitor.^[8]^ (Figure 1)

**Figure 1:**
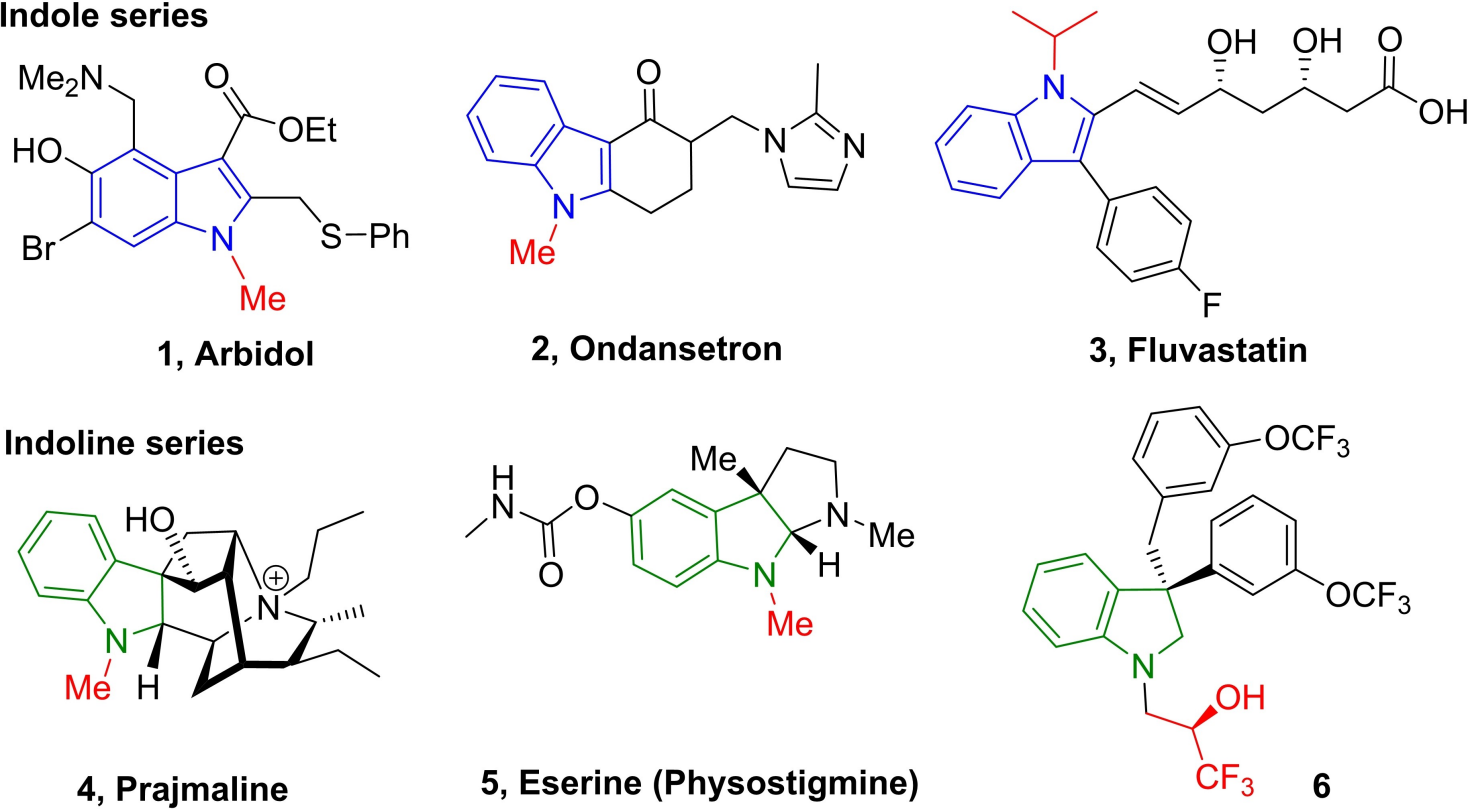
Examples of N‐alkylated indole and indoline based derivatives in drug area.

Classical alkylation of indole procedures always used alkyl halide derivatives,^[9]^ notably via a Friedel‐Crafts reaction.^[10]^ A more sustainable way is nowadays the borrowing‐hydrogen alkylation of indoles with alcohols,^[11]^ one of the crucial challenges in indole chemistry being the regioselective alkylation. Indeed, the control of the regioselectivity (N‐ vs. C3‐alkylation) is still challenging, most of the catalytic systems performing specifically the C3‐alkylation.^[12]^ At iron, the scarce described catalytic systems, mainly based on tricarbonyl(cyclopentadienone) iron complexes, performed C3‐alkylation.^[13]^ However, to the best of our knowledge, a general methodology for the N‐alkylation of indoles via BH (Borrowing‐Hydrogen) methodology has not been reported with iron catalysts. One of the main problems to orientate the selectivity towards N‐alkylation should be due to the poor nucleophilicity of the indole nitrogen center which makes difficult its reaction with the in situ generated carbonyl derivatives obtained by dehydrogenation of alcohols. Thus one alternative strategy to obtain N‐alkylated indoles can be the transformation in a two‐step, one‐pot procedure, (*i*) by performing the N‐alkylation of indolines in the presence of an alcohol, and then (*ii*) by oxidizing the resulting N‐alkylated indolines to N‐alkylindoles (Scheme 1).

**Scheme 1:**
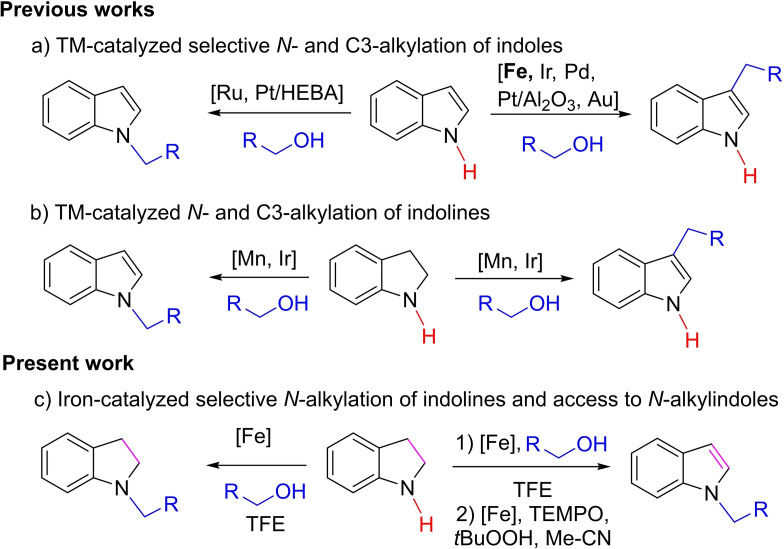
Divergent N‐ and C3‐alkylation of indoles and indolines via TM‐catalyzed borrowing‐hydrogen methodology in the presence of alcohols.

Such procedures were never reported with iron, but noticeably scarce examples were recently developed with manganese and iridium via a borrowing hydrogen/acceptorless alcohol dehydrogenation^[14]^ and transition metal catalyzed alkylation/oxidation processes involving DDQ, or MnO_2_ as final oxidant.^[15]^ Additionally, only a single example of N‐alkylation of indoline with cinnamyl alcohol leading to N‐cinnamylindoline was described in the literature at iron by B. Sundararaju.^[16]^

Based on our interest in iron catalyzed reduction and borrowing hydrogen transformations,^[17]^ we decided to investigate iron‐catalyzed N‐alkylation of indolines and the possibility to obtain in a cascade fashion N‐alkylated indoles (Scheme 1c).

We first examined the reaction of indoline **7 a** with benzyl alcohol **8 a** in the presence of 5 mol % of tricarbonyl(cyclopentadienone) iron complex **Fe‐1** and 10 mol % of Me_3_NO in toluene (Tables 1 and S1 in Supporting Information). In the presence or absence of CsOH base (0.5 equiv.) at 110 °C or even 130 °C, no reaction took place (Table 1, entries 1–3). Among the different evaluated solvents, no reaction was detected in common solvents such as cyclohexane, dioxane, or CPME (see Supporting Information) and only 3 % yield was obtained in *t*‐BuOH (Entry 4). Noticeably, when conducting the reaction in TFE, at 110 °C for 18 h, **9 a** was produced in 70 % yield (Entry 5). It should be pointed out that fluorinated solvents such as TFE were identified as original reaction media due to their unique properties such as low nucleophilicity and strong hydrogen bonding donor ability.[^14^, ^18^] In this case, TFE should favor the borrowing hydrogen reaction mainly increasing the electrophilicity of the carbonyl intermediate, thus facilitating the reaction of soft nucleophilic indolines.

**Table 1:** Optimization of the iron‐catalyzed N‐alkylation of indoline with benzyl alcohol.^[a]^

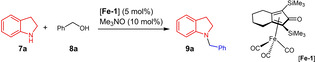				
Entry	Base (Equiv.)	Solvent	Conditions	Yield^[b]^
1	CsOH (0.5)	Toluene	110 °C, 18 h	NR
2	–	Toluene	110 °C, 18 h	NR
3	–	Toluene	130 °C, 18 h	<5
4	CsOH (0.5)	*t*‐BuOH	110 °C, 18 h	3
5	CsOH (0.5)	TFE	110 °C, 18 h	70
6	Cs_2_CO_3_ (0.5)	TFE	110 °C, 18 h	70
7	KO*t*‐Bu (0.5)	TFE	110 °C, 18 h	39
8	K_3_HPO_4_ ⋅ H_2_O (0.5)	TFE	110 °C, 18 h	80
9	K_2_CO_3_ (0.5)	TFE	110 °C, 18 h	85
**10**	**K_2_CO_3_ (1)**	**TFE**	**110 °C, 18 h**	**>99**
11^[c]^	K_2_CO_3_ (1)	TFE	110 °C, 18 h	68
12	–	TFE	110 °C, 18 h	7

[a] Experimental conditions: Indoline (0.3 mmol), benzyl alcohol (2 equiv.), Knölker catalyst **Fe‐1** (5 mol %), Me_3_NO (10 mol %), solvent (0.6 M). [b] Yields determined by ^1^H NMR using CH_2_Br_2_ as internal standard. [c] Reaction with 1.5 equiv. of BnOH. TFE: 2,2,2‐trifluoroethanol.

The screening of the base (0.5 equiv.) was then performed (see also Supporting Information). Whereas Cs_2_CO_3_ gave similar result as CsOH (Entry 6), K_2_CO_3_ was identified as the best base to perform this transformation with 85 % of **9 a** obtained by a reaction at 110 °C for 18 h (Entry 9). Finally, increasing the quantity of K_2_CO_3_ to 1 equiv. permitted to reach 99 % yield (Entry 10). Noticeably, in TFE, the base was crucial to perform the reaction as in its absence, **9 a** was detected in very low yield (7 %, entry 12). Decreasing the quantity of benzyl alcohol to 1.5 equiv. has a deleterious effect on the efficiency of the reaction, **9 a** being produced in only 68 % (Entry 11).

Using the optimized conditions (5 mol % of tricarbonyl(cyclopentadienone) iron complex **Fe‐1**, 10 mol % of Me_3_NO, 1 equiv. of K_2_CO_3_, in TFE at 110 °C for 18 h, the scope and limitation of the *N*‐alkylation of indolines were evaluated (Scheme 2).

**Scheme 2:**
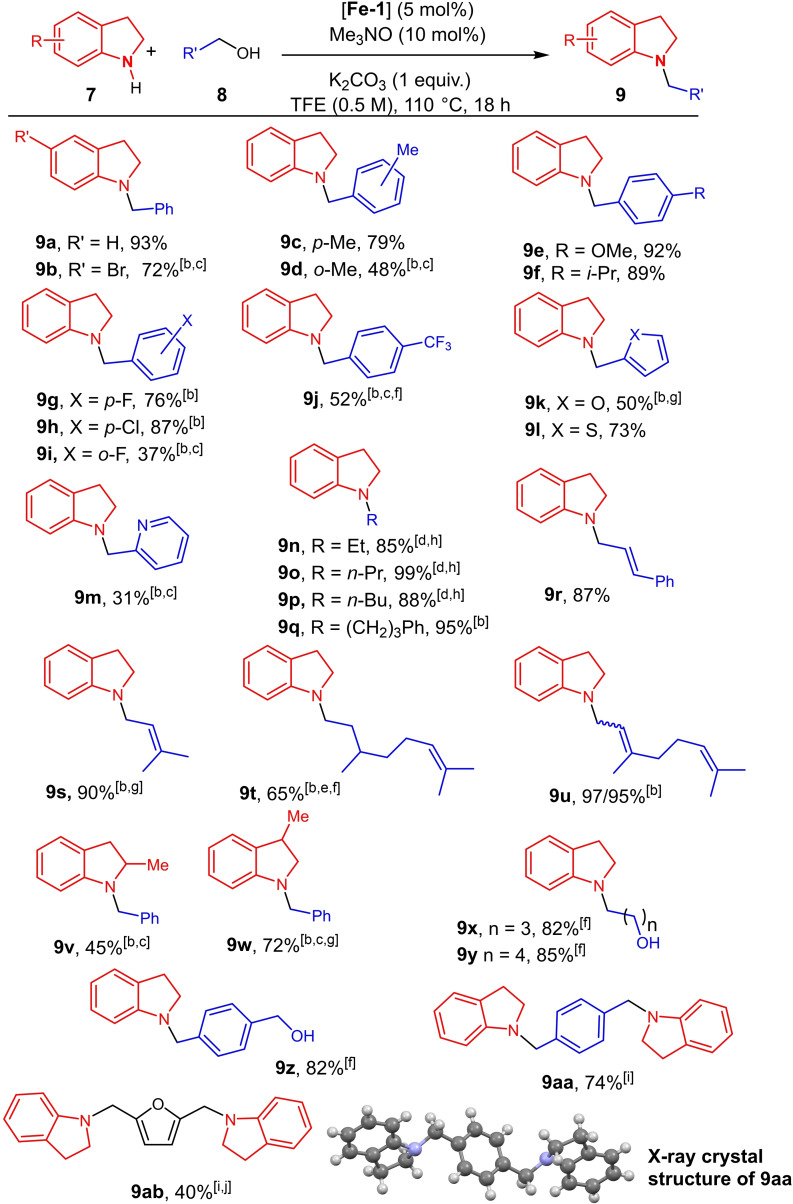
Scope of the *N*‐alkylation of indolines with alcohols. [a] Experimental conditions: Indoline (0.5 mmol), alcohol (2 equiv.), **Fe‐1** (5 mol %), Me_3_NO (10 mol %), K_2_CO_3_ (1 equiv.), TFE (0.5 M), 110 °C for 18 h under argon. Isolated yields. [b] 1 M in TFE. [c] 30 h of reaction. [d] 48 h of reaction. [e] 24 h of reaction. [f] 3 equiv. of alcohol was used. [g] 4 equiv. of alcohol was used. [h] 0.5 mL of alcohol was used. [i] 1 equiv. of diol and 3 equiv. of indole was used. [j] 7.5 mol % of **Fe‐1** and 15 mol % of Me_3_NO were used.

5‐Bromoindoline also reacted with benzyl alcohol and led after 30 h of reaction at 110 °C to the corresponding *N*‐benzyl derivative **9 b** in 72 % isolated yield. The reaction of indoline **7 a** can be conducted with benzyl alcohols bearing donating groups such as methyl, isopropyl or methoxy giving the corresponding *N*‐alkylated derivatives **9 c,e,f** in good yields up to 92 %. Fluoro‐ or chloro‐substituted benzyl alcohols were also suitable substrates for this transformation (up to 87 %, **9 g**–**h**). It must be underlined that the hindrance due to the methyl and fluoro *ortho*‐substitution of the benzyl alcohol has a significant influence on the efficiency of the transformation lowering the yields (**9 d**, 48 % and **9 i**, 37 %, respectively). When using benzyl alcohol substituted by an electron‐withdrawing group such as CF_3_ (3 equiv.), the corresponding N‐alkylated indoline **9 j** was isolated in 52 % yield after 30 h of reaction at 110 °C. It should be noted that no reaction took place with 4‐hydroxymethylbenzonitrile. Heteroaromatic alcohols such as (2‐furanyl)methanol and (2‐thienyl)methanol furnished the corresponding N‐alkylindolines in moderate yields (50 and 73 %, **9 k**–**l**). Interestingly, ethanol, *n*‐propanol and *n‐*butanol were good partners in this reaction thus conducting to the N‐alkylated indolines **9 n**–**p** in 85–99 % yields after 48 h of reaction. Additionally, 3‐phenylpropanol and (+/−)‐β‐citronellol with a remoted C=C bond led to **9 q** and **9 t** in 95 and 65 % yields, respectively. By contrast, with isopropyl alcohol, no reaction occurred. Noticeably, the reaction can be performed with allylic alcohols. Cinnamyl alcohol and prenol led to the corresponding N‐alkylated indolines **9 r**–**s** in 87–90 % yields. When starting from geraniol or nerol, the corresponding N‐alkylated indoline **9 u** was obtained in 97 and 95 % yields, respectively, but in both cases, with a mixture of (*E*)/(*Z*) allylic C=C bond. Additionally, starting from 2‐methylindoline and 3‐methylindoline, the corresponding N‐benzyl derivatives **9 v** and **9 w** were obtained in moderate yields (45 % and 72 %, respectively). We then focused our attention on the condensation of diols. Using 3 equiv. of diol under similar conditions, the monoalkylation operated and the corresponding N‐alkylated indolines **9 x**–**z** with a pending hydroxyl moiety were obtained in 82–85 % isolated yields. When using 3 equiv. of indoline **7 a** for 1 equiv. of diols, the dialkylation occurred and the corresponding N‐alkylated bis‐indolines **9 aa**–**ab** were obtained in 74 and 40 %. The X‐ray crystallographic analysis of **9 aa** further confirmed its structure and the selective N‐alkylation of indolines.

Next, we investigated the oxidation of N‐alkylated indolines in order to obtain selectively the corresponding N‐alkylated indoles (Tables 2 and S2 in Supporting Information). At iron, only few examples of efficient dehydrogenations of indolines to indoles were reported notably with heterogeneous iron on nitrogen‐doped graphene,^[19]^ well‐defined iron pincer complexes,^[20]^ FeCl_2_ in the presence of DMSO,^[21]^ or Fe(NO_3_)_3_ in the presence of TEMPO under air.^[22]^

**Table 2:** Optimization of the iron‐catalyzed one pot *N*‐alkylation of indoline/oxidation to indole.^[**a**]^

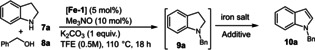					
Entry	Iron salt [mol %]	Additive (Equiv.)	Oxidant (Equiv.)	Time [h]	Yields^[b]^ **9 a/10 a**
1	–	–	TBHP (70 % aq.) (20)	2	95/<5
2	FeCl_2_ (10)	–	TBHP (70 % aq.) (3)	2	0/62(40)
3	FeCl_2_ (10)	*–*	TBHP (5.5 M) (4)	2	8/63
4	FeCl_3_ (10)	–	TBHP (5.5 M) (4)	2	9/53
5	FeBr_3_ (10)	*–*	TBHP (5.5 M) (4)	1	0/63
6	FeBr_3_ (10)	TEMPO (1)	TBHP (5.5 M) (4)	1	0/91
7	–	TEMPO (1)	–	1	88/6
8	FeBr_3_ (10)	TEMPO (1)	–	1	75/18
9	–	TEMPO (1)	TBHP (5.5 M) (4)	1	78/6
10	FeBr_3_ (10)	TEMPO (0.2)	TBHP (5.5 M) (4)	1	0/75
11	FeBr_3_ (10)	TEMPO (0.5)	TBHP (5.5 M) (4)	1	0/92
**12**	**FeBr_3_ (10)**	**TEMPO (0.5)**	**TBHP (5.5 M) (3)**	**1**	**0/91(90)**
13	FeBr_3_ (10)	TEMPO (0.5)	TBHP (5.5 M) (2)	1	0/77
14	FeBr_3_ (5)	TEMPO (0.5)	TBHP (5.5 M) (3)	1	0/70

[a] Experimental conditions: (i) Indoline (0.3 mmol), benzyl alcohol (2 equiv.), **Fe‐1** (5 mol %), Me_3_NO (10 mol %), K_2_CO_3_ (1 equiv.), TFE (0.6 M), 110 °C for 18 h under argon. The crude mixture was filtered on alumina pad and the TFE was evaporated under vacuum. (ii) Under argon, the crude mixture was then dissolved in 5 mL of CH_3_CN, and iron, additives and TBHP (5.5 M in decane) were added at RT. [b] NMR yield with dibromomethane as internal standard. TEMPO=(2,2,6,6‐tetramethylpiperidin‐1‐yl)oxyl; TBHP=*t‐*BuOOH.

In first attempts, we used classical procedures^[23]^ involving oxidants such as DDQ, MnO_2_, or peroxides (*t‐*BuOOH, etc.) which were added, after evaporation of TFE, in an acetonitrile solution. Under such conditions at RT for 2 h, indole **10 a** was not selectively produced (Table 2, entry 1 and Table S2). With the goal of obtaining a suitable procedure to dehydrogenate N‐alkyl‐indolines, we then investigated the oxidation of indolines with *t‐*BuOOH in the presence of various iron salts (Table 2 and S2). Using 10 mol % of FeCl_2_ with TBHP (70 % in aqueous solution or 5.5 M in decane), after 2 h at RT in acetonitrile, full conversion of **9 a** was observed and the benzylated indole **10 a** was obtained in up to 63 % yield (Table 2, entries 2 and 3). FeBr_3_ led to similar results (Entry 5; **10 a**, 63 %).

When using the methodology developed by Q. Wu^[22a]^ for the indoline dehydrogenation step, with 5 mol % of Fe(NO_3_)_3_ and 10 mol % of TEMPO under air in toluene at 80 °C for 16 h, only 5 % of N‐benzylindole **10 a** was detected. Nevertheless, when adding 1 equiv. of TEMPO to 5 mol % of FeBr_3_ in the presence of 4 equiv. of TBHP, a huge improvement was observed as **10 a** was selectively obtained in 91 % (Entry 6). Noticeably, with only TEMPO (1 equiv.), or when FeBr_3_ in association with TEMPO (1 equiv.) or TEMPO/THBP (1 and 4 equiv., respectively) were used, low amount of indole **10 a** (6–18 %) were detected (Entries 7–9). Decreasing the amount of TEMPO to 0.5 equiv. has no influence on the efficiency of the oxidation whereas using only 0.2 equiv. led to a significant decrease of **10 a** (Entries 10 and 11). Similarly, decreasing the amount of FeBr_3_ to 5 mol % led to only 70 % of **10 a** (Entry 14).

The quantity of TBHP was also crucial, 3 equiv. leading to the best performance with 90 % of isolated *N*‐benzylindole **10 a** (Entry 12).

With these optimized conditions in hand (5 mol % of **Fe‐1**, 10 mol % of Me_3_NO, 1 equiv. of K_2_CO_3_, in TFE (0.5 M) at 110 °C for N‐alkylation, then FeBr_3_ (10 mol %), TEMPO (50 mol %), THBP (3 equiv.), CH_3_CN, RT, 1 h) (Table 2, entry 12), the scope and limitation of the N‐alkylated indole synthesis from indolines were established (Scheme 3).

**Scheme 3:**
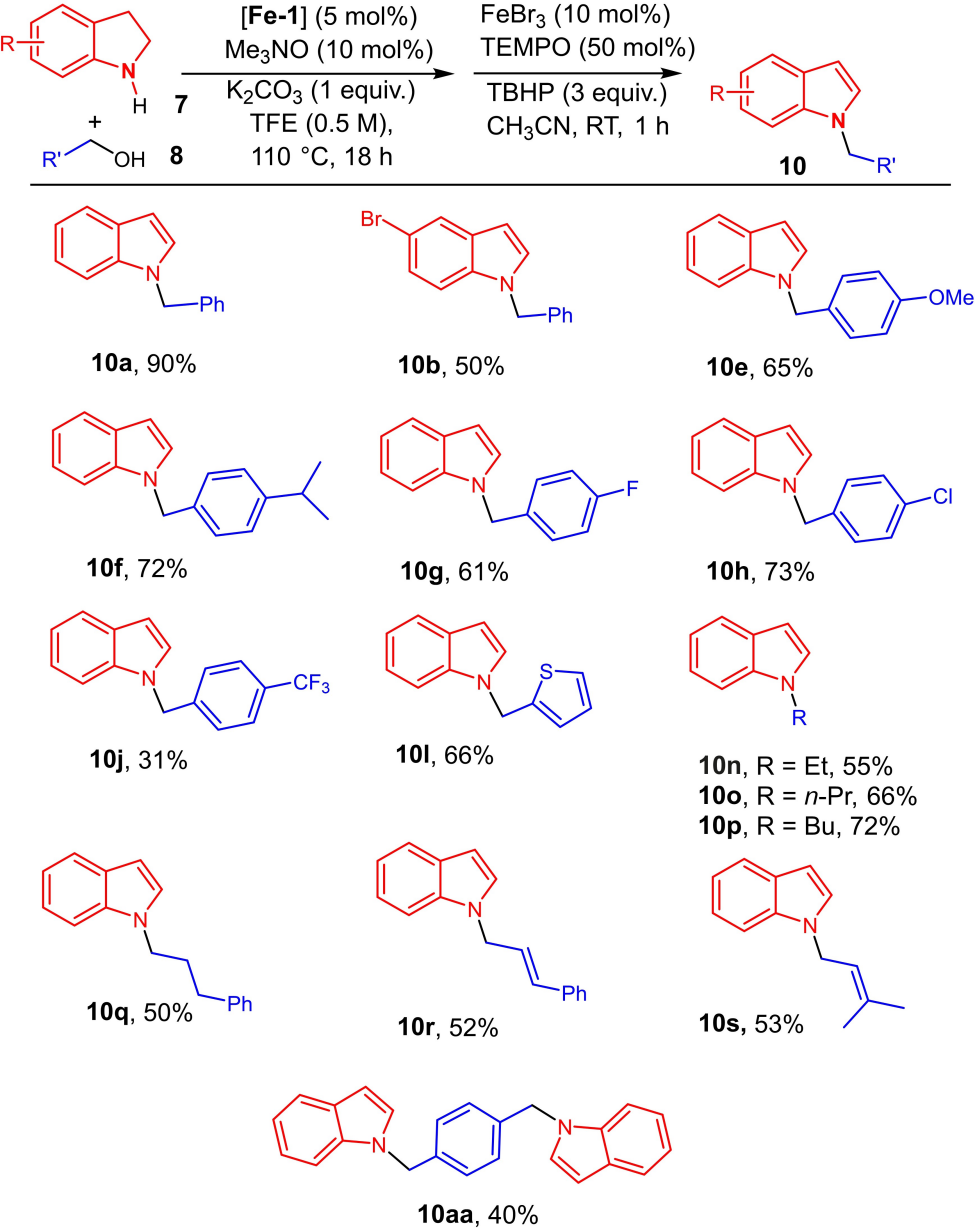
Scope of the iron‐catalyzed one pot N‐alkylation of indolines/oxidation to indoles.

Indoles bearing N‐substituted benzyl groups with electron donating groups (**10 a,b,e,f**, 50–90 % yields), and with halides (F, Cl, **10 g**–**h**, 61 and 73 % yields) can be isolated in moderate to good yields. By contrast, indole **10 j** bearing a N‐substituted benzyl groups with an electron withdrawing group such as CF_3_ was prepared with a lower yield (31 %). The reaction can be conducted with (2‐thienyl)methanol leading to the corresponding indole **10 l** in 66 % yield. When conducting the two‐step one‐pot transformation with alkanols, the corresponding N‐alkylindoles **10 n**–**q** were obtained in 50–72 % yields. Remarkably, the transformation tolerated C=C bonds as N‐allyl indole derivatives **10 r**–**s** were obtained without epoxidation of the C=C bonds. Finally, the reaction was performed with 1,4‐benzenedimethanol, and the corresponding bis‐indole **10 aa** was isolated with 40 % yield.

From a mechanistic point of view, performing the reaction with FeCl_2_ or FeCl_3_ in the presence of TBHP can suggest a radical mechanism for the dehydrogenation of indolines to indoles involving the abstraction of the α‐H atom of indoline by a butyloxy radical, obtained from TBHP by reduction by an iron salt.^[24]^ In order to check if such indoline α‐radical was produced, TEMPO was added to evaluate its scavenger ability. Surprisingly, when adding 50 mol % of TEMPO, the efficiency of the dehydrogenation was greatly improved (Table 2, entries 6 vs. 5, 91 % vs. 63 %). We thus tested several other radical scavengers (Galvinoxyl, BHT (2,6‐di‐*tert*‐butyl‐4‐methylphenol), and 1,1‐diphenylethene) under stoichiometric or catalytic conditions starting from N‐benzylindoline **9 a**, and in all case, the reaction was not inhibited (see Table S3, Supporting Information). Additionally, starting from N‐(pent‐4‐en‐1‐yl)indoline **9**–**1**, by reaction with 1 equiv. of FeBr_3_ and 1 equiv. of TEMPO at RT for 1 h, only N‐(pent‐4‐en‐1‐yl)indole **10**–**1** was obtained and no trace of cyclized derivative was observed. Finally, a clock reaction was conducted starting from N‐cyclopropylindoline **9**–**2**: under similar conditions, no ring opening derivative was observed and only the N‐cyclopropylindole **10**–**2** was detected (Table S4 and Scheme S1, Supporting Information). These experimental evidences seem to support that no indoline α‐radical was involved during the dehydrogenation process. In another hand, Fe(III) is described to be able to oxidize TEMPO to the corresponding oxoammonium **A**.^[25]^ In order to check if such oxoammonium species can oxidize indoline to indole, the reaction of N‐benzylindoline **9 a** with 1 equiv. of 4‐acetamido‐2,2,6,6‐tetramethylpiperidin‐1‐oxoammonium tetrafluoroborate salt (Bobbit's salt) in acetonitrile at RT for 1 h led to 80 % of N‐benzylindole **10 a**. Using 20 mol % of this salt in the presence of 3 equiv. of TBHP without iron catalyst, only 32 % of **10 a** was obtained whereas in the presence of 1 equiv. of FeBr_3_ without TBHP, **10 a** was detected in 19 %, showing that both FeBr_3_ and TBHP are mandatory to re‐oxidize efficiently the TEMPO−H formed *in the* oxoammonium species. Indeed, performing the reaction under conditions similar to the optimized conditions with TEMPO [**9 a** (1 equiv.), Bobbit's salt (50 mol %), FeBr_3_ (10 mol %), TBHP (3 equiv.), acetonitrile, RT, 1 h], **10 a** was obtained in 80 % yield (see Table S5, Supporting Information).

Thus, a plausible mechanism can be proposed as depicted in Scheme 4, this in situ generated oxoammonium species **A** can then react with indoline **9** leading to the iminium intermediate **C** ^[26]^ then the indole **10** by proton elimination‐isomerization.^[27]^ The obtained TEMPOH species **B** can then be re‐oxidized in oxoammonium **A** by action of Fe(III)^[28]^ which is regenerated by action of TBHP on Fe(II).^[29]^

**Scheme 4:**
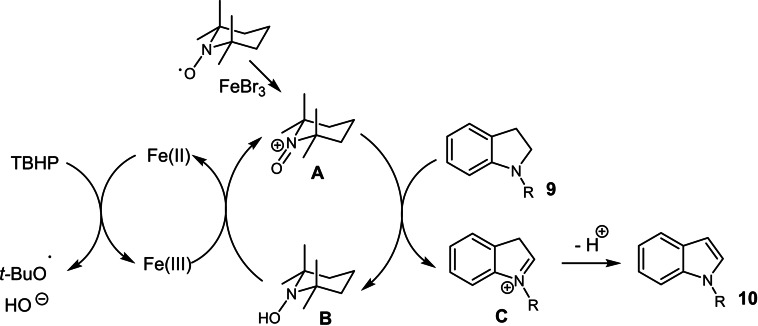
Plausible mechanism for the dehydrogenation of indolines.

In summary, we have developed a general and selective iron‐catalyzed synthesis of N‐alkylated indoles starting from the corresponding indolines via a one‐pot, two‐step procedure. Noticeably, only C3‐alkylated derivatives were selectivity obtained using iron‐catalyzed methodologies when starting from indole derivatives. This new two‐step, one‐pot procedure involves (i) the N‐alkylation of indolines via the borrowing‐hydrogen of alcohols with tricarbonyl(cyclopentadienone) iron complex as the catalyst in TFE, followed by (ii) an original selective oxidation of the obtained N‐alkylated indolines to their corresponding indole derivatives using the combination of catalytic amounts of FeBr_3_ and TEMPO in the presence of TBHP at room temperature. It should be noted that the dehydrogenation promoted by a relay iron/TEMPO catalysis did not proceed via a classical radical process. Mechanistic studies suggested that the oxidation went through the use of an oxoammonium **B** leading to an iminium intermediate **C**.

## Conflict of interest

The authors declare no conflict of interest.

## Supporting information

As a service to our authors and readers, this journal provides supporting information supplied by the authors. Such materials are peer reviewed and may be re‐organized for online delivery, but are not copy‐edited or typeset. Technical support issues arising from supporting information (other than missing files) should be addressed to the authors.

Supporting Information

## Data Availability

The data that support the findings of this study are available in the supplementary material of this article.
