# Supplementary material for: Selective Iron Catalyzed Synthesis of N‐Alkylated Indolines and Indoles
Source: Chemistry. 2022 Jul 22;28(54):e202201809. doi: 10.1002/chem.202201809 (PMC9796591; doi:10.1002/chem.202201809)
Supplement: Supplementary file 1 — Supporting Information [file CHEM-28-0-s001.pdf]

Chemistry–A European Journal

Supporting Information

Selective Iron Catalyzed Synthesis of N-Alkylated Indolines and Indoles

Jiajun Wu, Satawat Tongdee, Marie Cordier, and Christophe Darcel*

Contents

1) General information S2

2) Optimization condition for the synthesis of 1-benzylindoline **9a** S3

3) Optimization condition for the oxidation of 1-benzylindoline **9a** to 1-benzylindole **10a** S4

4) Representative procedures for the synthesis of indoline derivatives **9** and characterization data S5

5) Representative procedures for the synthesis of indole derivatives **10** and characterization data S16

6) Mechanism studies S23

7) X-ray analysis of compound **9aa** S26

8) NMR spectra S28

9) References S76

1) General information

All reagents were obtained from commercial sources and used as received. All reactions were carried out with dried glassware using standard Schlenk techniques under an inert atmosphere of dry argon. Technical grade heptane and ethyl acetate were used for column chromatography. Analytical TLC was performed on Merck 60F254 silica gel plates (0.25 mm thickness). Column chromatography was performed on Kieselgel silica gel (mesh size 40-63 μm, 60Å).

^{1}H NMR spectra were recorded in $CDCl_3$ at ambient temperature on Bruker AVANCE 400 spectrometers at 400 MHz or 500 MHz, using the solvent as the internal standard ($CDCl_3$ 7.26 ppm). ^{13}C NMR spectra were obtained at 101 MHz or 126 MHz, and referenced to the internal solvent signals ($CDCl_3$, central peak is 77.16 ppm). ^{19}F NMR spectra were obtained at 376 MHz in $CDCl_3$. Chemical shift (δ) and coupling constants (*J*) are given in ppm and in Hz respectively. The peak patterns are indicated as follows: (s, singlet; d, doublet; t, triplet; q, quartet; m, multiplet, and br. for broad).

GC-MS were measured by GCMS-QP2010S (Shimadzu) with GC-2010 equipped with a 30-m capillary column (Supelco, SLBTM-5ms, fused silica capillary column, 30 M × 0.25 mm × 0.25 mm film thickness), which was used with helium as vector gas. The following GC-MS conditions were used: initial temperature 100 °C, for 2 minutes, then rate 10 °C/min. until 250 °C and 250 °C for 10 minutes.

HR-MS spectra were carried out by using a time flight Agilent 6510 [Agilent Technologies Santa Clara (CA), USA] in an electrospray positive ionization mode at the CRMPO (Centre Régional de Mesures Physiques de l'Ouest, ScanMAT, UMS 2001 CNRS – Université de Rennes 1).

The X-ray Analysis were performed at The Centre de Diffractométrie X (CDFX) of the Insitut des Sciences Chimiques de Rennes. CCDC 2149723 contains the supplementary crystallographic data for the molecule **9aa**. These data can be obtained free of charge from The Cambridge Crystallographic Data Centre via www.ccdc.cam.ac.uk/data_request/cif.

2) Optimization conditions for the synthesis of 1-benzylindoline 9a.

Table S1- Optimization of N-alkylation of indoline with benzyl alcohol[a]

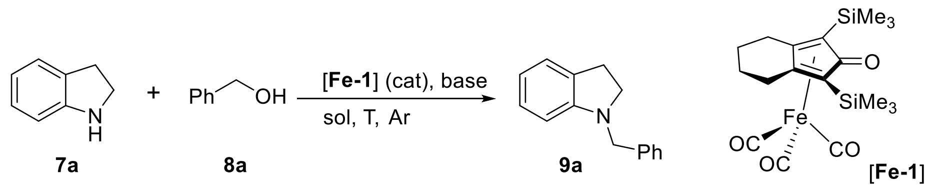

Entry	Catalyst (mol%)	8a (equiv.)	Base (equiv.)	Solvent	T (ºC)	Yield 9a (%)[b]
1	[Fe-1] (5) Me_3NO (10)	2	CsOH (0.5)	Toluene	110	NR
2	[Fe-1] (5) Me_3NO (10)	2	-	Toluene	130	<5
3	[Fe-1] (5) Me_3NO (10)	2	CsOH (0.5)	Cyclohexane	110	NR
4	[Fe-1] (5) Me_3NO (10)	2	CsOH (0.5)	CPME	110	NR
5	[Fe-1] (5) Me_3NO (10)	2	CsOH (0.5)	Dioxane	110	NR
6	[Fe-1] (5) Me_3NO (10)	2	CsOH (0.5)	*t*-BuOH	110	3
7	[Fe-1] (5) Me_3NO (10)	2	CsOH (0.5)	TFE	110	70
8	[Fe-1] (5) Me_3NO (10)	2	CsOH (0.5)	TFE	90	40
9	[Fe-1] (5) Me_3NO (10)	2	*t*-BuOK (0.5)	TFE	110	39
10	[Fe-1] (5) Me_3NO (10)	2	KOH (0.5)	TFE	110	32
11	[Fe-1] (5) Me_3NO (10)	2	$K_3PO_4 \cdot H_2O$ (0.5)	TFE	110	80
12	[Fe-1] (5) Me_3NO (10)	2	CsOAc (0.5)	TFE	110	NR
13	[Fe-1] (5) Me_3NO (10)	2	K_2CO_3 (0.5)	TFE	110	85
14	[Fe-1] (5) Me_3NO (5)	2	K_2CO_3 (0.5)	TFE	110	47
15	[Fe-1] (5) -	2	K_2CO_3 (0.5)	TFE	110	19
16	[Fe-1] (5) Me_3NO (10)	2	K_2CO_3 (0.25)	TFE	110	54
**17**	**[Fe-1] (5) Me_3NO (10)**	**2**	**K_2CO_3 (1)**	**TFE**	**110**	**>99**
18	[Fe-1] (5) Me_3NO (10)	1.5	K_2CO_3 (1)	TFE	110	68

[a] Reaction conditions: Indoline **7a** (0.3 mmol, 1 equiv.), benzyl alcohol **8a** (1.5-2 equiv.), iron catalyst **Fe-1** (0.015 mmol, 5 mol%), Me_3NO (0-10 mol%), base (0.25-1 equiv.), solvent (0.5 mL), at 90-110 ºC for 18 h under argon. [b] Yields were determined by ^1H-NMR using CH_2Br_2 as the internal standard. CPME: cyclopentyl methyl ether, TFE: trifluoroethanol.

3) Optimization conditions for the oxidation of 1-benzylindoline 9a to 1-benzylindole 10a

Table S2 - Optimization of the oxidation to N-alkylated indole[a]

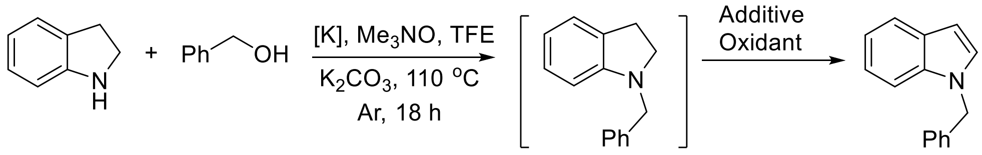

Entry	Iron salt (mol%)	Additive (equiv.)	Oxidant (equiv.)	Solvent (mL)	t (h)	Yield[b] of 9a (%)	Yield[b] of 10a (%)
1	-	-	DDQ (1)	MeCN (5)	2	45	30
2	-	-	DDQ (1.5)	MeCN (5)	2	21	26
3	-	-	MnO_2 (4)	MeCN (5)	2	93	6
4	-	-	H_2O_2 (20)	MeCN (5)	2	90	4
5	-	-	TBHP (70% aq.) (20)	MeCN (5)	2	95	<5
6	-	-	Dicumyl peroxide (3)	MeCN (5)	2	94	2
7	$FeCl_2$ (10)	-	TBHP (70% aq.) (3)	MeCN (5)	2	0	62 (40)
8	$FeCl_2$ (10)	-	TBHP (5.5 M) (4)	MeCN (5)	2	8	63
9	$Fe(OTf)_2$ (10)	-	TBHP (5.5 M) (4)	MeCN (5)	2	28	54
10	$FeCl_3$ (10)	-	TBHP (5.5 M) (4)	MeCN (5)	2	9	53
11	$FeBr_3$ (10)	-	TBHP (5.5 M) (4)	MeCN (5)	2	0	60
12	FeF_3 (10)	-	TBHP (5.5 M) (4)	MeCN (5)	2	76	trace
13	$FeBr_2$ (10)	-	TBHP (5.5 M) (4)	MeCN (5)	2	trace	51
14	$FeBr_3$ (10)	-	TBHP (5.5 M) (4)	MeCN (5)	1	0	63
15	$FeBr_3$ (10)	-	TBHP (5.5 M) (4)	THF (5)	1	53	33
16	$FeBr_3$ (10)	-	TBHP (5.5 M) (4)	Tol (5)	1	65	25
17	$FeBr_3$ (10)	-	TBHP (5.5 M) (4)	Dioxane (5)	1	59	26
18	$FeBr_3$ (10)	-	TBHP (5.5 M) (4)	Acetone (5)	1	40	40
19	$FeBr_3$ (10)	Tempo (1)	TBHP (5.5 M) (4)	MeCN (5)	1	0	91
20	-	Tempo (1)	-	MeCN (5)	1	88	6
21	$FeBr_3$ (10)	Tempo (1)	-	MeCN (5)	1	75	18
22	$FeBr_3$ (10)	Tempo (3)	-	MeCN (5)	1	70	20
23	-	Tempo (1)	TBHP (5.5 M) (4)	MeCN (5)	1	78	6
24	$FeBr_3$ (10)	Tempo (0.2)	TBHP (5.5 M) (4)	MeCN (5)	1	0	75
25	$FeBr_3$ (10)	Tempo (0.5)	TBHP (5.5 M) (4)	MeCN (5)	1	0	92
**26**	**$FeBr_3$ (10)**	**Tempo (0.5)**	**TBHP (5.5 M) (3)**	**MeCN (5)**	**1**	**0**	**91 (90)**
27	$FeBr_3$ (10)	Tempo (0.5)	TBHP (5.5 M) (2)	MeCN (5)	1	0	77
28	$FeBr_3$ (5)	Tempo (0.5)	TBHP (5.5 M) (2)	MeCN (5)	1	0	70

[a]Reaction conditions: (i) Indoline **7a** (0.3 mmol, 1 equiv.), benzyl alcohol **8a** (2 equiv.), iron catalyst (0.015 mmol, 5 mol%), Me_3NO (10 mol%), K_2CO_3 (1 equiv.), TFE (0.5 mL), at 110 °C for 18 h under argon. The crude mixture was filtered on alumina pad and the TFE was evaporated under vacuum. (ii) Under the crude mixture was then dissolved in 5 mL of solvent, and iron salt (0-10 mol%), tempo (0.2-1 equiv.) and oxidant (0-20 equiv.) were added at RT and stirred for 1-2 h. [b]Yields were determined by ^1H NMR using CH_2Br_2 as the internal standard, isolated yields were given in parenthesis.

4) Representative procedures for the synthesis of indoline derivatives and characterization data

In a dried Schlenk tube with a magnetic bar, the tricarbonyl(cyclopentadienone) iron complex [**Fe-1**] (10.2 mg, 0.025 mmol, 5 mol%), Me_3NO (3.8 mg, 0.05 mmol, 5 mol%), indoline derivative (0.5 mmol, 1 equiv.), alcohol (1-2 mmol, 2-4 equiv.), K_2CO_3 (69.1 mg, 0.5 mmol, 1 equiv.) and trifluoroethanol (TFE, 0.5-1 M) were added successively under argon atmosphere. Then, the tube was sealed and the mixture was stirred at 110 °C using an oil bath for 18-48 h. After cooling to room temperature, the resulting solution was filtrated through a pad of neutral alumina and washed with dichloromethane or ethyl acetate. The filtrate was collected and concentrated in vacuo. The residue was purified by silica gel column chromatography using ethyl acetate/heptane as the eluent to afford the desired product.

1-Benzylindoline (9a)[1]

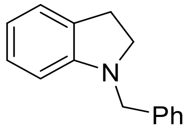

Indoline (0.5 mmol, 59.6 mg) and benzyl alcohol (1 mmol, 108.2 mg) in TFE (1 mL) for 18 h gave the title compound **9a** (97.1 mg) in 93% yield as yellow oil.

^{1}H NMR (400 MHz, $CDCl_3$) δ 7.48 – 7.28 (m, 5H), 7.21 – 7.07 (m, 2H), 6.73 (t, *J* = 7.3 Hz, 1H), 6.57 (d, *J* = 7.8 Hz, 1H), 4.31 (s, 2H), 3.36 (t, *J* = 8.3 Hz, 2H), 3.03 (t, *J* = 8.3 Hz, 2H).

^{13}C{H} NMR (101 MHz, $CDCl_3$) δ 152.7, 138.6, 130.1, 128.6, 128.0, 127.4, 127.2, 124.6, 117.8, 107.1, 53.8, 53.7, 28.7.

GC-MS: m/z (%) = 209 (M^+, 75), 132 (45), 118 (40), 91 (100), 65 (10).

1-Benzyl-5-bromoindoline (9b)[2]

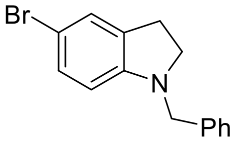

5-Bromoindoline (0.5 mmol, 99.0 mg) and benzyl alcohol (1 mmol, 108.2 mg) in TFE (0.5 mL) for 30 h gave the title compound **9b** (103.8 mg) in 72 % yield as yellow oil.

^{1}H NMR (400 MHz, $CDCl_3$) δ 7.39 – 7.27 (m, 5H), 7.22 – 7.11 (m, 2H), 6.36 (d, *J* = 8.3 Hz, 1H), 4.24 (s, 2H), 3.35 (t, *J* = 8.4 Hz, 2H), 2.97 (t, *J* = 8.4 Hz, 2H).

^{13}C{H} NMR (101 MHz, $CDCl_3$) δ 151.7, 138.0, 132.5, 130.0, 128.7, 127.9, 127.6, 127.4, 109.2, 108.2, 53.6, 53.5, 28.4.

GC-MS: m/z (%) = 289 (M^+, 35), 287 (M^+, 35), 212 (20), 210 (20), 117 (30), 91 (100), 65 (10).

1-(4'-Methylbenzyl)indoline (9c)[3]

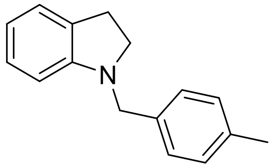

Indoline (0.5 mmol, 59.6 mg) and 4-methylbenzyl alcohol (1 mmol, 122.2 mg) in TFE (1 mL) for 18 h gave the title compound **9c** (88.1 mg) in 79 % yield as yellow oil.

^{1}H NMR (400 MHz, $CDCl_3$) δ 7.30 (d, *J* = 7.8 Hz, 2H), 7.19 (d, *J* = 7.8 Hz, 2H), 7.17 – 7.06 (m, 2H), 6.71 (t, *J* = 7.3 Hz, 1H), 6.57 (d, *J* = 7.8 Hz, 1H), 4.26 (s, 2H), 3.34 (t, *J* = 8.3 Hz, 2H), 3.01 (t, *J* = 8.3 Hz, 2H), 2.40 (s, 3H).

^{13}C{H} NMR (101 MHz, $CDCl_3$) δ 152.7, 136.8, 135.5, 130.1, 129.3, 128.0, 127.4, 124.6, 117.7, 107.2, 53.6, 53.5, 28.7, 21.2.

GC-MS: m/z (%) = 223 (M^+, 50), 132 (10), 118 (15), 105 (100), 91 (10), 77 (10), 65 (5).

1-(2'-Methylbenzyl)indoline (9d)

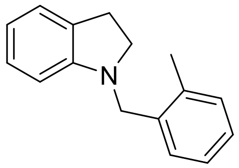

Indoline (0.5 mmol, 59.6 mg) and 2-methylbenzyl alcohol (1 mmol, 122.2 mg) in TFE (0.5 mL) for 30 h gave the title compound **9d** (53.3 mg) in 48 % yield as yellow oil.

^{1}H NMR (400 MHz, $CDCl_3$) δ 7.39 (d, *J* = 6.7 Hz, 1H), 7.27 – 7.20 (m, 3H), 7.17 – 7.08 (m, 2H), 6.72 (t, *J* = 7.3 Hz, 1H), 6.56 (d, *J* = 7.8 Hz, 1H), 4.24 (s, 2H), 3.33 (t, *J* = 8.3 Hz, 2H), 3.01 (t, *J* = 8.3 Hz, 2H), 2.42 (s, 3H).

^{13}C{H} NMR (101 MHz, $CDCl_3$) δ 152.8, 136.7, 136.4, 130.4, 130.2, 128.6, 127.5, 127.3, 126.0, 124.6, 117.7, 107.0, 53.9, 52.2, 28.7, 19.2.

HR-MS (ESI) m/z: [M+H$^+$] calcd for $C_{16}H_{18}N^+$ 224.1434; found 224.1436.

1-(4'-Methoxybenzyl)indoline (9e)[4]

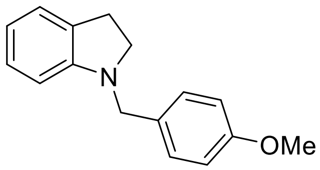

Indoline (0.5 mmol, 59.6 mg) and 4-methoxybenzyl alcohol (1 mmol, 138.2 mg) in TFE (1 mL) for 18 h gave the title compound **9e** (110.2 mg) in 92 % yield as yellow oil.

^{1}H NMR (400 MHz, CDCl$_3$) δ 7.35 (d, *J* = 8.5 Hz, 2H), 7.21 – 7.08 (m, 2H), 6.94 (d, *J* = 8.5 Hz, 2H), 6.74 (t, *J* = 7.3 Hz, 1H), 6.60 (d, *J* = 7.8 Hz, 1H), 4.25 (s, 2H), 3.86 (s, 3H), 3.34 (t, *J* = 8.3 Hz, 2H), 3.01 (t, *J* = 8.3 Hz, 2H).

^{13}C{H} NMR (101 MHz, CDCl$_3$) δ 158.9, 152.7, 130.5, 130.2, 129.2, 127.4, 124.6, 117.7, 113.9, 107.2, 55.3, 53.5, 53.1, 28.6.

GC-MS: m/z (%) = 239 (M^+, 35), 121 (100), 91 (10), 77 (10), 65 (5).

1-(4'-*Iso*-propylbenzyl)indoline (9f)

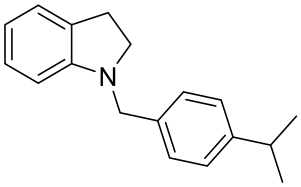

Indoline (0.5 mmol, 59.6 mg) and 4-isopropylbenzyl alcohol (1 mmol, 150.2 mg) in TFE (1 mL) for 18 h gave the title compound **9f** (111.5 mg) in 89% yield as brown oil.

^{1}H NMR (400 MHz, CDCl$_3$) δ 7.29 (d, *J* = 8.0 Hz, 2H), 7.20 (d, *J* = 8.0 Hz, 2H), 7.14 – 7.01 (m, 2H), 6.66 (t, *J* = 7.3 Hz, 1H), 6.53 (d, *J* = 7.8 Hz, 1H), 4.23 (s, 2H), 3.31 (t, *J* = 8.3 Hz, 2H), 3.03 – 2.85 (m, 3H), 1.26 (d, *J* = 6.9 Hz, 6H).

^{13}C NMR (101 MHz, CDCl$_3$) δ 152.7, 147.8, 135.9, 130.1, 128.0, 127.4, 126.6, 124.6, 117.7, 107.1, 53.6, 53.4, 33.9, 28.7, 24.2.

HR-MS (ESI) m/z: [M+H$^+$] calcd for $C_{18}H_{22}N^+$ 252.1747; found 252.1748.

1-(4'-Fluorobenzyl)indoline (9g)

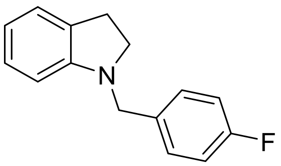

Indoline (0.5 mmol, 59.6 mg) and 4-fluorobenzyl alcohol (1 mmol, 126.1 mg) in TFE (0.5 mL) for 18 h gave the title compound **9g** (86.6 mg) in 76% yield as yellow oil.

^{1}H NMR (400 MHz, CDCl$_3$) δ 7.37 (dd, *J* = 8.5, 5.6 Hz, 2H), 7.19 – 7.01 (m, 4H), 6.73 (t, *J* = 7.2 Hz, 1H), 6.54 (d, *J* = 7.8 Hz, 1H), 4.25 (s, 2H), 3.33 (t, *J* = 8.3 Hz, 2H), 3.01 (t, *J* = 8.3 Hz, 2H).

^{13}C{H} NMR (101 MHz, CDCl$_3$) δ 162.1 (d, *J* = 244.9 Hz), 152.5, 134.3 (d, *J* = 3.2 Hz), 130.2, 129.5 (d, *J* = 7.9 Hz), 127.4, 124.7, 118.0, 115.4 (d, *J* = 21.3 Hz), 107.2, 53.7, 53.1, 28.7.

^{19}F NMR (376 MHz, CDCl$_3$) δ -115.8.

HR-MS (ESI) m/z: [M+H$^+$] calcd for $C_{15}H_{15}NF^+$ 228.1184; found 228.1185.

1-(4’-Chlorobenzyl)indoline (9h)

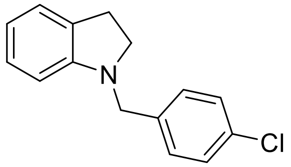

Indoline (0.5 mmol, 59.6 mg) and 4-chlorobenzyl alcohol (1 mmol, 142.6 mg) in TFE (0.5 mL) for 18 h gave the title compound **9h** (105.9 mg) in 87% yield as yellow oil.

^{1}H NMR (400 MHz, $CDCl_3$) δ 7.32 (s, 4H), 7.15 – 7.04 (m, 2H), 6.70 (t, *J* = 7.3 Hz, 1H), 6.49 (d, *J* = 7.8 Hz, 1H), 4.22 (s, 2H), 3.31 (t, *J* = 8.3 Hz, 2H), 2.99 (t, *J* = 8.3 Hz, 2H).

^{13}C{H} NMR (101 MHz, $CDCl_3$) δ 152.4, 137.2, 132.9, 130.1, 129.3, 128.8, 127.4, 124.7, 118.1, 107.2, 53.8, 53.3, 28.7.

HR-MS (ESI) m/z: [M+H$^+$] calcd for $C_{15}H_{15}N^{35}Cl^+$ 244.0888; found 244.0887.

1-(2’-Fluorobenzyl)indoline (9i)

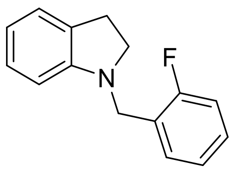

Indoline (0.5 mmol, 59.6 mg) and 2-fluorobenzyl alcohol (1 mmol, 126.1 mg) in TFE (0.5 mL) for 48 h gave the title compound **9i** (41.8 mg) in 37 % yield as brown oil.

^{1}H NMR (400 MHz, $CDCl_3$) δ 7.50 – 7.41 (m, 1H), 7.33 – 7.26 (m, 1H), 7.17 – 7.05 (m, 4H), 6.72 (t, *J* = 7.3 Hz, 1H), 6.57 (d, *J* = 7.8 Hz, 1H), 4.36 (s, 2H), 3.42 (t, *J* = 8.3 Hz, 2H), 3.03 (t, *J* = 8.3 Hz, 2H).

^{13}C NMR (101 MHz, $CDCl_3$) δ 161.1 (d, *J* = 245.9 Hz), 152.4, 130.2 (d, *J* = 4.5 Hz), 123.0, 128.8 (d, *J* = 8.1 Hz), 127.5, 125.5 (d, *J* = 14.4 Hz), 124.6, 124.2 (d, *J* = 3.6 Hz), 117.9, 115.4 (d, *J* = 21.6 Hz), 107.0, 53.8, 47.0 (d, *J* = 3.5 Hz), 28.7.

^{19}F NMR (376 MHz, $CDCl_3$) δ -118.3.

HR-MS (ESI) m/z: [M+H$^+$] calcd for $C_{15}H_{15}NF^+$ 228.1184; found 228.1184.

1-(4’-(Trifluoromethyl)benzyl)indoline (9j)[5]

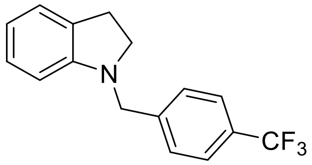

Indoline (0.5 mmol, 59.6 mg) and 4-(trifluoromethyl)benzyl alcohol (1.5 mmol, 264.2 mg) in TFE (0.5 mL) for 30 h gave the title compound **9j** (71.4 mg) in 52 % yield as yellow oil.

^{1}H NMR (400 MHz, CDCl$_3$) δ 7.64 (d, *J* = 8.1 Hz, 2H), 7.53 (d, *J* = 8.1 Hz, 2H), 7.19 – 7.08 (m, 2H), 6.75 (t, *J* = 7.3 Hz, 1H), 6.51 (d, *J* = 7.8 Hz, 1H), 4.34 (s, 2H), 3.38 (t, *J* = 8.3 Hz, 2H), 3.05 (t, *J* = 8.3 Hz, 2H).

^{13}C NMR (101 MHz, CDCl$_3$) δ 152.4, 143.0 (q, *J* = 1.2 Hz), 130.1, 129.6 (q, *J* = 32.4 Hz), 128.1, 127.5, 125.6 (q, *J* = 3.8 Hz), 124.8, 124.4 (q, *J* = 272.9 Hz), 118.2, 107.1, 54.0, 53.6, 28.7.

^{19}F NMR (376 MHz, CDCl$_3$) δ -62.3.

GC-MS: m/z (%) = 277 (M^+, 100), 258 (5), 159 (30), 132 (65), 118 (65), 91 (35), 77 (5), 65 (10).

1-(Furan-2'-ylmethyl)indoline (9k)

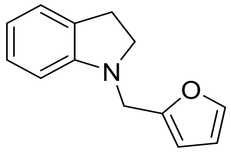

Indoline (0.5 mmol, 59.6 mg) and furfuryl alcohol (2 mmol, 196.2 mg) in TFE (0.5 mL) for 18 h gave the title compound **9k** (50.1 mg) in 50 % yield as yellow oil.

^{1}H NMR (400 MHz, CDCl$_3$) δ 7.39 (d, *J* = 1.1 Hz, 1H), 7.14 – 7.06 (m, 2H), 6.71 (t, *J* = 7.3 Hz, 1H), 6.63 (d, *J* = 8.1 Hz, 1H), 6.34 (dd, *J* = 3.1, 1.9 Hz, 1H), 6.25 (d, *J* = 2.9 Hz, 1H), 4.28 (s, 2H), 3.39 (t, *J* = 8.3 Hz, 2H), 2.98 (t, *J* = 8.3 Hz, 2H).

^{13}C{H} NMR (126 MHz, CDCl$_3$) δ 151.7, 151.6, 142.2, 130.4, 127.4, 124.6, 118.4, 110.3, 108.2, 107.8, 53.5, 46.2, 28.6.

HR-MS (ESI) m/z: [$M+H^+$] calcd for $C_{13}H_{14}NO^+$ 200.1070; found 200.1070.

1-(Thiophen-2'-ylmethyl)indoline (9l)

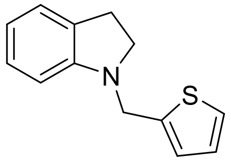

Indoline (0.5 mmol, 59.6 mg) and 2-thiophenemethanol (1 mmol, 114.2 mg) in TFE (1 mL) for 18 h gave the title compound **9l** (102.3 mg) in 73 % yield as yellow oil.

^{1}H NMR (400 MHz, CDCl$_3$) δ 7.23 (dd, *J* = 4.9, 1.0 Hz, 1H), 7.14 – 7.06 (m, 2H), 7.02 – 6.94 (m, 2H), 6.71 (t, *J* = 7.3 Hz, 1H), 6.61 (d, *J* = 7.7 Hz, 1H), 4.47 (s, 2H), 3.37 (t, *J* = 8.3 Hz, 2H), 2.98 (t, *J* = 8.3 Hz, 2H).

^{13}C{H} NMR (101 MHz, CDCl$_3$) δ 151.8, 141.4, 130.4, 127.4, 126.7, 125.8, 125.0, 124.7, 118.3, 107.7, 53.2, 48.4, 28.6.

HR-MS (ESI) m/z: [$M+H^+$] calcd for $C_{13}H_{14}NS^+$ 216.0842; found 216.0843.

1-(Pyridin-2'-ylmethyl)indoline (9m)[6]

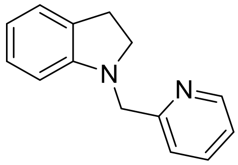

Indoline (0.5 mmol, 59.6 mg) and 2-pyridinemethanol (1 mmol, 109.1 mg) in TFE (0.5 mL) for 30 h gave the title compound **9m** (32.5 mg) in 31 % yield as brown oil.

^{1}H NMR (400 MHz, $CDCl_3$) δ 8.60 (d, *J* = 4.3 Hz, 1H), 7.65 (td, *J* = 7.7, 1.2 Hz, 1H), 7.43 (d, *J* = 7.8 Hz, 1H), 7.19 (dd, *J* = 6.7, 5.4 Hz, 1H), 7.12 (d, *J* = 7.2 Hz, 1H), 7.05 (t, *J* = 7.6 Hz, 1H), 6.69 (t, *J* = 7.3 Hz, 1H), 6.46 (d, *J* = 7.8 Hz, 1H), 4.41 (s, 2H), 3.46 (t, *J* = 8.3 Hz, 2H), 3.04 (t, *J* = 8.3 Hz, 2H).

^{13}C NMR (101 MHz, $CDCl_3$) δ 159.0, 152.4, 149.4, 136.8, 129.9, 127.4, 124.6, 122.2, 121.9, 117.9, 107.1, 55.8, 54.2, 28.8.

GC-MS: m/z (%) = 210 (M^+, 10), 132 (5), 117 (10), 93 (100), 77 (5), 65 (10).

1-Ethylindoline (9n)[7]

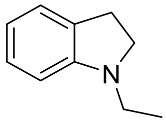

Indoline (0.5 mmol, 59.6 mg) and ethanol (0.5 mL) in TFE (1 mL) for 48 h gave the title compound **9n** (62.5 mg) in 85 % yield as yellow oil.

^{1}H NMR (400 MHz, $CDCl_3$) δ 7.16 – 7.06 (m, 2H), 6.69 (t, *J* = 7.3 Hz, 1H), 6.53 (d, *J* = 7.7 Hz, 1H), 3.37 (t, *J* = 8.3 Hz, 2H), 3.18 (q, *J* = 7.2 Hz, 2H), 2.99 (t, *J* = 8.3 Hz, 2H), 1.24 (t, *J* = 7.2 Hz, 3H).

^{13}C{H} NMR (101 MHz, $CDCl_3$) δ 152.5, 130.4, 127.3, 124.5, 117.6, 107.3, 52.4, 43.2, 28.6, 12.1.

GC-MS: m/z (%) = 147 (M^+, 45), 132 (100), 117 (25), 91 (10), 77 (10), 65 (10), 51 (5).

1-Propylindoline (9o)

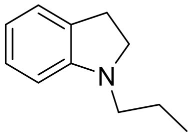

Indoline (0.5 mmol, 59.6 mg) and 1-propanol (0.5 mL) in TFE (1 mL) for 48 h gave the title compound **9o** (79.6 mg) in 99 % yield as yellow oil.
CAS: 17274-60-1

^{1}H NMR (400 MHz, $CDCl_3$) δ 7.14 – 7.01 (m, 2H), 6.63 (t, *J* = 7.3 Hz, 1H), 6.47 (d, *J* = 7.8 Hz, 1H), 3.35 (t, *J* = 8.3 Hz, 2H), 3.05 – 2.94 (m, 4H), 1.69 – 1.59 (m, 2H), 0.99 (t, *J* = 7.4 Hz, 3H).

^{13}C{H} NMR (101 MHz, $CDCl_3$) δ 152.9, 130.1, 127.4, 124.5, 117.4, 107.0, 53.2, 51.3, 28.7, 20.8, 11.9.

GC-MS: m/z (%) = 161 (M^+, 25), 132 (100), 117 (20), 91 (5), 77 (5).

1-Butylindoline (9p)[1]

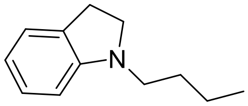

Indoline (0.5 mmol, 59.6 mg) and 1-butanol (0.5 mL) in TFE (1 mL) for 48 h gave the title compound **9p** (77.0 mg) in 88 % yield as yellow oil.

^{1}H NMR (400 MHz, $CDCl_3$) δ 7.15 – 7.01 (m, 2H), 6.64 (t, *J* = 7.3 Hz, 1H), 6.48 (d, *J* = 7.9 Hz, 1H), 3.36 (t, *J* = 8.3 Hz, 2H), 3.07 (t, *J* = 7.6 Hz, 2H), 2.97 (t, *J* = 8.3 Hz, 2H), 1.65 – 1.57 (m, 2H), 1.49 – 1.39 (m, 2H), 0.99 (t, *J* = 7.3 Hz, 3H).

^{13}C{H} NMR (101 MHz, $CDCl_3$) δ 152.9, 130.1, 127.4, 124.5, 117.3, 106.9, 53.2, 49.1, 29.6, 28.7, 20.6, 14.1.

GC-MS: m/z (%) = 175 (M^+, 20), 132 (100), 117 (15), 91 (5), 77 (5).

1-(3'-Phenylpro-1'-pyl)indoline (9q)[8]

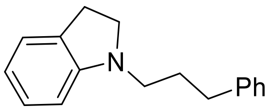

Indoline (0.5 mmol, 59.6 mg) and 3-phenylpropanol (1 mmol, 136.2 mg) in TFE (0.5 mL) for 18 h gave the title compound **9q** (112.5 mg) in 95 % yield as yellow oil.

^{1}H NMR (400 MHz, $CDCl_3$) δ 7.36 – 7.31 (m, 2H), 7.28 – 7.20 (m, 3H), 7.15 – 7.05 (m, 2H), 6.68 (t, *J* = 7.3 Hz, 1H), 6.45 (d, *J* = 7.8 Hz, 1H), 3.38 (t, *J* = 8.3 Hz, 2H), 3.11 (t, *J* = 7.2 Hz, 2H), 3.00 (t, *J* = 8.3 Hz, 2H), 2.77 (t, *J* = 7.6 Hz, 2H), 2.03 – 1.92 (m, 2H).

^{13}C{H} NMR (101 MHz, $CDCl_3$) δ 152.8, 142.0, 130.1, 128.6, 128.5, 127.4, 126.0, 124.5, 117.6, 107.1, 53.2, 48.8, 33.4, 29.2, 28.7.

GC-MS: m/z (%) = 237 (M^+, 15), 132 (100), 117 (15), 91 (10), 65 (5).

1-Cinnamylindoline (9r)[9]

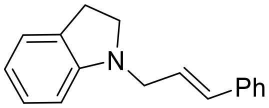

Indoline (0.5 mmol, 59.6 mg) and cinnamyl alcohol (1 mmol, 134.2 mg) in TFE (1 mL) for 18 h gave the title compound **9r** (102.3 mg) in 87 % yield as yellow oil.

^{1}H NMR (400 MHz, $CDCl_3$) δ 7.45 (d, *J* = 7.5 Hz, 2H), 7.38 (t, *J* = 7.5 Hz, 2H), 7.30 (t, *J* = 7.4 Hz, 1H), 7.19 – 7.12 (m, 2H), 6.75 (t, *J* = 7.4 Hz, 1H), 6.70 (d, *J* = 15.8 Hz, 1H), 6.64 (d, *J* = 7.8 Hz, 1H), 6.37 (dt, *J* = 15.8, 6.1 Hz, 1H), 3.94 (d, *J* = 6.1 Hz, 2H), 3.45 (t, *J* = 8.3 Hz, 2H), 3.04 (t, *J* = 8.3 Hz, 2H).

^{13}C{H} NMR (101 MHz, $CDCl_3$) δ 152.3, 137.0, 132.4, 130.4, 128.7, 127.6, 127.4, 126.5, 126.0, 124.6, 117.9, 107.5, 53.4, 51.7, 28.7.

GC-MS: m/z (%) = 235 (M^+, 50), 144 (15), 117 (100), 91 (25), 65 (10).

1-(3-Methylbut-2-en-1-yl)indoline (9s)[10]

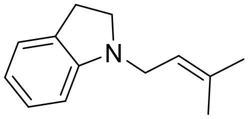

Indoline (0.5 mmol, 59.6 mg) and 3-methyl-2-buten-1-ol (2 mmol, 172.3 mg) in TFE (0.5 mL) for 18 h gave the title compound **9s** (84.5 mg) in 90 % yield as yellow oil.

^{1}H NMR (400 MHz, $CDCl_3$) δ 7.14 – 7.06 (m, 2H), 6.68 (t, *J* = 7.5 Hz, 1H), 6.55 (d, *J* = 7.7 Hz, 1H), 5.39 – 5.30 (m, 1H), 3.72 (d, *J* = 6.8 Hz, 2H), 3.34 (t, *J* = 8.2 Hz, 2H), 2.96 (t, *J* = 8.2 Hz, 2H), 1.78 (d, *J* = 0.7 Hz, 3H), 1.76 (s, 3H).

^{13}C NMR (101 MHz, $CDCl_3$) δ 152.6, 135.4, 130.6, 127.3, 124.5, 120.3, 117.7, 107.5, 53.2, 46.8, 28.7, 25.9, 18.1.

GC-MS: m/z (%) = 187 (M^+, 60), 172 (10), 132 (10), 119 (100), 91 (20), 69 (25).

1-(3’,7’-Dimethyloct-6’-en-1’-yl)indoline (9t)

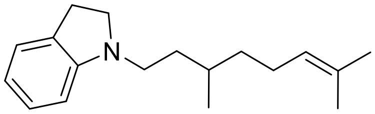

Indoline (0.5 mmol, 59.6 mg) and citronellol (1.5 mmol, 234.4 mg) in TFE (0.5 mL) for 24 h gave the title compound **9t** (83.2 mg) in 65 % yield as yellow oil.

^{1}H NMR (400 MHz, $CDCl_3$) δ 7.12 – 7.05 (m, 2H), 6.65 (t, *J* = 7.3 Hz, 1H), 6.49 (d, *J* = 8.0 Hz, 1H), 5.20 – 5.09 (m, 1H), 3.41 – 3.30 (m, 2H), 3.10 (t, *J* = 7.6 Hz, 2H), 2.97 (t, *J* = 8.3 Hz, 2H), 2.11 – 1.97 (m, 2H), 1.72 (s, 3H), 1.68 – 1.55 (m, 5H), 1.48 – 1.38 (m, 2H), 1.30 – 1.22 (m, 1H), 0.99 (d, *J* = 6.5 Hz, 3H).

^{13}C NMR (101 MHz, $CDCl_3$) δ 152.8, 131.4, 130.2, 127.4, 124.9, 124.5, 117.4, 107.0, 53.1, 47.3, 37.3, 34.2, 30.7, 28.7, 25.9, 25.6, 19.8, 17.8.

HR-MS (ESI) m/z: [$M+H^+$] calcd for $C_{18}H_{28}N^+$ 258.2217; found 258.2216.

1-(3’,7’-Dimethylocta-2’,6’-dien-1’-yl)indoline (9u)

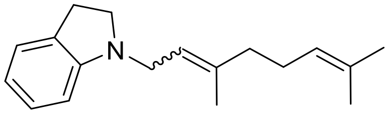

Indoline (0.5 mmol, 59.6 mg) and geraniol (1 mmol, 154.3 mg) in TFE (0.5 mL) for 18 h gave the title compound **9u** (124.0 mg) in 97 % yield as yellow oil as a 1:1 mixture of (*E*)/(*Z*) isomers.

Indoline (0.5 mmol, 59.6 mg) and nerol (1 mmol, 154.3 mg) in TFE (0.5 mL) for 18 h gave the title compound **9u** (121.5 mg) in 95 % yield as yellow oil as a 1:1 mixture of (*E*)/(*Z*) isomers.

^{1}H NMR (500 MHz, $CDCl_3$) δ 7.20 – 7.04 (m, 2H), 6.77 – 6.66 (m, 1H), 6.62 – 6.52 (m, 1H), 5.42 – 5.32 (m, 1H), 5.22 – 5.09 (m, 1H), 3.75 (d, *J* = 6.8 Hz, 2H), 3.35 (td, *J* = 8.3, 3.1 Hz, 2H), 2.97 (t, *J* = 8.3 Hz, 2H), 2.21 – 2.08 (m, 4H), 1.80 – 1.64 (m, 9H).

^{13}C NMR (126 MHz, $CDCl_3$) observation of 2 diastereoisomers: δ 152.6, 152.5, 139.0, 138.8, 132.0, 131.6, 130.5, 130.5, 127.3, 124.5, 124.5, 124.2, 124.1, 121.1, 120.3, 117.7, 117.7, 107.6, 107.5, 53.2, 46.9, 46.6, 39.8, 32.3, 28.7, 26.6, 26.6, 25.9, 25.8, 23.5, 17.8, 17.8, 16.4.

HR-MS (ESI) m/z: [M+H$^+$] calcd for $C_{18}H_{26}N^+$, 256.2060; found 256.2059.

1-Benzyl-2-methylindoline (9v)[11]

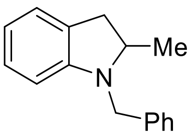

2-Methylindoline (0.5 mmol, 66.6 mg) and benzyl alcohol (1 mmol, 108.2 mg) in TFE (0.5 mL) for 30 h gave the title compound **9v** (50.2 mg) in 45 % yield as yellow oil.

^{1}H NMR (400 MHz, $CDCl_3$) δ 7.45 – 7.35 (m, 4H), 7.33 – 7.27 (m, 1H), 7.14 – 7.02 (m, 2H), 6.69 (t, *J* = 7.3 Hz, 1H), 6.38 (d, *J* = 7.8 Hz, 1H), 4.42 (d, *J* = 16.1 Hz, 1H), 4.25 (d, *J* = 16.1 Hz, 1H), 3.85 – 3.73 (m, 1H), 3.23 (dd, *J* = 15.6, 8.6 Hz, 1H), 2.74 (dd, *J* = 15.6, 9.7 Hz, 1H), 1.36 (d, *J* = 6.1 Hz, 3H).

^{13}C NMR (101 MHz, $CDCl_3$) δ 152.8, 139.4, 128.9, 128.6, 127.5, 127.4, 127.0, 124.2, 117.5, 106.9, 60.7, 51.3, 37.5, 19.8.

GC-MS: m/z (%) = 223 (M$^+$, 40), 208 (50), 130 (10), 117 (10), 91 (100), 65 (15).

1-Benzyl-3-methylindoline (9w)[12]

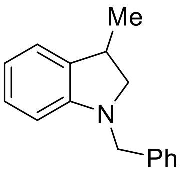

3-Methylindoline (0.5 mmol, 66.6 mg) and benzyl alcohol (2 mmol, 216.4 mg) in TFE (0.5 mL) for 30 h gave the title compound **9w** (79.8 mg) in 72 % yield as yellow oil.

^{1}H NMR (400 MHz, $CDCl_3$) δ 7.48 – 7.28 (m, 5H), 7.16 – 7.07 (m, 2H), 6.75 (t, *J* = 7.4 Hz, 1H), 6.56 (d, *J* = 8.1 Hz, 1H), 4.41 (d, *J* = 14.8 Hz, 1H), 4.17 (d, *J* = 14.8 Hz, 1H), 3.56 (t, *J* = 8.6 Hz, 1H), 3.41 – 3.27 (m, 1H), 2.88 (t, *J* = 8.6 Hz, 1H), 1.35 (d, *J* = 6.8, 3H).

^{13}C NMR (101 MHz, $CDCl_3$) δ 152.2, 138.6, 135.1, 128.6, 128.0, 127.5, 127.2, 123.3, 117.9, 107.1, 61.7, 53.4, 35.3, 18.8.

GC-MS: m/z (%) = 223 (M$^+$, 45), 208 (20), 146 (10), 132 (15), 117 (10), 91 (100), 77 (10), 65 (15).

4-(Indolin-1'-yl)butan-1-ol (9x)[13]

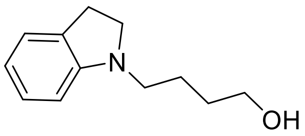

Indoline (0.5 mmol, 59.6 mg) and 1,4-butandiol (1.5 mmol, 135.2 mg) in TFE (0.5 mL) for 18 h gave the title compound **9x** (78.4 mg) in 82 % yield as yellow oil.

^{1}H NMR (400 MHz, $CDCl_3$) δ 7.17 – 6.99 (m, 2H), 6.68 (t, *J* = 7.3 Hz, 1H), 6.52 (d, *J* = 7.7 Hz, 1H), 3.68 (t, *J* = 5.7 Hz, 2H), 3.34 (t, *J* = 8.3 Hz, 2H), 3.09 (t, *J* = 6.5 Hz, 2H), 2.97 (t, *J* = 8.3 Hz, 2H), 2.19 (br, 1H), 1.79 – 1.63 (m, 4H).

^{13}C NMR (101 MHz, $CDCl_3$) δ 152.6, 130.3, 127.4, 124.5, 118.0, 107.5, 62.8, 53.4, 49.7, 30.8, 28.7, 24.3.

GC-MS: m/z (%) = 191 (M^+, 15), 132 (100), 117 (15), 91 (5), 77 (5).

5-(Indolin-1-yl)pentan-1-ol (9y)

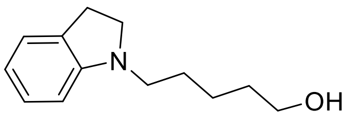

Indoline (0.5 mmol, 59.6 mg) and 1,5-pentandiol (1.5 mmol, 156.2 mg) in TFE (0.5 mL) for 18 h gave the title compound **9y** (87.3 mg) in 85 % yield as yellow oil.

^{1}H NMR (400 MHz, $CDCl_3$) δ 7.16 – 6.99 (m, 2H), 6.65 (t, *J* = 7.3 Hz, 1H), 6.48 (d, *J* = 7.7 Hz, 1H), 3.66 (t, *J* = 6.5 Hz, 2H), 3.34 (t, *J* = 8.3 Hz, 2H), 3.07 (t, *J* = 7.2 Hz, 2H), 2.97 (t, *J* = 8.3 Hz, 2H), 1.71 – 1.58 (m, 5H), 1.52 – 1.42 (m, 2H).

^{13}C NMR (101 MHz, $CDCl_3$) δ 152.8, 130.1, 127.4, 124.5, 117.5, 107.0, 62.9, 53.2, 49.4, 32.7, 28.7, 27.3, 23.6.

HR-MS (ESI) m/z: [$M+Na^+$] calcd for $C_{13}H_{19}NONa^+$ 228.1359; found 228.1361.

(4-(Indolin-1'-ylmethyl)phenyl)methanol (9z)

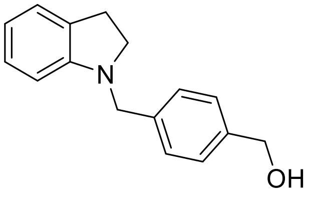

Indoline (0.5 mmol, 59.6 mg) and 1,4-phenylenedimethanol (1.5 mmol, 207.2 mg) in TFE (0.5 mL) for 18 h gave the title compound **9z** (97.6 mg) in 82 % yield as yellow oil.

^{1}H NMR (400 MHz, $CDCl_3$) δ 7.39 (d, *J* = 8.1 Hz, 2H), 7.35 (d, *J* = 8.1 Hz, 2H), 7.17 – 7.02 (m, 2H), 6.71 (t, *J* = 7.3 Hz, 1H), 6.54 (d, *J* = 7.8 Hz, 1H), 4.68 (s, 2H), 4.27 (s, 2H), 3.33 (t, *J* = 8.3 Hz, 2H), 2.99 (t, *J* = 8.3 Hz, 2H), 2.00 (br, 1H).

^{13}C NMR (101 MHz, $CDCl_3$) δ 152.6, 139.9, 138.0, 130.1, 128.2, 127.4, 127.3, 124.6, 117.9, 107.2, 65.2, 53.7, 53.5, 28.6.

HR-MS (ESI) m/z: [M+Na$^+$] calcd for $C_{16}H_{17}NONa^+$ 262.1202; found 262.1204.

1,4-Bis(indolin-1'-ylmethyl)benzene (9aa)

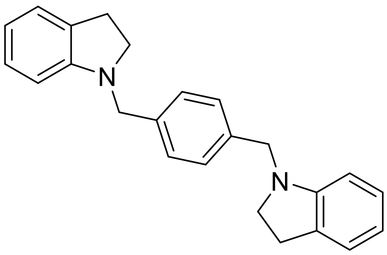

Indoline (1.5 mmol, 178.7 mg) and 1,4-phenylenedimethanol (0.5 mmol, 69.1 mg) in TFE (0.5 mL) for 18 h gave the title compound **9aa** (125.5 mg) in 74 % yield as white solid.

^{1}H NMR (400 MHz, $CDCl_3$) δ 7.34 (s, 4H), 7.16 – 7.02 (m, 4H), 6.68 (t, *J* = 7.3 Hz, 2H), 6.53 (d, *J* = 7.8 Hz, 2H), 4.26 (s, 4H), 3.33 (t, *J* = 8.3 Hz, 4H), 2.98 (t, *J* = 8.3 Hz, 4H).

^{13}C NMR (101 MHz, $CDCl_3$) δ 152.7, 137.5, 130.2, 128.2, 127.4, 124.6, 117.8, 107.2, 53.7, 53.5, 28.7.

HR-MS (ESI) m/z: [M+H$^+$] calcd for $C_{24}H_{25}N_2^+$, 341.2013; found 341.2012.

2,5-Bis(indolin-1'-ylmethyl)furan (9ab)

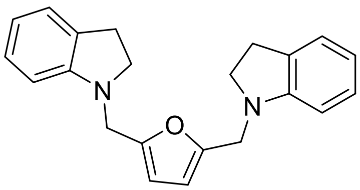

Indoline (1.5 mmol, 178.7 mg) and 2,5-furandimethanol (0.5 mmol, 64.1 mg) in TFE (0.5 mL) for 30 h gave the title compound **9ab** (66.0 mg) in 40 % yield as brown oil.

^{1}H NMR (400 MHz, $CDCl_3$) δ 7.14 – 7.04 (m, 4H), 6.69 (t, *J* = 7.3 Hz, 2H), 6.60 (d, *J* = 7.7 Hz, 2H), 6.15 (s, 2H), 4.23 (s, 4H), 3.34 (t, *J* = 8.3 Hz, 4H), 2.95 (t, *J* = 8.3 Hz, 4H).

^{13}C NMR (101 MHz, $CDCl_3$) δ 151.8, 151.3, 130.3, 127.3, 124.6, 118.1, 108.8, 107.7, 53.5, 46.1, 28.6.

HR-MS (ESI) m/z: [M+Na$^+$] calcd for $C_{22}H_{22}N_2ONa^+$ 353.1625; found 353.1628.

5) Representative procedures for the synthesis of indole derivatives and characterization data

In a dried Schlenk tube with a magnetic bar, the tricarbonyl(cyclopentadienone) iron complex [**Fe-1**] (10.2 mg, 0.025 mmol, 5 mol%), Me_3NO (3.8 mg, 0.05 mmol, 5 mol%), indoline derivative (0.5 mmol, 1 equiv.), alcohol (1-2 mmol, 2-4 equiv.), K_2CO_3 (69.1 mg, 0.5 mmol, 1 equiv.) and trifluoroethanol (TFE, 0.5-1 M) were added successively under argon atmosphere. Then, the tube was sealed and the mixture was stirred at 110 °C using an oil bath for 18-48 h. After cooling to room temperature, the mixture was filtered through a pad of neutral alumina and transferred into a clean Schlenk tube without solvent. Then, $FeBr_3$ (14.9 mg, 0.1 mmol, 10 mol%), TEMPO (0.25 mmol, 0.5 equiv.), *t*-BuOOH (1.5 mmol, 3 equiv.) and MeCN (6 mL) were added under argon and stirred for 1 h at room temperature. After the oxidation reaction, the mixture was filtered again through a pad of neutral alumina oxide and washed with dichloromethane, and then the crude product was purified by silica gel column chromatography using dichloromethane/heptane as the eluent to afford the desired product.

1-Benzyl-1*H*-indole (10a)[8]

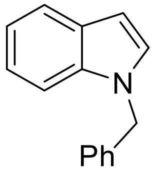

Indoline (0.5 mmol, 59.6 mg) and benzyl alcohol (1 mmol, 108.2 mg) in TFE (1 mL) for 18 h, then the title compound **10a** (93.0 mg) can be obtained under oxidation conditions in 90% yield as colorless oil.

^{1}H NMR (400 MHz, $CDCl_3$) δ 7.69 (d, *J* = 7.8 Hz, 1H), 7.38 – 7.27 (m, 4H), 7.23 – 7.11 (m, 5H), 6.63 – 6.54 (m, 1H), 5.35 (s, 2H).

^{13}C NMR (101 MHz, $CDCl_3$) δ 137.7, 136.4, 128.9, 128.9, 128.4, 127.7, 126.9, 121.8, 121.1, 119.7, 109.8, 101.8, 50.2.

GC-MS m/z (%): 207 (M^+, 45), 91(100), 65 (15).

1-Benzyl-5-bromo-1*H*-indole (10b)[8]

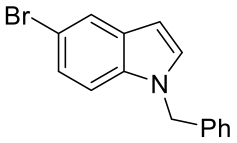

5-Bromoindoline (0.5 mmol, 99.0 mg) and benzyl alcohol (1 mmol, 108.2 mg) in TFE (0.5 mL) for 30 h, then the title compound **10b** (71.8 mg) can be obtained under oxidation conditions in 50% yield as yellow solid.

^{1}H NMR (400 MHz, $CDCl_3$) δ 7.80 (d, *J* = 1.5 Hz, 1H), 7.36 – 7.25 (m, 4H), 7.18 – 7.13 (m, 2H), 7.13 – 7.05 (m, 2H), 6.52 (d, *J* = 3.1 Hz, 1H), 5.30 (s, 2H).

^{13}C NMR (101 MHz, $CDCl_3$) δ 137.1, 135.1, 130.5, 129.6, 129.0, 127.9, 126.8, 124.7, 123.6, 113.0, 111.3, 101.4, 50.4.

GC-MS m/z (%): 287 (M^+, 30), 285 (M^+, 30), 204 (5), 115 (5), 103 (5), 91 (100), 65 (10).

1-(4'-Methoxybenzyl)-1*H*-indole (10e)[8]

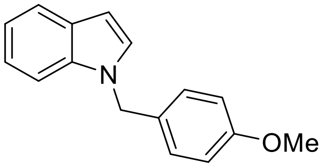

Indoline (0.5 mmol, 59.6 mg) and 4-methoxybenzyl alcohol (1 mmol, 138.2 mg) in TFE (1 mL) for 18 h, then the title compound **10e** (76.8 mg) can be obtained under oxidation conditions in 65% yield as colorless oil.

^{1}H NMR (400 MHz, $CDCl_3$) δ 7.68 (d, *J* = 7.6 Hz, 1H), 7.33 (d, *J* = 8.4 Hz, 1H), 7.23 – 7.06 (m, 5H), 6.85 (d, *J* = 8.4 Hz, 2H), 6.62 – 6.52 (m, 1H), 5.27 (s, 2H), 3.79 (s, 3H).

^{13}C NMR (101 MHz, $CDCl_3$) δ 159.2, 136.4, 129.6, 128.9, 128.3, 128.2, 121.7, 121.1, 119.6, 114.2, 109.8, 101.6, 55.4, 49.7.

GC-MS m/z (%): 237 (M^+, 20), 121 (100), 91 (5), 77 (10).

1-(4'-*Iso*-propylbenzyl)-1*H*-indole (10f)

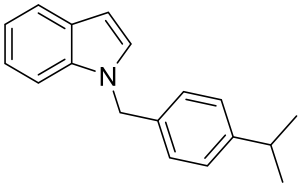

Indoline (0.5 mmol, 59.6 mg) and 4-isopropylbenzyl alcohol (1 mmol, 150.2 mg) in TFE (1 mL) for 18 h, then the title compound **10f** (89.8 mg) can be obtained under oxidation conditions in 72% yield as yellow oil.

CAS: 1225754-08-4

^{1}H NMR (400 MHz, $CDCl_3$) δ 7.68 (d, *J* = 7.6 Hz, 1H), 7.34 (d, *J* = 8.4 Hz, 1H), 7.24 – 7.01 (m, 7H), 6.64 – 6.49 (m, 1H), 5.31 (s, 2H), 2.98 – 2.80 (m, 1H), 1.25 (d, *J* = 6.9 Hz, 6H).

^{13}C NMR (101 MHz, $CDCl_3$) δ 148.4, 136.5, 135.0, 128.8, 128.3, 127.0, 126.9, 121.7, 121.1, 119.6, 109.8, 101.7, 50.0, 33.9, 24.1.

GC-MS m/z (%): 249 (M^+, 35), 133 (100), 117 (25), 105 (25), 91 (15), 77 (5).

1-(4'-Fluorobenzyl)-1*H*-indole (10g)[14]

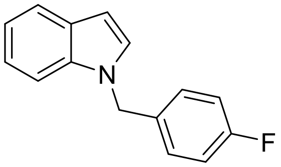

Indoline (0.5 mmol, 59.6 mg) and 4-fluorobenzyl alcohol (1 mmol, 126.1 mg) in TFE (0.5 mL) for 18 h, then the title compound **10g** (68.5 mg) can be obtained under oxidation conditions in 61% yield as colorless oil.

^{1}H NMR (400 MHz, CDCl$_3$) δ 7.73 (d, *J* = 7.7 Hz, 1H), 7.31 (d, *J* = 8.1 Hz, 1H), 7.27 – 7.09 (m, 5H), 7.06 – 6.97 (m, 2H), 6.62 (d, *J* = 2.8 Hz, 1H), 5.30 (s, 2H).

^{13}C NMR (101 MHz, CDCl$_3$) δ 162.3 (d, *J* = 246.0 Hz), 136.3, 133.4 (d, *J* = 3.2 Hz), 128.9, 128.5 (d, *J* = 8.2 Hz), 128.2, 121.9, 121.2, 119.8, 115.7 (d, *J* = 21.6 Hz), 109.7, 102.0, 49.5.

^{19}F NMR (376 MHz, CDCl$_3$) δ -114.8.

GC-MS m/z (%): 225 (M$^+$, 35), 109 (100), 83 (15), 63 (5).

1-(4'-Chlorobenzyl)-1*H*-indole (10h)[8]

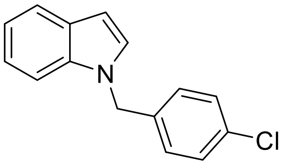

Indoline (0.5 mmol, 59.6 mg) and 4-chlorobenzyl alcohol (1 mmol, 142.6 mg) in TFE (0.5 mL) for 18 h, then the title compound **10h** (88.1 mg) can be obtained under oxidation conditions in 73% yield as yellow oil.

^{1}H NMR (400 MHz, CDCl$_3$) δ 7.68 (d, *J* = 7.6 Hz, 1H), 7.27 (d, *J* = 8.5 Hz, 2H), 7.25 – 7.13 (m, 3H), 7.12 (d, *J* = 3.1 Hz, 1H), 7.04 (d, *J* = 8.5 Hz, 2H), 6.65 – 6.50 (m, 1H), 5.29 (s, 2H).

^{13}C NMR (101 MHz, CDCl$_3$) δ 136.3, 136.2, 133.5, 129.1, 128.9, 128.2, 128.2, 122.0, 121.2, 119.8, 109.7, 102.1, 49.6.

GC-MS m/z (%): 243(M$^+$, 10), 241 (M$^+$, 35), 127 (30), 125 (100), 89 (20), 63 (5).

1-(4'-(Trifluoromethyl)benzyl)-1*H*-indole (10j)[8]

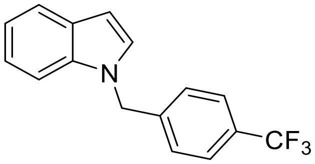

Indoline (0.5 mmol, 59.6 mg) and 4-(trifluoromethyl)benzyl alcohol (1.5 mmol, 264.2 mg) in TFE (0.5 mL) for 30 h, then the title compound **10j** (42.4 mg) can be obtained under oxidation conditions in 31% yield as colorless oil.

^{1}H NMR (400 MHz, CDCl$_3$) δ 7.70 (d, *J* = 7.5 Hz, 1H), 7.56 (d, *J* = 8.1 Hz, 2H), 7.26 – 7.12 (m, 6H), 6.61 (d, *J* = 3.0 Hz, 1H), 5.39 (s, 2H).

^{13}C NMR (101 MHz, CDCl$_3$) δ 141.8 (q, *J* = 1.0 Hz), 136.3, 130.1 (q, *J* = 32.5 Hz), 128.9, 128.3, 127.0, 125.9 (q, *J* = 3.7 Hz), 124.1 (q, *J* = 273.2 Hz), 122.1, 121.3, 120.0, 109.6, 102.4, 49.8.

^{19}F NMR (376 MHz, CDCl$_3$) δ -62.6.

GC-MS m/z (%): 275 (M^+, 100), 204 (5), 159 (90), 130 (25), 109 (25), 89 (20). 63 (10).

1-(Thiophen-2’-ylmethyl)-1*H*-indole (10l)[8]

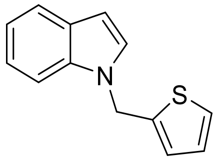

Indoline (0.5 mmol, 59.6 mg) and 2-thiophenemethanol (1 mmol, 114.2 mg) in TFE (1 mL) for 18 h, then the title compound **10l** (70.5 mg) can be obtained under oxidation conditions in 66% yield as colorless oil.

^{1}H NMR (400 MHz, CDCl$_3$) δ 7.68 (d, *J* = 7.9 Hz, 1H), 7.42 (d, *J* = 8.2 Hz, 1H), 7.30 – 7.21 (m, 2H), 7.20 – 7.11 (m, 2H), 7.00 – 6.89 (m, 2H), 6.62 – 6.52 (m, 1H), 5.47 (s, 2H).

^{13}C NMR (101 MHz, CDCl$_3$) δ 140.2, 136.1, 128.9, 127.7, 127.0, 126.0, 125.4, 121.9, 121.2, 119.8, 109.6, 102.2, 45.2.

GC-MS m/z (%): 213 (M^+, 40), 97 (100), 63 (5), 53 (5).

1-Ethyl-1*H*-indole (10n)[15]

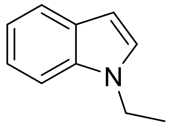

Indoline (0.5 mmol, 59.6 mg) and ethanol (0.5 mL) in TFE (1 mL) for 48 h, then the title compound **10n** (39.5 mg) can be obtained under oxidation conditions in 55% yield as colorless oil.

^{1}H NMR (400 MHz, CDCl$_3$) δ 7.66 (d, *J* = 7.9 Hz, 1H), 7.38 (d, *J* = 8.2 Hz, 1H), 7.26 – 7.20 (m, 1H), 7.17 – 7.09 (m, 2H), 6.52 (d, *J* = 3.0 Hz, 1H), 4.20 (q, *J* = 7.3 Hz, 2H), 1.49 (t, *J* = 7.3 Hz, 3H).

^{13}C NMR (101 MHz, CDCl$_3$) δ 135.8, 128.8, 127.1, 121.5, 121.1, 119.3, 109.4, 101.1, 41.1, 15.6.

GC-MS m/z (%): 145 (M^+, 65), 130 (100), 103 (13), 89 (15), 77 (10), 63 (5).

1-Propyl-1*H*-indole (10o) [15]

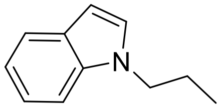

Indoline (0.5 mmol, 59.6 mg) and 1-propanol (0.5 mL) in TFE (1 mL) for 48 h, then the title compound **10o** (52.5 mg) can be obtained under oxidation conditions in 66% yield as colorless oil.

^{1}H NMR (400 MHz, $CDCl_3$) δ 7.66 (d, *J* = 7.9 Hz, 1H), 7.37 (d, *J* = 8.2 Hz, 1H), 7.23 (t, *J* = 7.6 Hz, 1H), 7.18 – 7.07 (m, 2H), 6.51 (d, *J* = 3.0 Hz, 1H), 4.11 (t, *J* = 7.1 Hz, 2H), 1.95 – 1.83 (m, 2H), 0.96 (t, *J* = 7.4 Hz, 3H).

^{13}C NMR (101 MHz, $CDCl_3$) δ 136.1, 128.7, 128.0, 121.4, 121.1, 119.3, 109.5, 100.9, 48.2, 23.7, 11.7.

GC-MS m/z (%): 159 (M^+, 35), 130 (100), 103 (5), 77 (10), 63 (5).

1-Butyl-1*H*-indole (10p) [15]

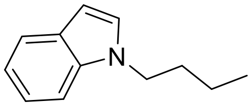

Indoline (0.5 mmol, 59.6 mg) and 1-butanol (0.5 mL) in TFE (1 mL) for 48 h, then the title compound **10p** (62.5 mg) can be obtained under oxidation conditions in 72% yield as colorless oil.

^{1}H NMR (400 MHz, $CDCl_3$) δ 7.67 (d, *J* = 7.9 Hz, 1H), 7.38 (d, *J* = 8.2 Hz, 1H), 7.26 – 7.21 (m, 1H), 7.17 – 7.09 (m, 2H), 6.52 (d, *J* = 3.0 Hz, 1H), 4.15 (t, *J* = 7.1 Hz, 2H), 1.90 – 1.81 (m, 2H), 1.43 – 1.31 (m, 2H), 0.97 (t, *J* = 7.4 Hz, 3H).

^{13}C NMR (101 MHz, $CDCl_3$) δ 136.1, 128.7, 127.9, 121.4, 121.1, 119.3, 109.5, 100.9, 46.2, 32.5, 20.3, 13.8.

GC-MS m/z (%): 173 (M^+, 30), 130 (100), 117 (5), 103 (10), 77 (10).

1-(3'-Phenylprop-1'-yl)-1*H*-indole (10q)[15]

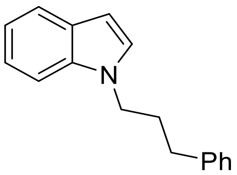

Indoline (0.5 mmol, 59.6 mg) and 3-phenylpropanol (1 mmol, 136.2 mg) in TFE (0.5 mL) for 18 h, then the title compound **10q** (58.8 mg) can be obtained under oxidation conditions in 50% yield as colorless oil.

^{1}H NMR (400 MHz, $CDCl_3$) δ 7.65 (d, *J* = 7.9 Hz, 1H), 7.36 – 7.29 (m, 3H), 7.25 – 7.16 (m, 4H), 7.16 – 7.07 (m, 2H), 6.52 (d, *J* = 3.0 Hz, 1H), 4.15 (t, *J* = 7.1 Hz, 2H), 2.66 (t, *J* = 7.6 Hz, 2H), 2.27 – 2.15 (m, 2H).

^{13}C NMR (101 MHz, $CDCl_3$) δ 141.1, 136.1, 128.8, 128.6, 128.5, 127.9, 126.2, 121.5, 121.1, 119.4, 109.5, 101.2, 45.8, 33.1, 31.6.

GC-MS m/z (%): 235 (M^+, 60), 130 (100), 117 (15), 103 (15), 91 (15), 77 (10), 65 (5).

1-Cinnamyl-1*H*-indole (10r)[16]

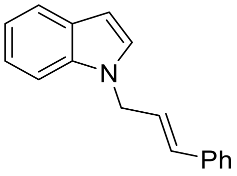

Indoline (0.5 mmol, 59.6 mg) and cinnamyl alcohol (1 mmol, 134.2 mg) in TFE (1 mL) for 18 h, then the title compound **10r** (60.5 mg) can be obtained under oxidation conditions in 52% yield as colorless oil.

^{1}H NMR (400 MHz, CDCl$_3$) δ 7.72 (d, *J* = 7.7 Hz, 1H), 7.43 (d, *J* = 8.2 Hz, 1H), 7.41 – 7.23 (m, 6H), 7.22 – 7.12 (m, 2H), 6.66 – 6.56 (m, 1H), 6.53 (d, *J* = 15.8 Hz, 1H), 6.38 (dt, *J* = 15.8, 5.7 Hz, 1H), 4.92 (d, *J* = 5.7 Hz, 2H).

^{13}C NMR (101 MHz, CDCl$_3$) δ 136.4, 136.3, 132.4, 128.9, 128.7, 128.0, 127.9, 126.6, 125.0, 121.7, 121.1, 119.6, 109.7, 101.7, 48.5.

GC-MS m/z (%): 233 (M^+, 40), 117 (100), 91 (15), 77 (5), 63 (5).

1-(3'-Methylbut-2'-en-1'-yl)-1*H*-indole (10s)[17]

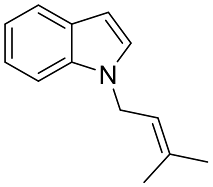

Indoline (0.5 mmol, 59.6 mg) and 3-methyl-2-buten-1-ol (2 mmol, 172.3 mg) in TFE (0.5 mL) for 18 h, then the title compound **10s** (48.9 mg) can be obtained under oxidation conditions in 53% yield as colorless oil.

^{1}H NMR (400 MHz, CDCl$_3$) δ 7.69 (d, *J* = 7.9 Hz, 1H), 7.39 (d, *J* = 8.2 Hz, 1H), 7.26 (t, *J* = 7.6 Hz, 1H), 7.21 – 7.08 (m, 2H), 6.55 (d, *J* = 3.1 Hz, 1H), 5.54 – 5.35 (m, 1H), 4.74 (d, *J* = 6.8 Hz, 2H), 1.88 (s, 3H), 1.82 (s, 3H).

^{13}C NMR (101 MHz, CDCl$_3$) δ 136.3, 136.1, 128.9, 127.5, 121.4, 121.0, 120.1, 119.4, 109.6, 101.0, 44.3, 25.8, 18.1.

GC-MS m/z (%): 185 (M^+, 40), 117 (100), 90 (10), 69 (20).

1,4-Bis((1’*H*-indol-1’-yl)methyl)benzene (10aa)[18]

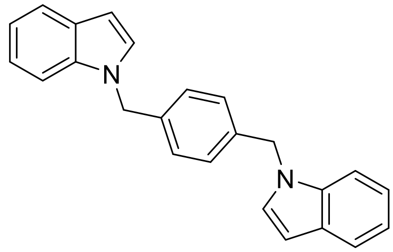

Indoline (1.5 mmol, 178.7 mg) and 1,4-phenylenedimethanol (0.5 mmol, 69.1 mg) in TFE (0.5 mL) for 18 h, then the title compound **10aa** (68.0 mg) can be obtained under oxidation conditions in 40% yield as brown solid.

^{1}H NMR (400 MHz, CDCl$_3$) δ 7.65 (d, *J* = 7.6 Hz, 2H), 7.25 (d, *J* = 7.0 Hz, 2H), 7.19 – 7.09 (m, 6H), 7.03 (s, 4H), 6.55 (dd, *J* = 3.1, 0.6 Hz, 2H), 5.28 (s, 4H).

^{13}C NMR (101 MHz, CDCl$_3$) δ 137.2, 136.4, 128.9, 128.3, 127.3, 121.9, 121.1, 119.7, 109.7, 101.9, 49.8.

GC-MS m/z (%): 336 (M^+, 100), 220 (85), 205 (10), 168 (20), 116 (15), 104 (85), 89 (15), 78 (15), 63 (5).

6) Mechanism studies and characterization data

a) Reaction of *N*-benzylindoline **9a** with radical scavengers.

Table S3 - Reaction of *N*-benzylindoline with different radical scavengers under oxidation conditions[a].

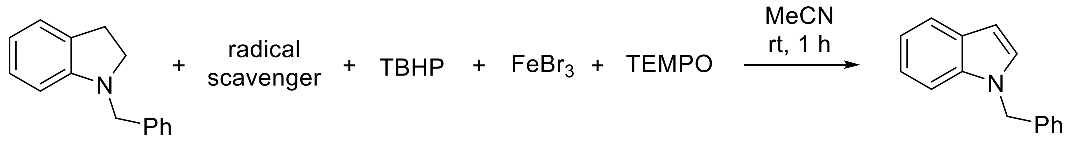

9a **10a**

Entry	Radical scavenger (equiv.)	TBHP (equiv.)	$FeBr_3$ (equiv.)	TEMPO (equiv.)	Yield[b] of **10a** (%)
Under stoichiometric conditions without TBHP as oxidant					
1	Galvinoxyl (2)	-	1	1	80
2	1,1- Diphenylethene (3)	-	1	1	94
Under optimized catalytic conditions in the presence of TBHP as oxidant					
3	BHT (3)	3	0.1	0.5	58

[a]Reaction conditions: **9a** (0.1 mmol, 1 equiv.), radical scavenger (2-3 equiv.), $FeBr_3$ (0.1-1 equiv.), TEMPO (0.5-1 equiv.), TBHP (0-3 equiv.) and acetonitrile (3 mL) under argon at room temperature for 1 h. [b]Yields were determined by ^{1}H NMR using CH_2Br_2 as the internal standard.

b) Reaction of 1-(pent-4'-en-1'-yl)indoline **9-1** with $FeBr_3$ and TEMPO

Table S4 - Reaction of 1-(pent-4'-en-1'-yl)indoline[a].

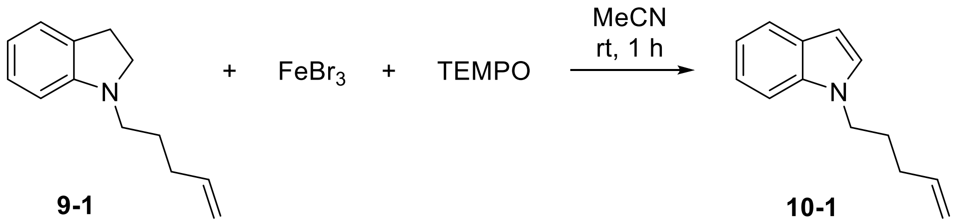

9-1 **10-1**

Entry	Conditions	Yield[b] of **10-1**(%)
1	$FeBr_3$ (1 equiv.), TEMPO (1 equiv.)	99
2	$FeBr_3$ (1 equiv.), TEMPO (5 equiv.)	90 (82)

[a] Reaction conditions: **9-1** (0.2 mmol, 1 equiv.), $FeBr_3$ (0.2 mmol, 1 equiv.), TEMPO (1-5 equiv.) and acetonitrile (3 mL) under argon at room temperature for 1 h. [b] Yields were determined by ^{1}H NMR using CH_2Br_2 as the internal standard, and isolated yield was given in parentheses.

c) Radical clock experiment

Scheme S1 - Reaction of *N*-cyclopropylindoline 9-2.

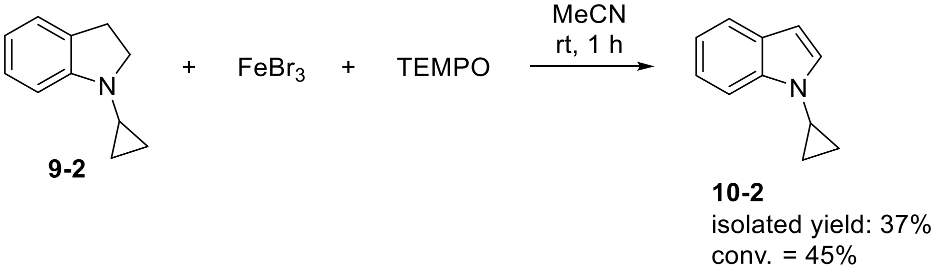

Reaction conditons: **9-2** (0.26 mmol, 1 equiv.), $FeBr_3$ (0.26 mmol, 1 equiv.), TEMPO (0.26 mmol, 1 equiv.) and acetonitrile (3 mL) under argon at room temperature for 1 h.

d) Use of Bobbit's salt as the oxidant species of indoline **9a**

Table S5 - Reaction of *N*-benzylindoline 9a with Bobbit's salt

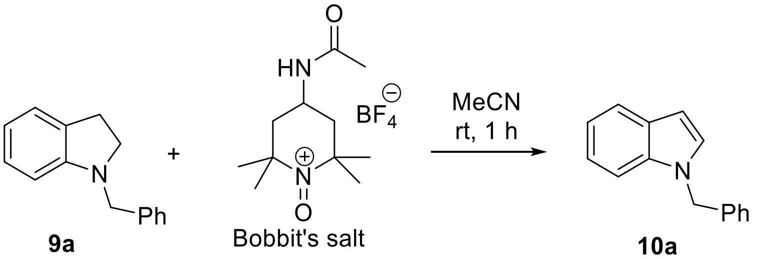

Entry	Bobbit's salt	$FeBr_3$	TBHP	**9a** (%)[b]	**10a** (%)[b]
1	100 mol%	-	-	0	80
2	20 mol%	-	3 equiv.	68	32
3	20 mol%	100 mol%	-	80	19
4	50 mol%	10 mol%	3 equiv.	0	80

[a] Reaction conditons: **9a** (0.2 mmol, 1 equiv.), acetonitrile (3 mL) under argon at room temperature for 1 h. [b] NMR yield using CH_2Br_2 as internal standard.

1-(Pent-4'-en-1'-yl)-1*H*-indole (10-1)[19]

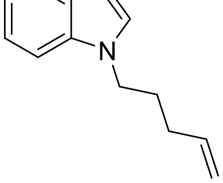

^{1}H NMR (400 MHz, CDCl$_3$) δ 7.69 (d, *J* = 7.9 Hz, 1H), 7.39 (d, *J* = 8.1 Hz, 1H), 7.29 – 7.22 (m, 1H), 7.19 – 7.10 (m, 2H), 6.55 (d, *J* = 2.7 Hz, 1H), 5.95 – 5.74 (m, 1H), 5.16 – 5.02 (m, 2H), 4.17 (t, *J* = 7.0 Hz, 2H), 2.17 – 2.08 (m, 2H), 2.04 – 1.94 (m, 2H).

^{13}C NMR (101 MHz, CDCl$_3$) δ 136.7, 135.3, 128.0, 127.2, 120.7, 120.3, 118.6, 115.0, 108.7, 100.4, 45.0, 30.2, 28.6.

GC-MS m/z (%): 185 (M$^+$, 40), 170 (15), 130 (100), 117 (10), 103 (10), 77 (15), 63 (5).

1-Cyclopropyl-1*H*-indole (10-2)[20]

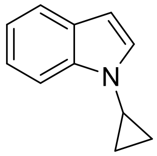

^{1}H NMR (400 MHz, CDCl$_3$) δ 7.65 – 7.54 (m, 2H), 7.26 – 7.21 (m, 1H), 7.16 – 7.10 (m, 2H), 6.45 (d, *J* = 2.8 Hz, 1H), 3.40 – 3.31 (m, 1H), 1.10 – 0.99 (m, 4H).

^{13}C NMR (101 MHz, CDCl$_3$) δ 137.6, 128.9, 128.2, 121.6, 121.0, 119.9, 110.5, 101.2, 27.1, 6.3.

GC-MS m/z (%): 156 (M$^+$, 100), 142 (5), 130 (30), 102 (10), 89 (20), 77 (10). 63 (5).

7) X-ray analysis of compound 9aa

Crystals were obtained by recrystallization from dichloromethane and heptane at room temperature.

A suitable crystal for X-ray diffraction single crystal experiment (colorless plate, dimensions = 0.700 x 0.470 x 0.070 mm) was selected and mounted on the goniometer head of a D8 Venture (Bruker-AXS) diffractometer equipped with a CMOS-PHOTON70 detector, using Mo-Kα radiation (λ = 0.71073 Å, multilayer monochromator) at T = 150(2) K. Crystal structure has been described in monoclinic symmetry and $P\ 2_1/c$ (I.T.#14) centric space group. Cell parameters have been refined as follows: a = 9.8645(19), b = 10.243(2), c = 9.0325(16) Å, β = 98.409(8) °, V = 902.9(3) $Å^3$. Number of formula unit Z is equal to 2 and calculated density d and absorption coefficient μ values are 1.252 $g.cm^{-3}$ and 0.073 mm^{-1} respectively. Crystal structure was solved by dual-space algorithm using SHELXT program, and then refined with full-matrix least-squares methods based on F^2 (SHELXL). All non-Hydrogen atoms were refined with anisotropic atomic displacement parameters. H atoms were finally included in their calculated positions and treated as riding on their parent atom with constrained thermal parameters. A final refinement on F^2 with 2053 unique intensities and 118 parameters converged at ωR_{F2} = 0.1490 (RF = 0.0660) for 1713 observed reflections with $I > 2\sigma(I)$.

Structure data for compound **9aa**

Empirical formula	$C_{24}H_{24}N_2$
Formula weight	340.45 g/mol
Temperature	150(2) K
Radiation type	Mo-Kα
Wavelength	0.71073 Å
Crystal system, space group	monoclinic, $P\ 2_1/c$ (I.T.#14)
Unit cell dimensions	a = 9.8645(19) Å
	b = 10.243(2) Å
	c = 9.0325(16) Å
	β = 98.409(8) °
Volume	902.9(3) $Å^3$
Z, Calculated density	2, 1.252 $g.cm^{-3}$
Absorption coefficient	0.073 mm^{-1}
F(000)	364
Crystal size	0.700 x 0.470 x 0.070 mm
Crystal color	colourless
Crystal description	plate
Diffractometer	D8 Venture (Bruker-AXS)
Detector	CMOS-PHOTON70
θ range for data collection	2.087 to 27.434°

(sinθ/λ)max (Å-1)	0.648
h_{min}, h_{max}	-12, 12
k_{min}, k_{max}	-13, 11
lmin, l_{max}	-10, 11
Reflections collected / unique	9406 / 2053 [R(int)^a = 0.0677]
Reflections [I>2σ]	1713
Completeness to θ_{max}	0.996
Absorption correction type	multi-scan
Max. and min. transmission	0.995, 0.655
Refinement method	Full-matrix least-squares on F^2
H-atom treatment	H-atom parameters constrained
Data / restraints / parameters	2053 / 0 / 118
^{b}S (Goodness-of-fit)	1.093
Shelxl weighting scheme parameters	a = 0.0747, b = 0.3635
Final R indices [I>2σ]	$R1^c$ = 0.0660, $wR2^d$ = 0.1490
Final R indices [all data]	$R1^c$ = 0.0784, $wR2^d$ = 0.1580
Δρmax, Δρmin	0.394, -0.302 e^-. $Å^{-3}$

$^aRint = \Sigma\ |F_o^2\ - < F_o^2>| / \Sigma [F_o^2]$

$^bS = \{\Sigma\ [w(F_o^2\ -\ F_c^2)^2] / (n - p)\}^{1/2}$

$^cR1 = \Sigma\ ||F_o| - |F_c|| / \Sigma\ |F_o|$

$^dwR2 = \{\Sigma\ [w(F_o^2 -\ F_c^2)^2] / \Sigma\ [w(F_o^2)^2]\}^{1/2}$

$w = 1 / [\sigma(F_o^2) + aP^2 + bP]$ where $P = [2F_c^2 + MAX(F_o^2, 0)] /3$

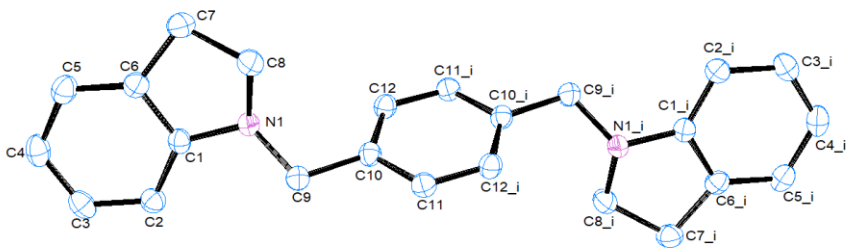

Figure S1 – Molecular structure of the compound **9aa**. Thermal ellipsoids correspond to 50% probability. Only one molecule is depicted and hydrogens were omitted for clarity.

8) NMR spectra

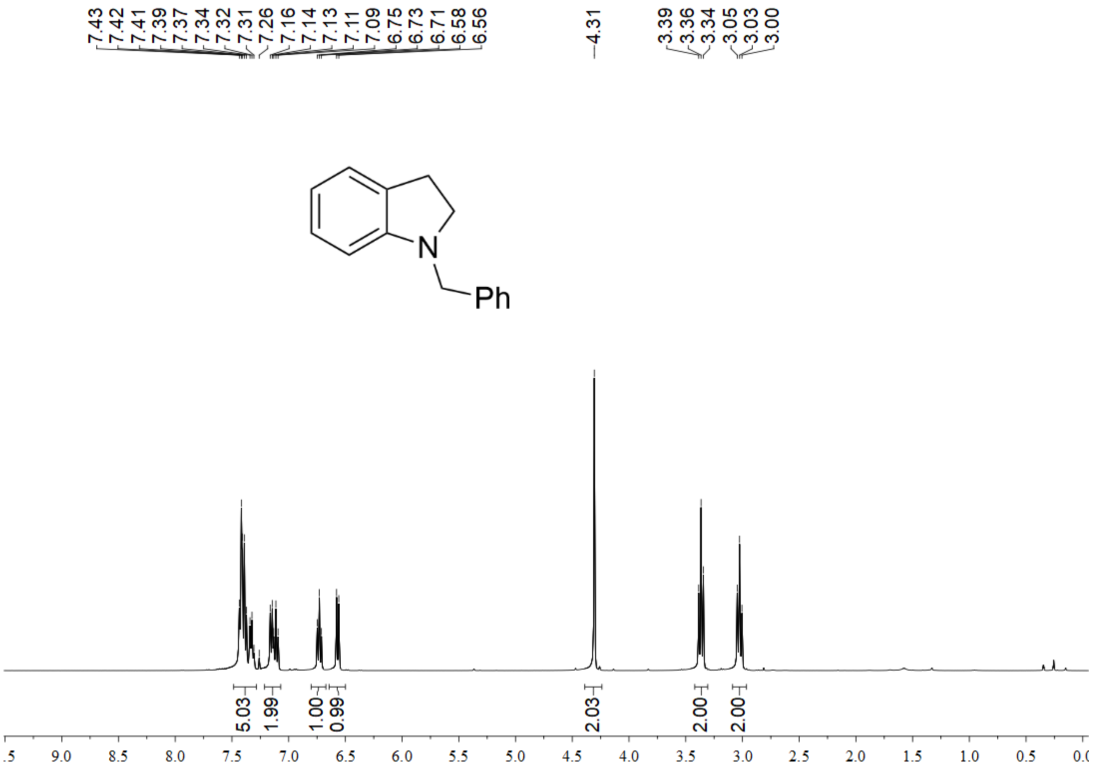

Figure S2 - ^{1}H NMR (400 MHz, $CDCl_3$) of **9a**

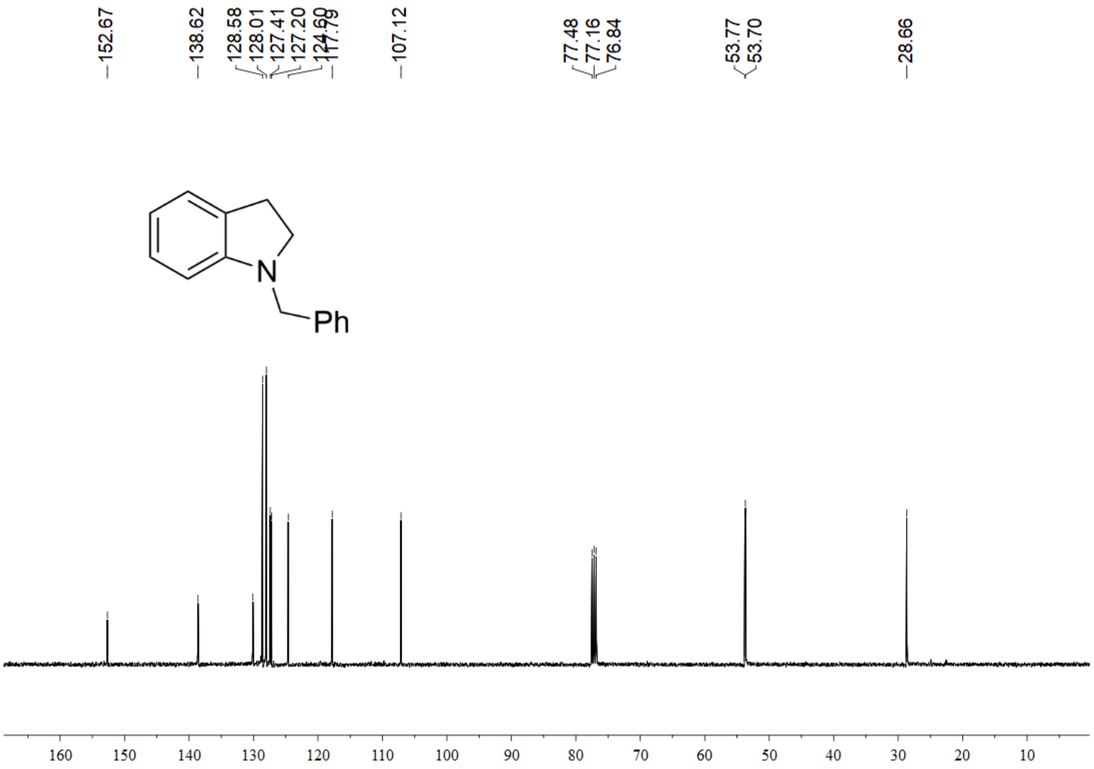

Figure S3 - $^{13}C\{^{1}H\}$ NMR (101 MHz, $CDCl_3$) of **9a**

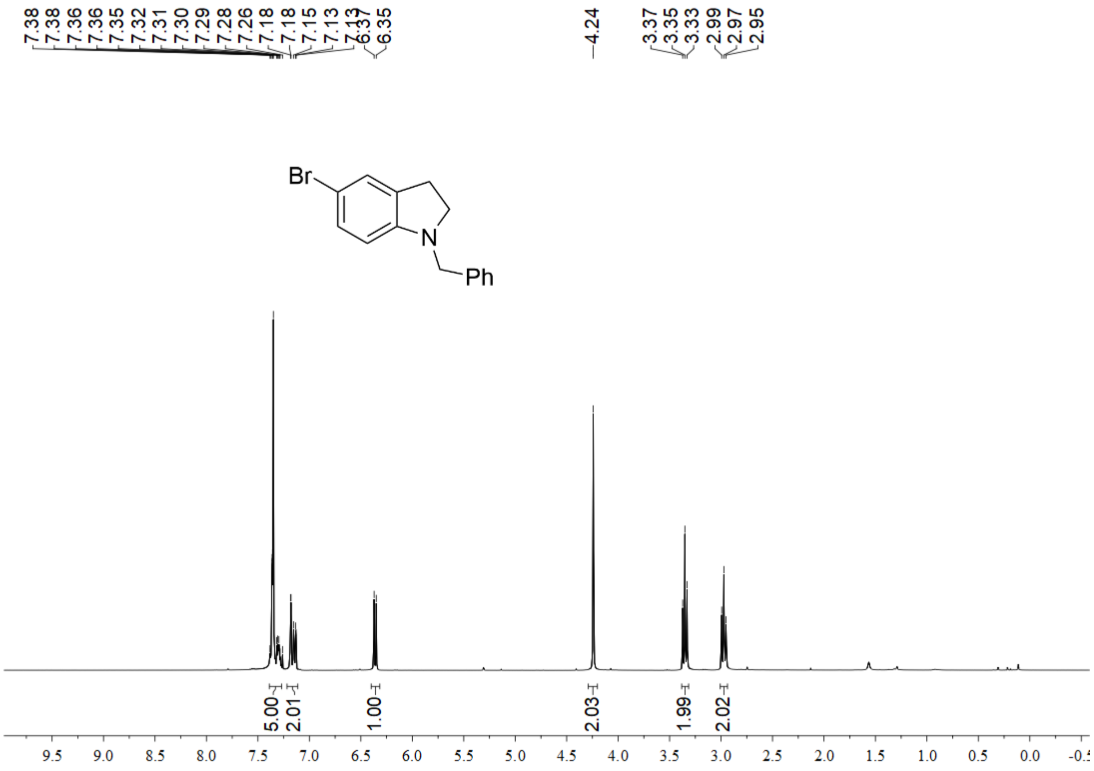

Figure S4 - ^{1}H NMR (400 MHz, $CDCl_3$) of **9b**

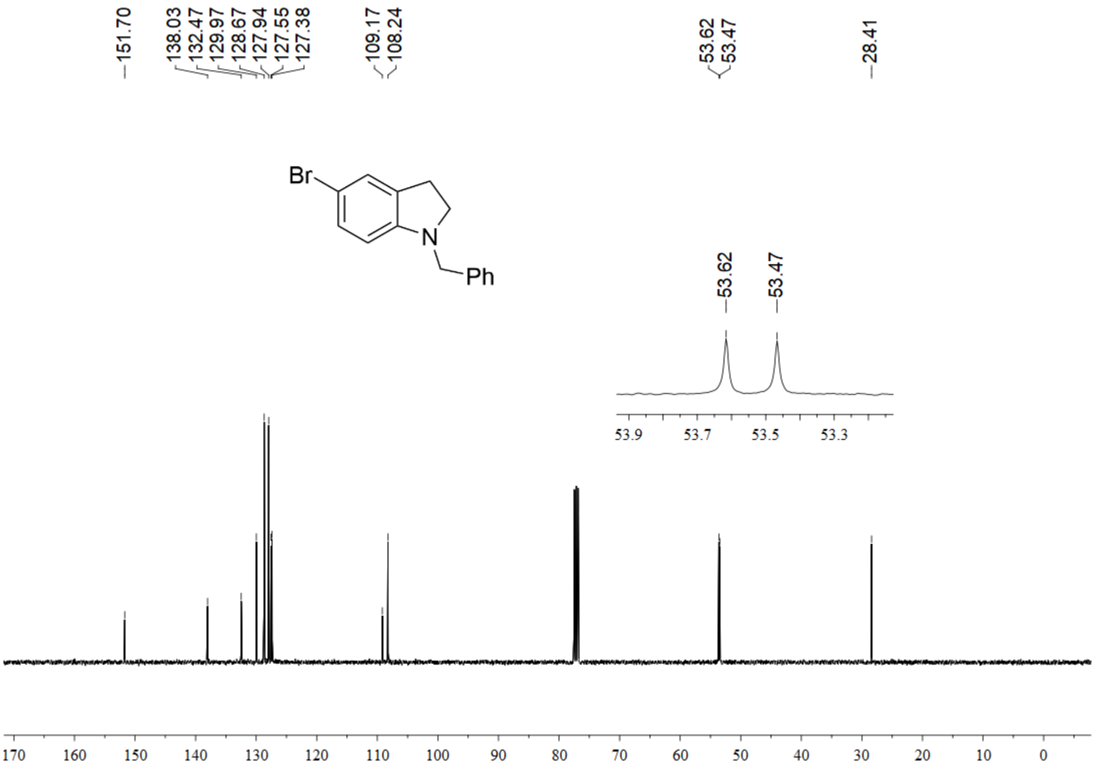

Figure S5 - $^{13}C\{^1H\}$ NMR (101 MHz, $CDCl_3$) of **9b**

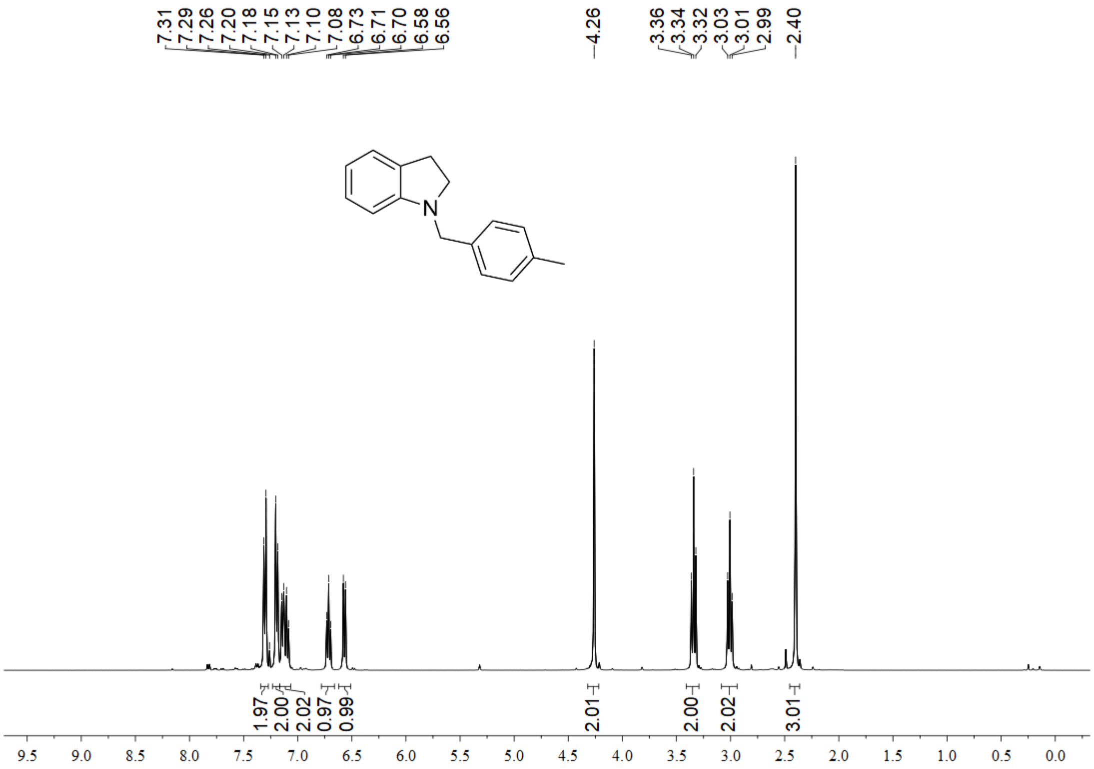

Figure S6 - ^{1}H NMR (400 MHz, $CDCl_3$) of **9c**

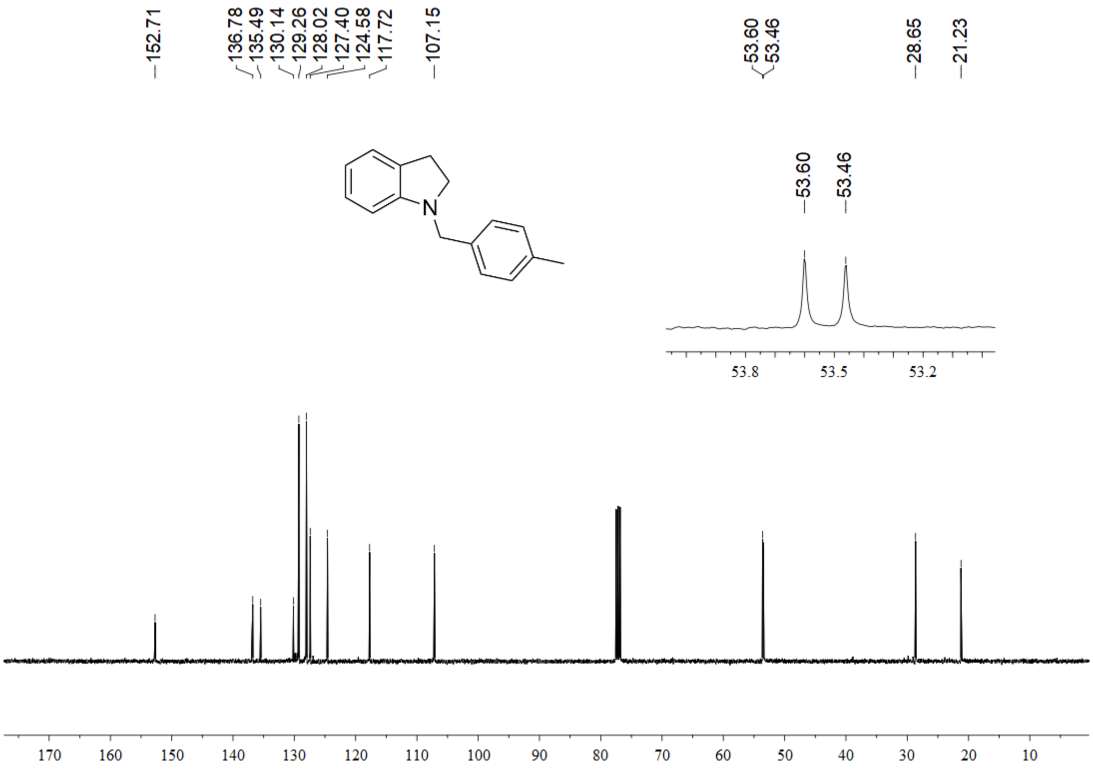

Figure S7 - $^{13}C\{^{1}H\}$ NMR (101 MHz, $CDCl_3$) of **9c**

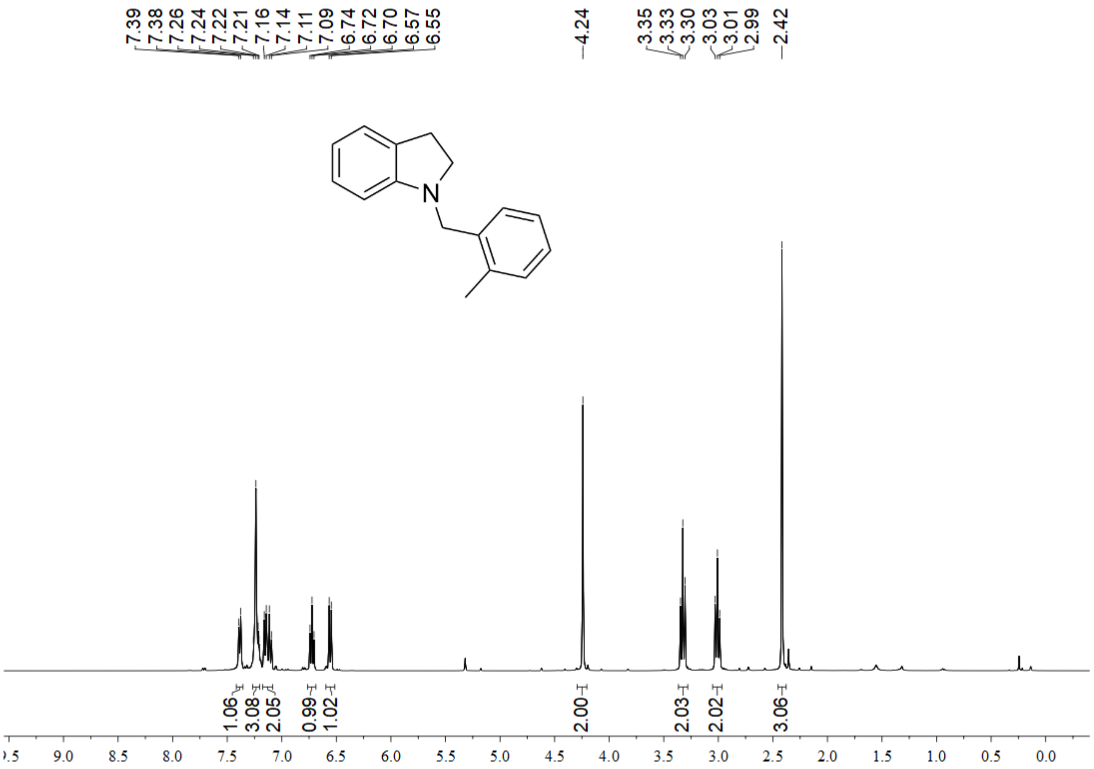

Figure S8 - ^{1}H NMR (400 MHz, $CDCl_3$) of **9d**

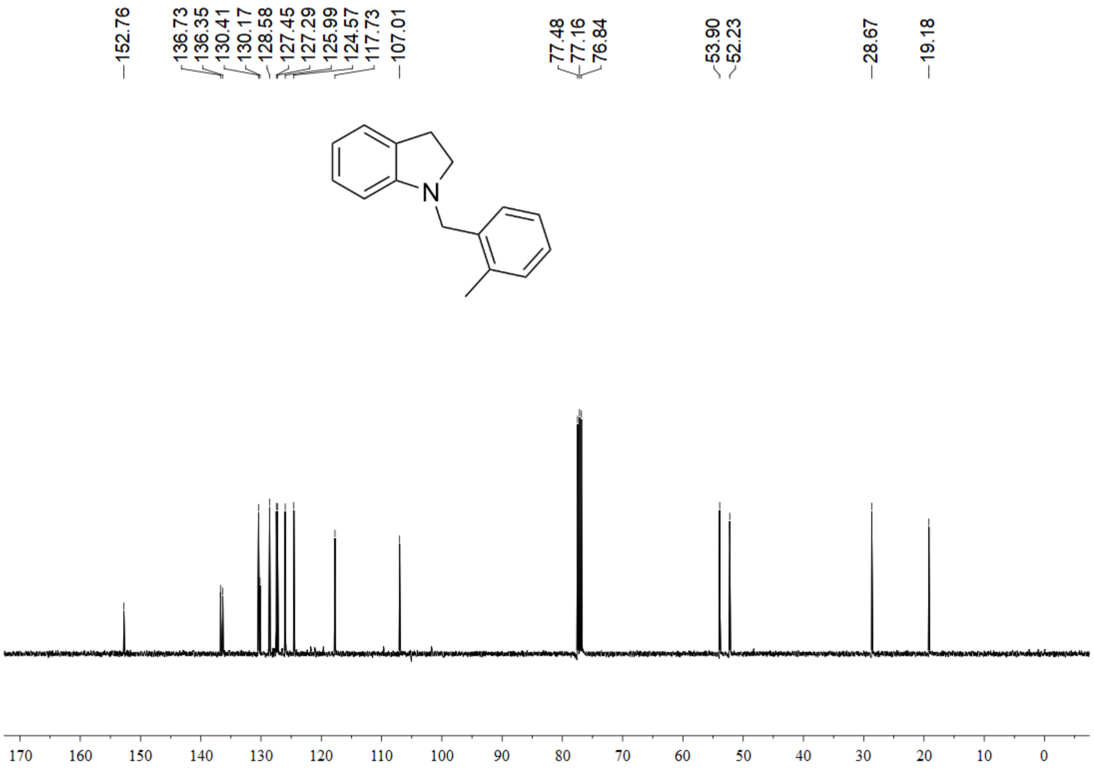

Figure S9 - ^{13}C{^{1}H} NMR (101 MHz, $CDCl_3$) of **9d**

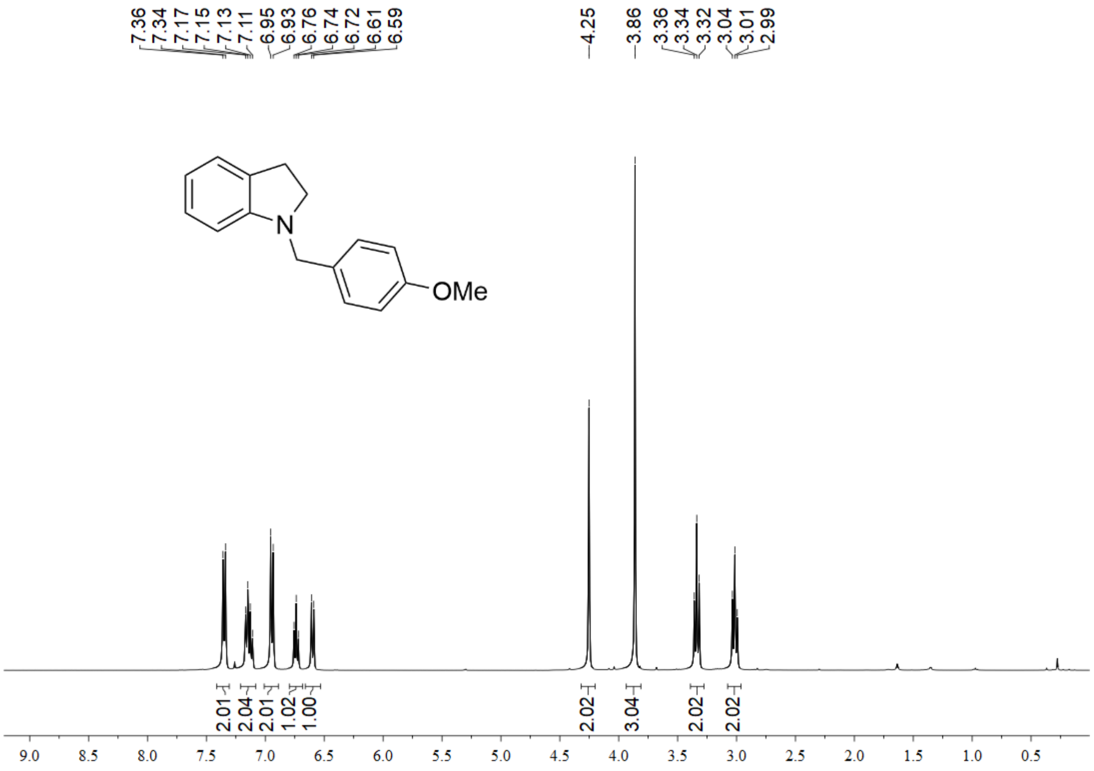

Figure S10 - ^{1}H NMR (400 MHz, $CDCl_3$) of **9e**

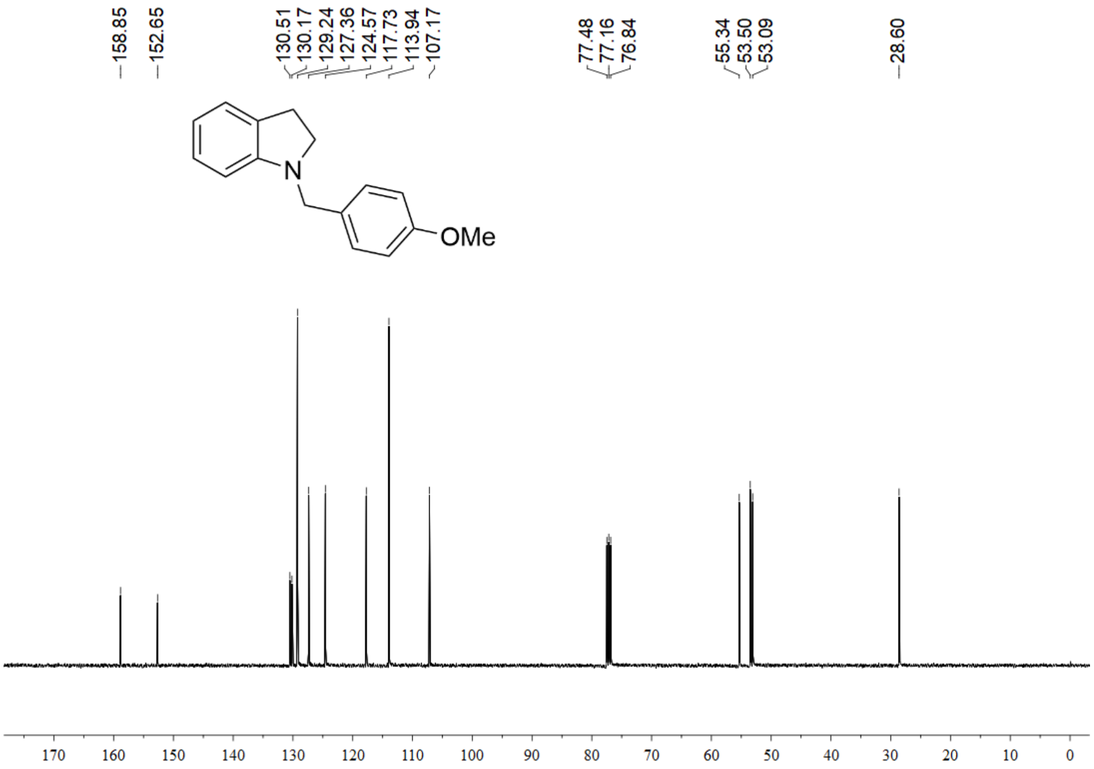

Figure S11 - $^{13}C\{^{1}H\}$ NMR (101 MHz, $CDCl_3$) of **9e**

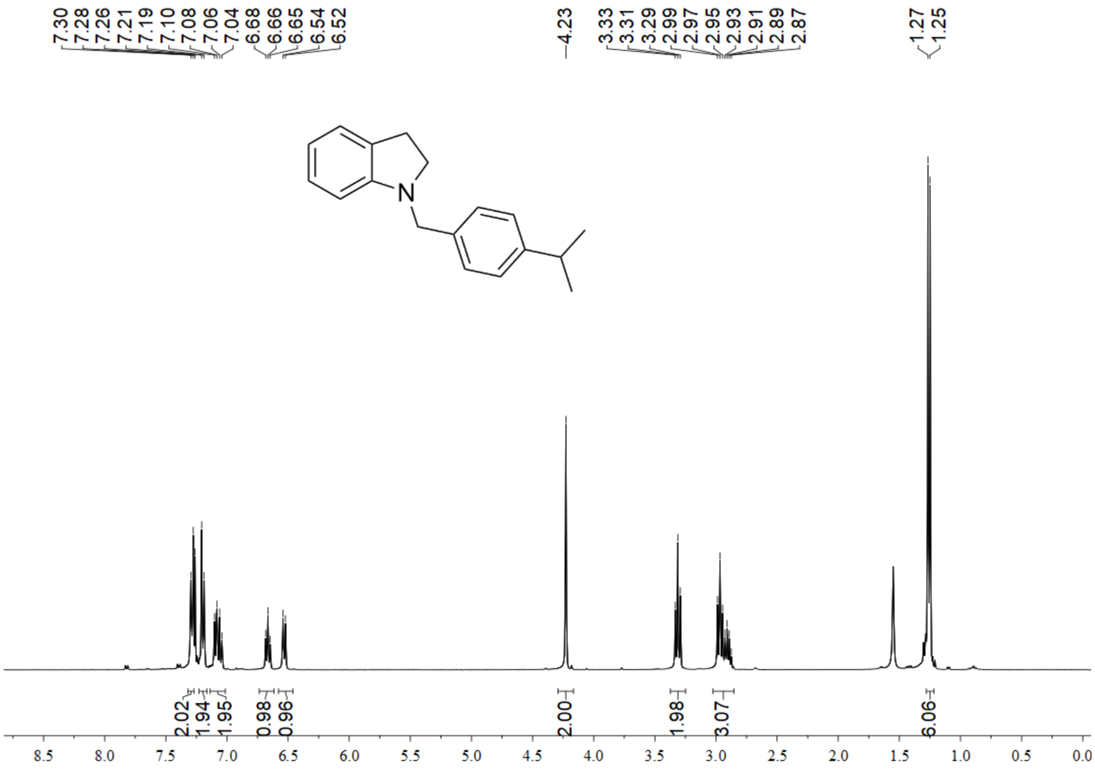

Figure S12 - ^{1}H NMR (400 MHz, $CDCl_3$) of **9f**

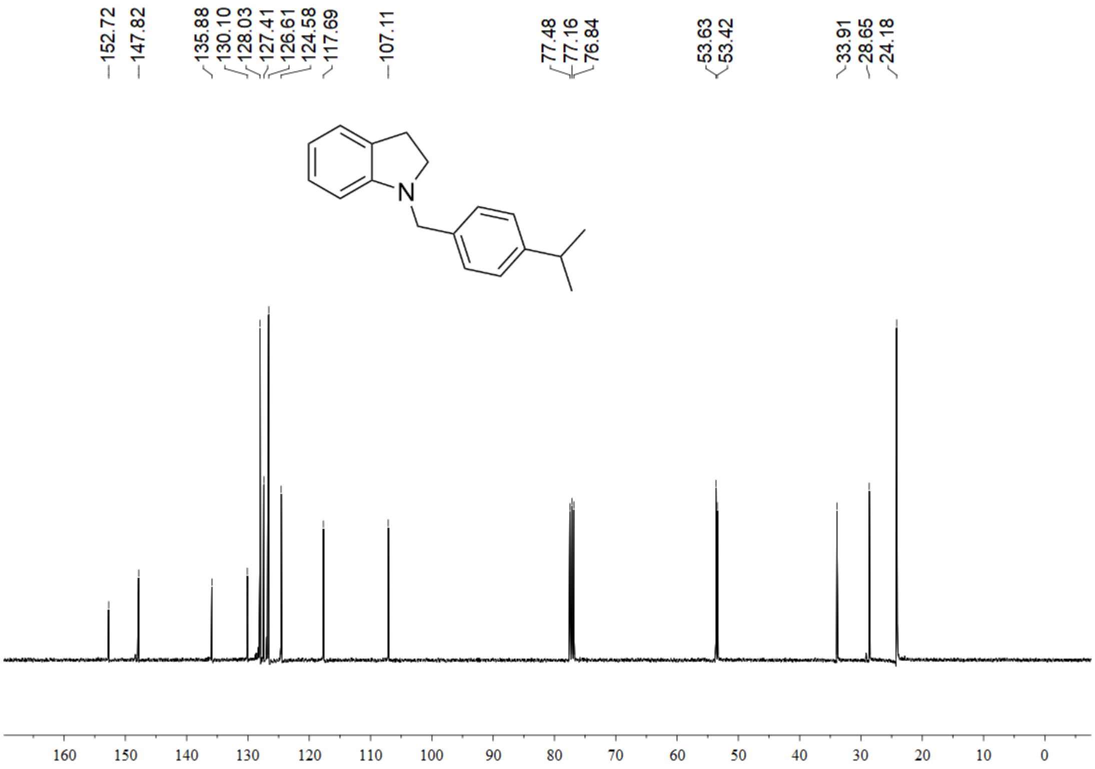

Figure S13 - $^{13}C\{^{1}H\}$ NMR (101 MHz, $CDCl_3$) of **9f**

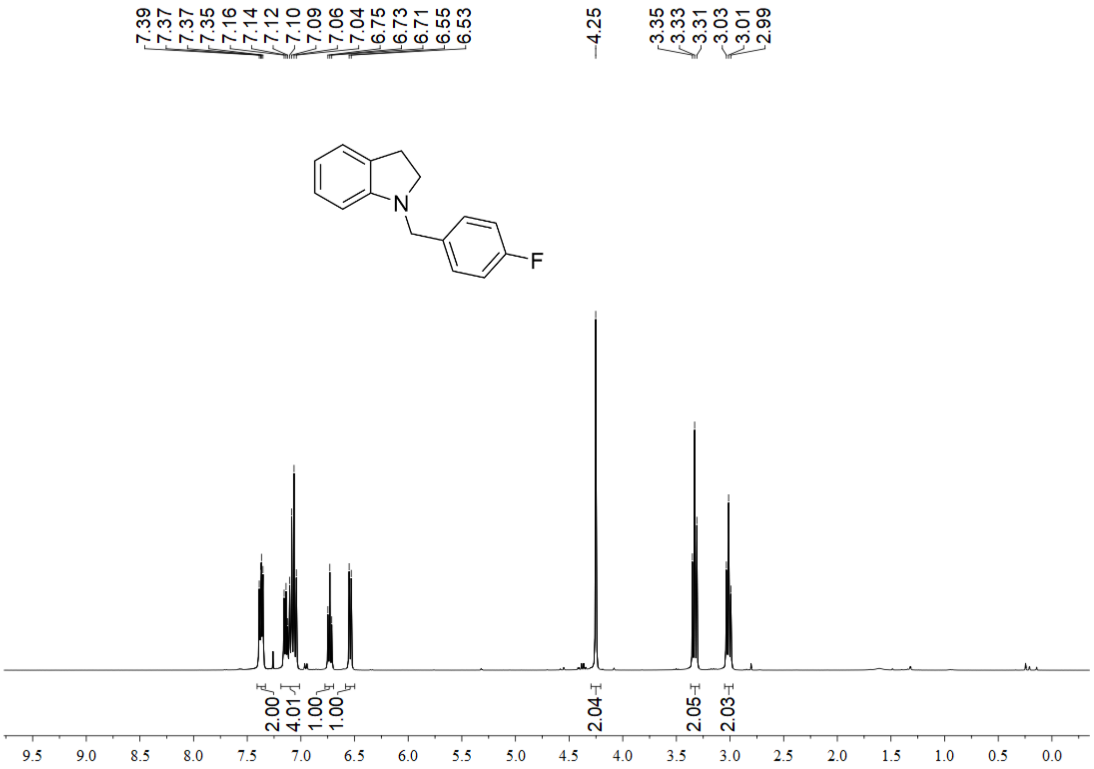

Figure S14 - ^{1}H NMR (400 MHz, $CDCl_3$) of **9g**

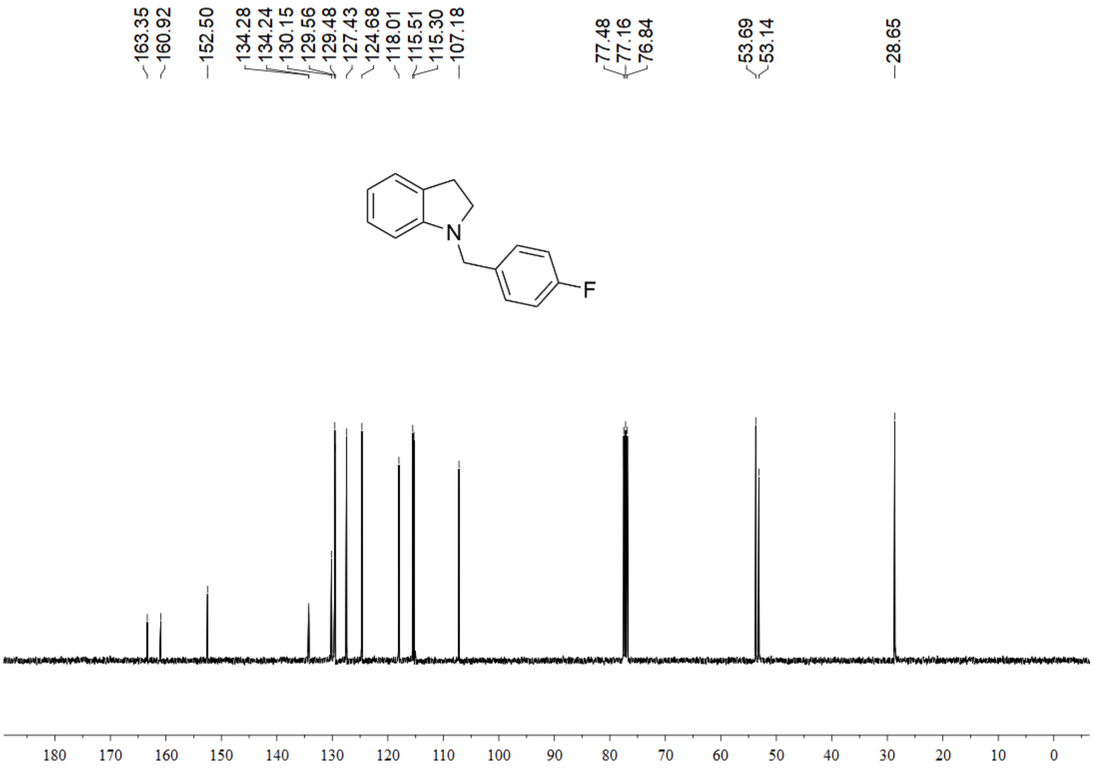

Figure S15 - $^{13}C\{^{1}H\}$ NMR (101 MHz, $CDCl_3$) of **9g**

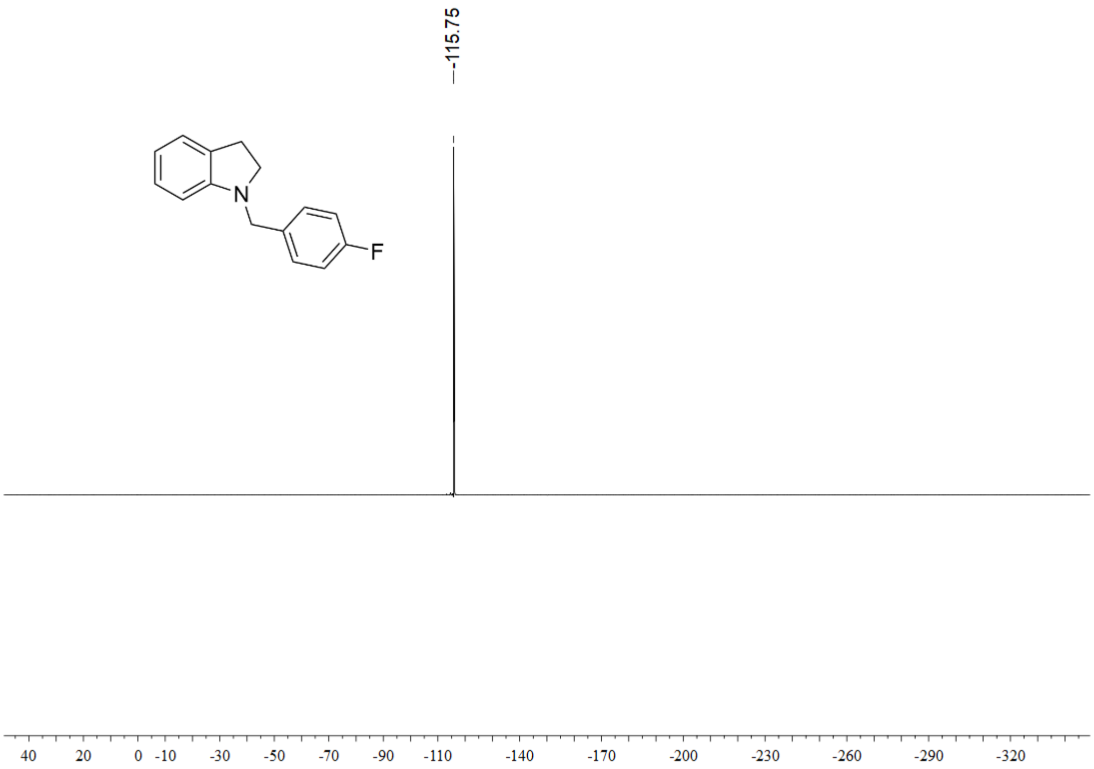

Figure S16 - ^{19}F NMR (376 MHz, $CDCl_3$) of **9g**

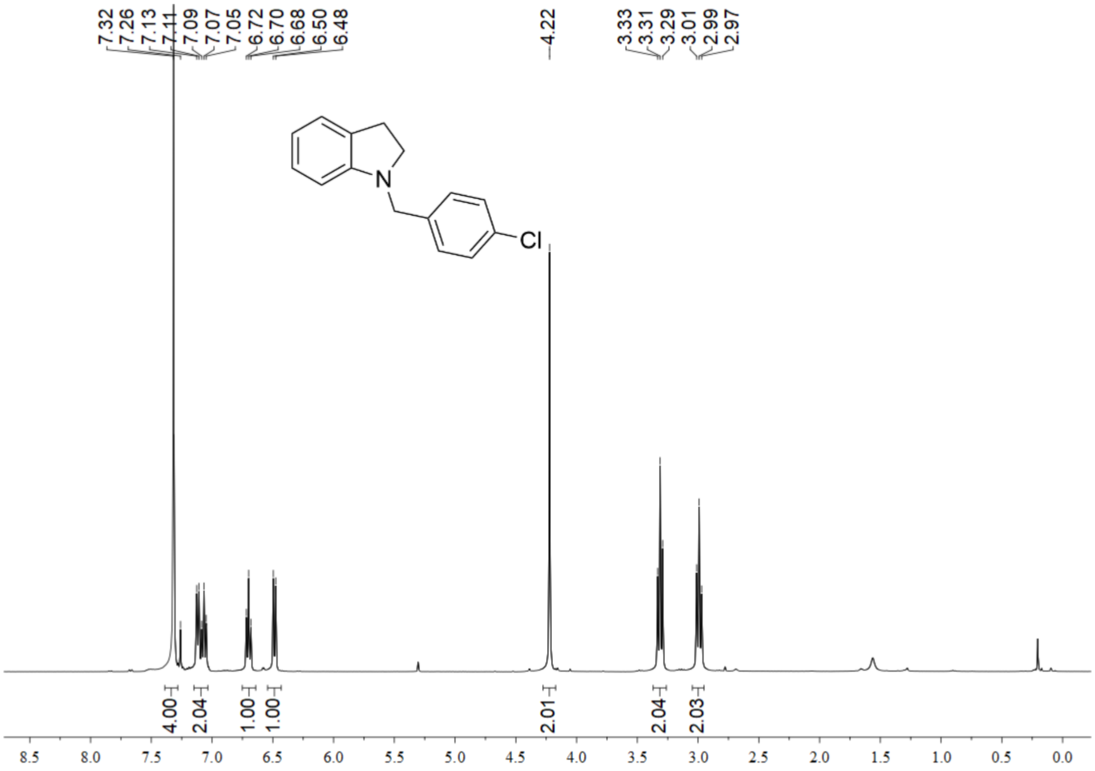

Figure S17 - ^{1}H NMR (400 MHz, $CDCl_3$) of **9h**

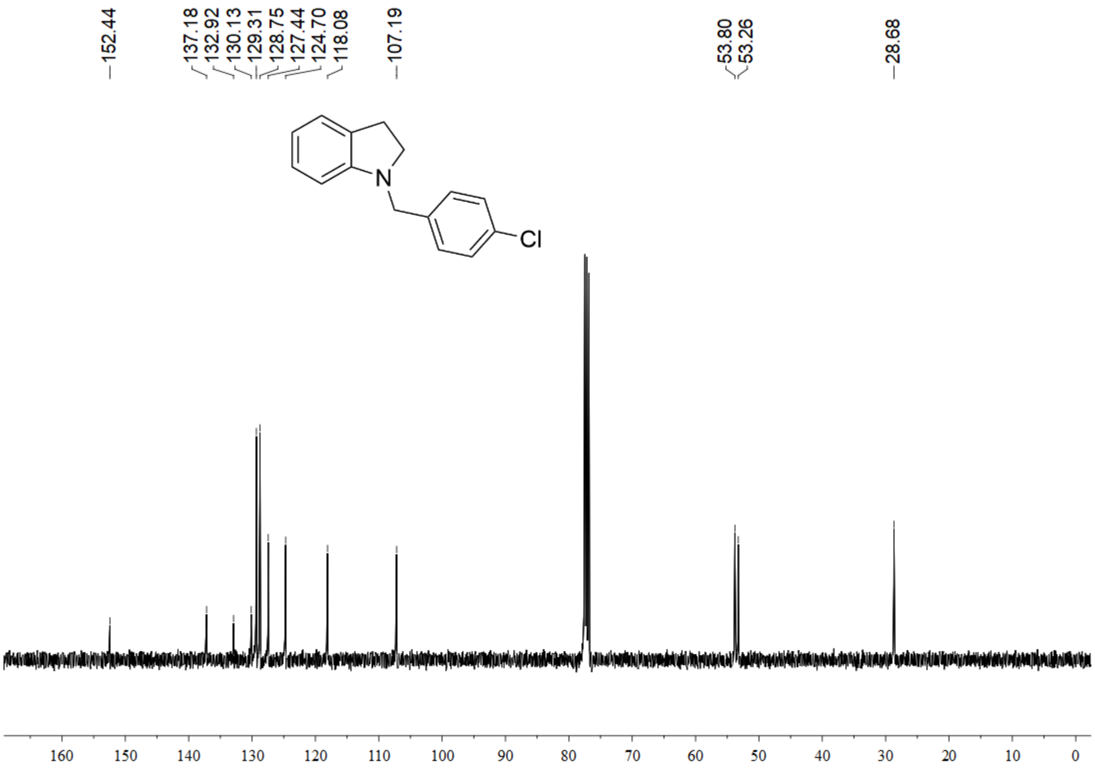

Figure S18 - $^{13}C\{^{1}H\}$ NMR (101 MHz, $CDCl_3$) of **9h**

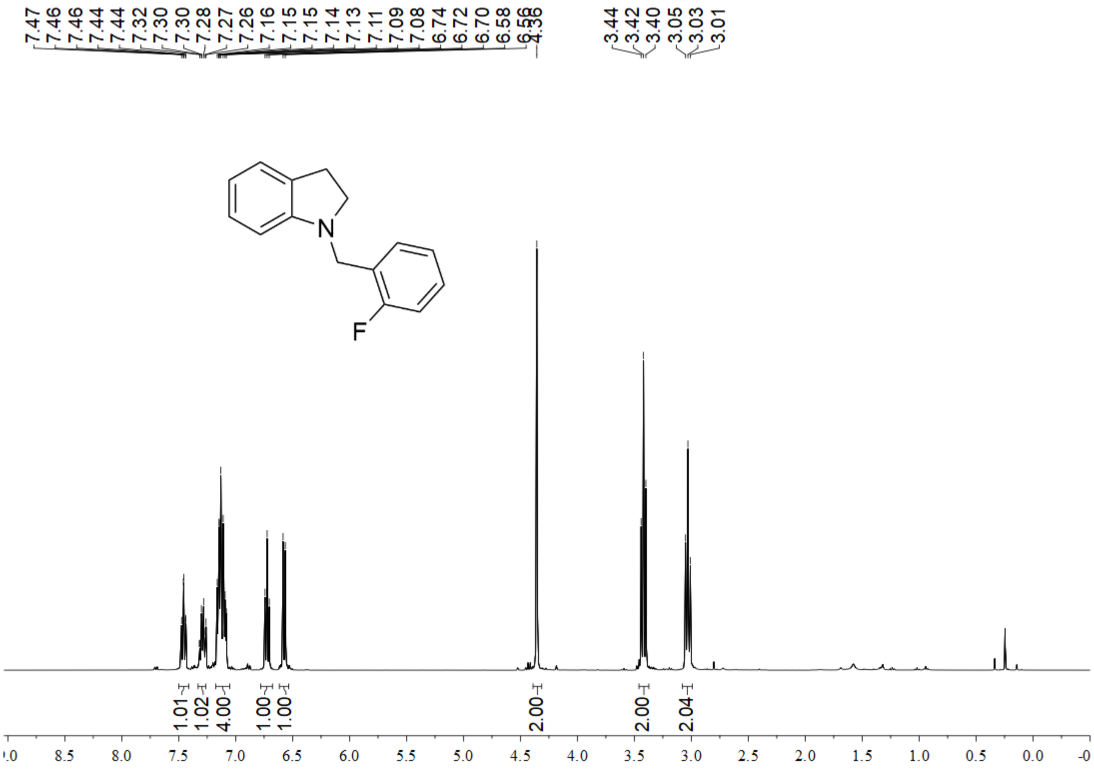

Figure S19 - ^{1}H NMR (400 MHz, $CDCl_3$) of **9i**

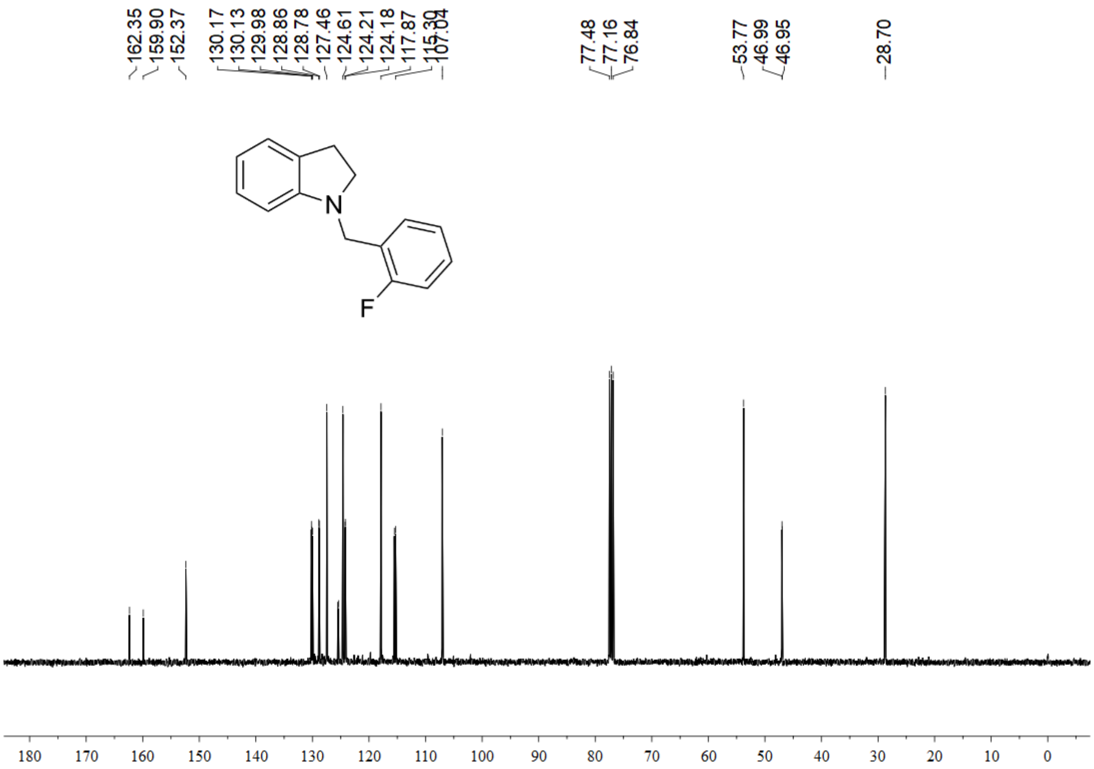

Figure S20 - $^{13}C\{^{1}H\}$ NMR (101 MHz, $CDCl_3$) of **9i**

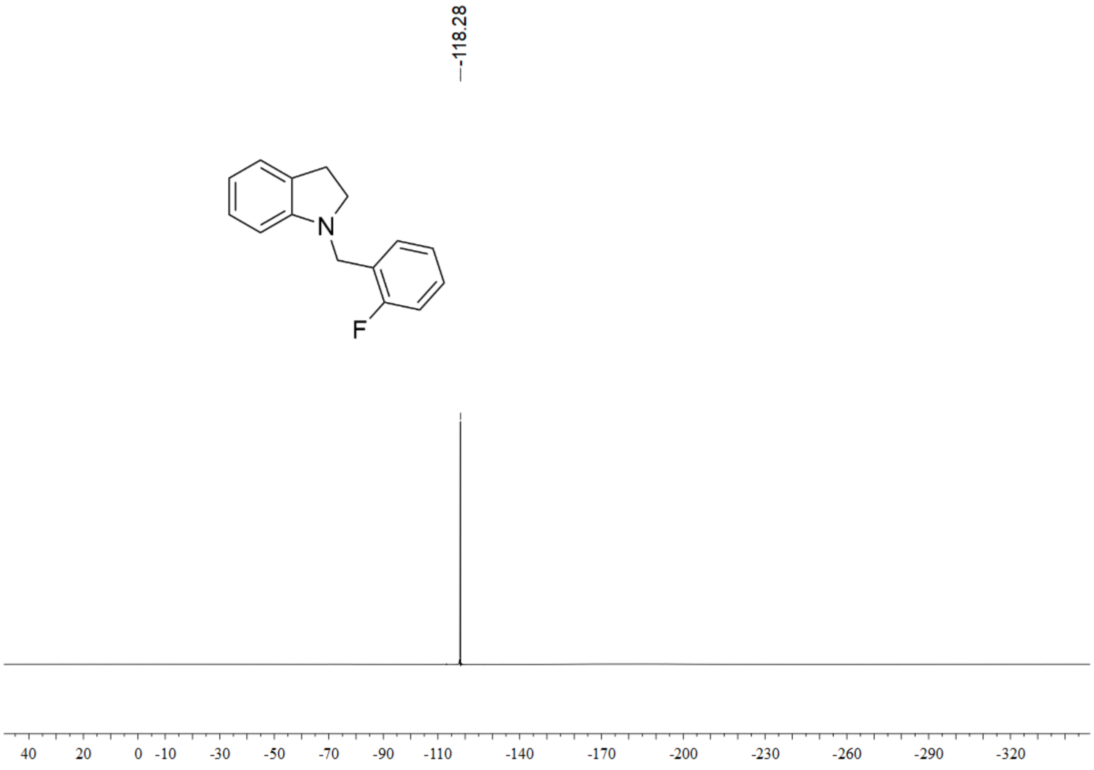

Figure S21 - ^{19}F NMR (376 MHz, $CDCl_3$) of **9i**

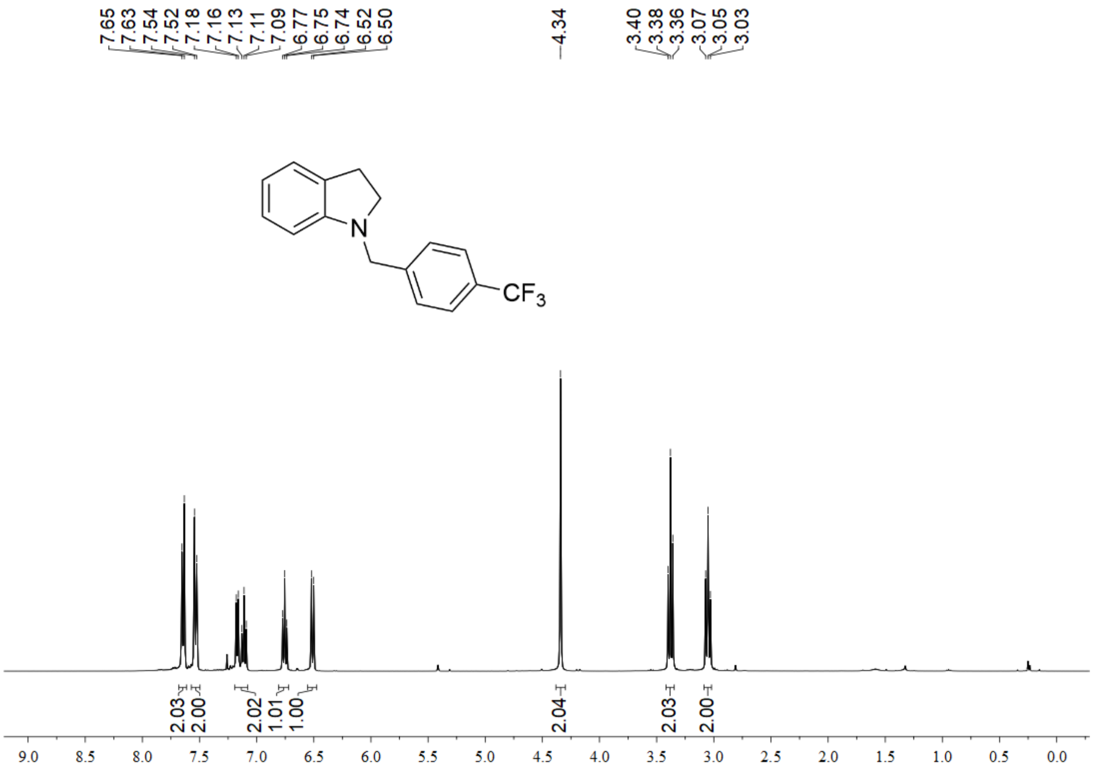

Figure S22 - ^{1}H NMR (400 MHz, $CDCl_3$) of **9j**

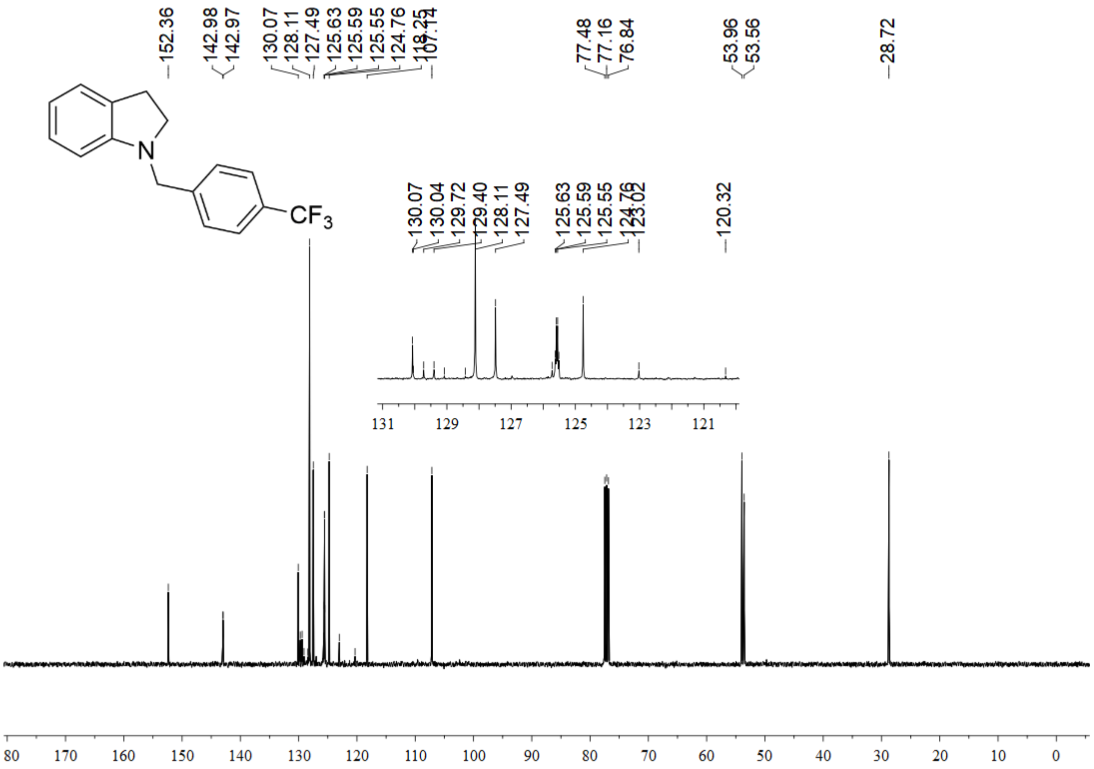

Figure S23 - ^{13}C{^{1}H} NMR (101 MHz, $CDCl_3$) of **9j**

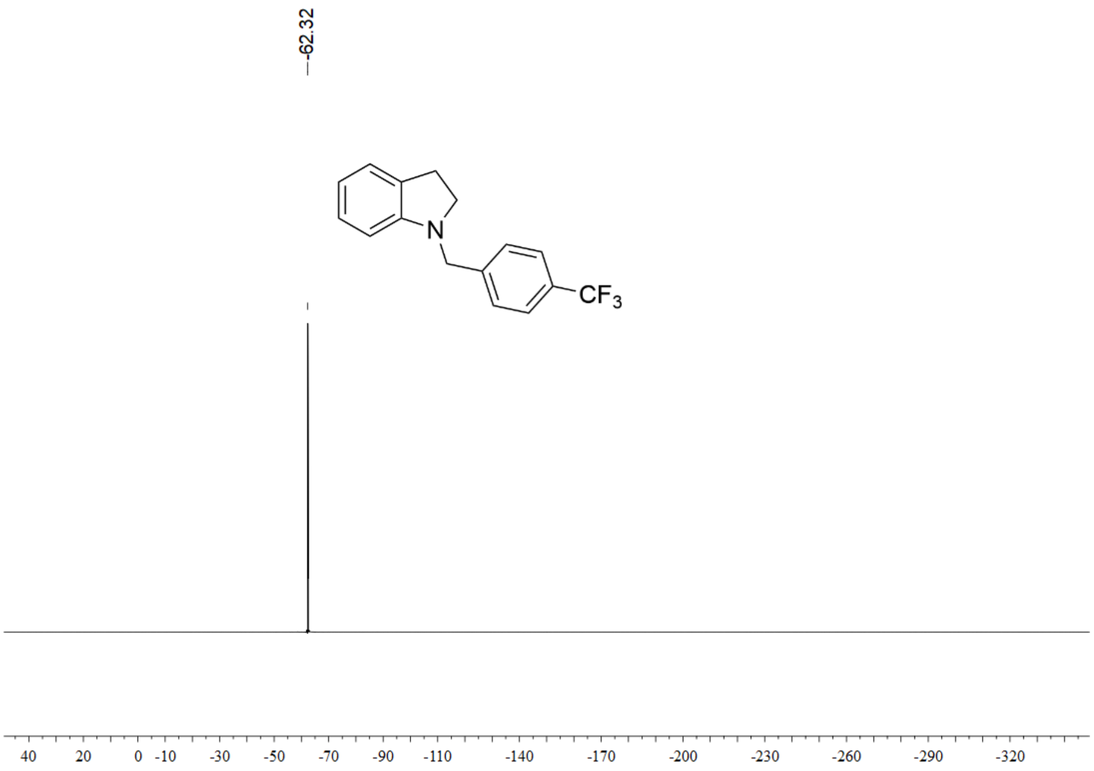

Figure S24 - ^{19}F NMR (376 MHz, $CDCl_3$) of **9j**

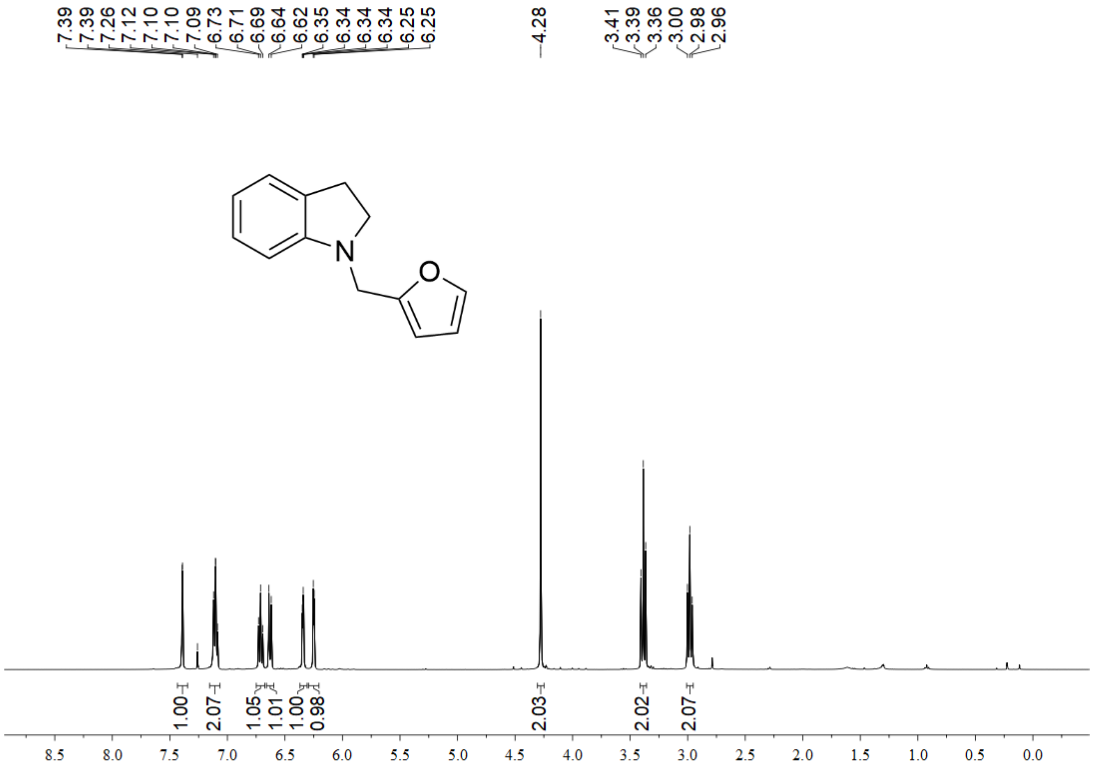

Figure S25 - ^{1}H NMR (400 MHz, $CDCl_3$) of **9k**

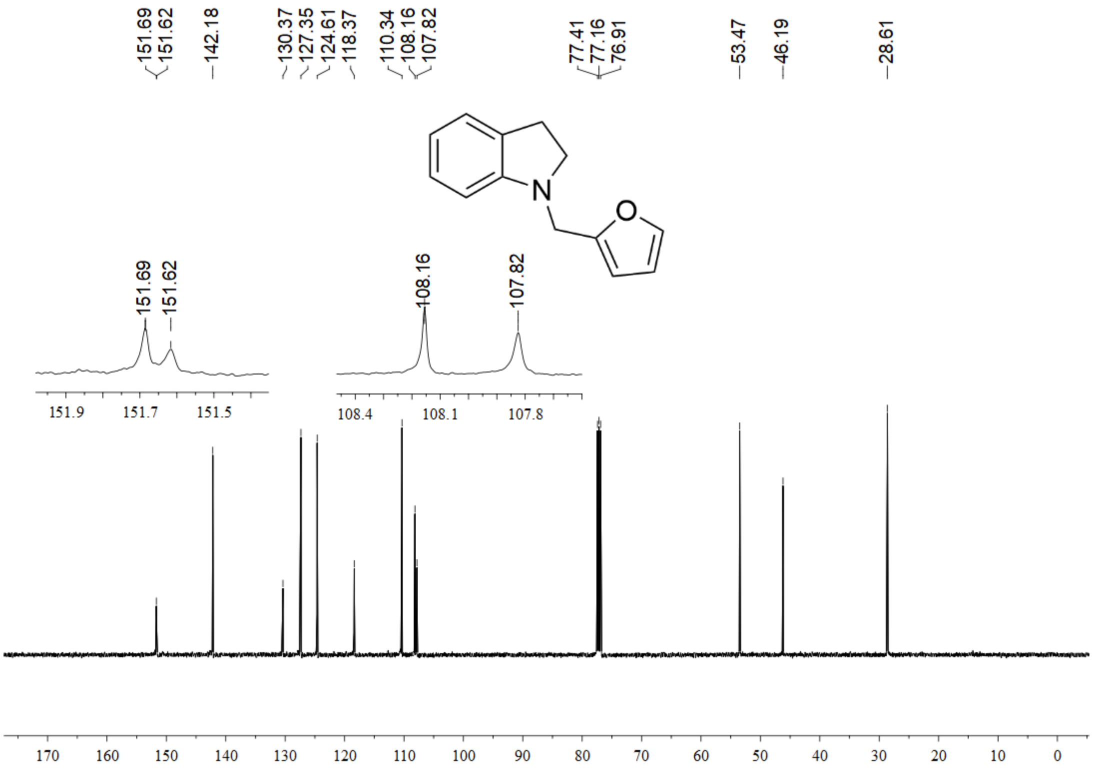

Figure S26 - $^{13}C\{^{1}H\}$ NMR (126 MHz, $CDCl_3$) of **9k**

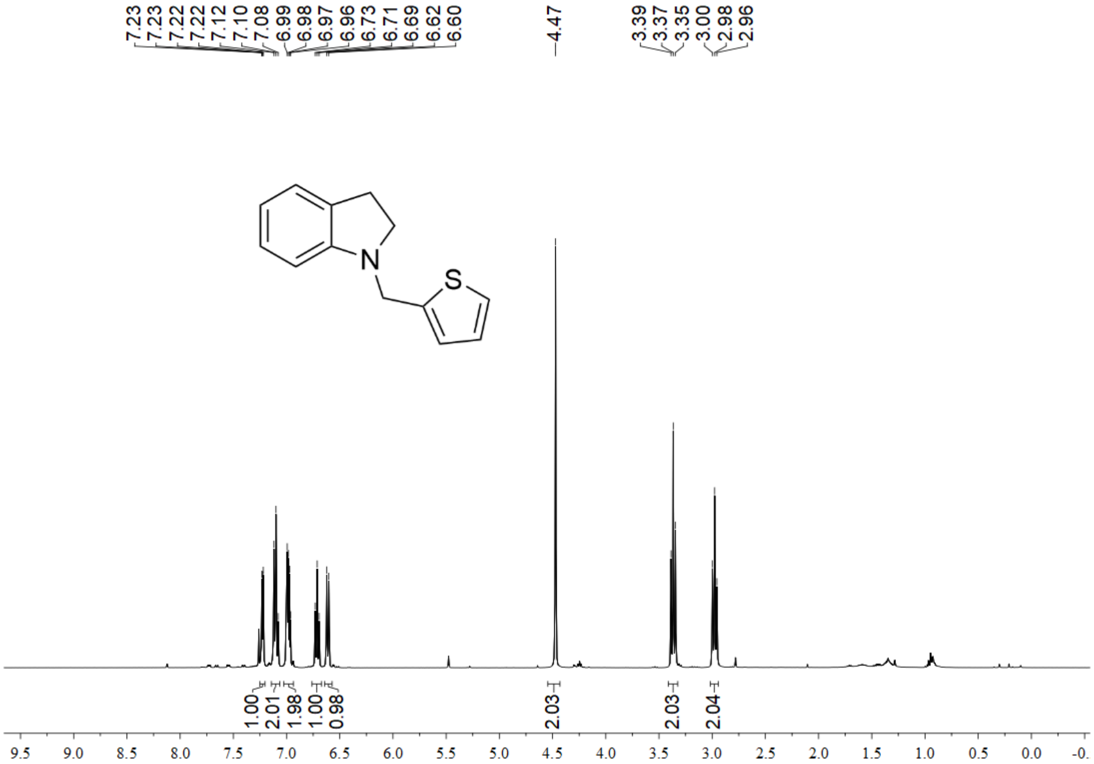

Figure S27 - ^{1}H NMR (400 MHz, $CDCl_3$) of **9l**

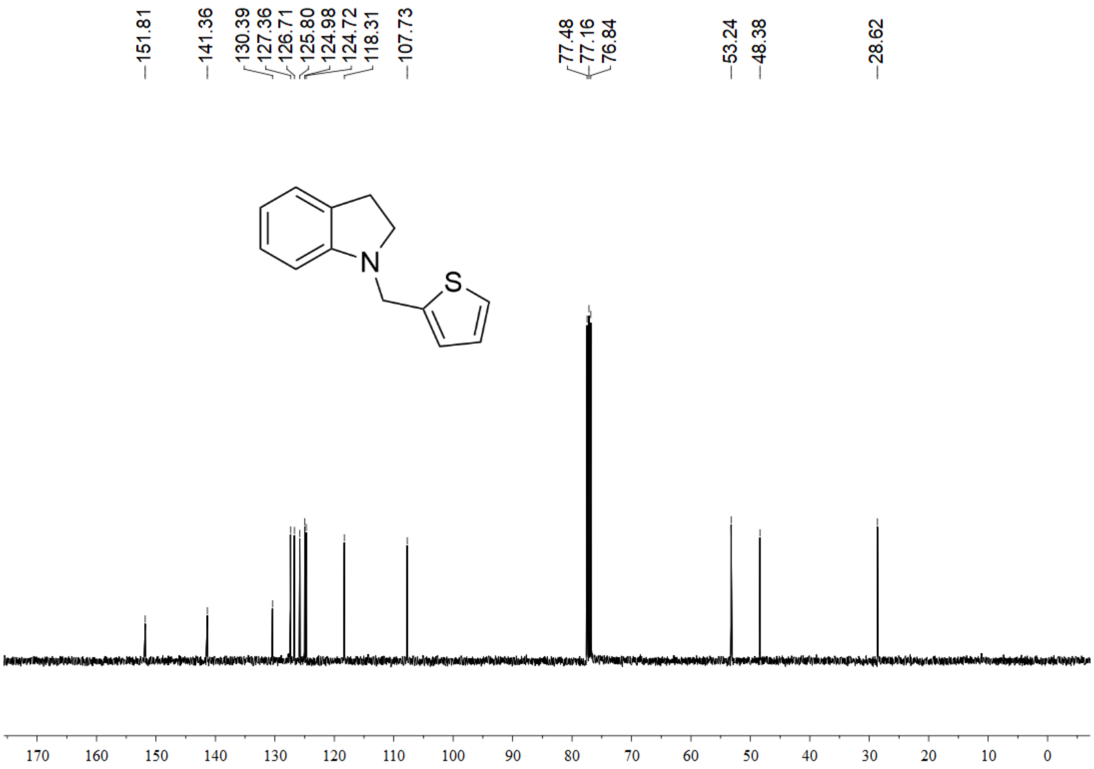

Figure S28 - $^{13}C\{^{1}H\}$ NMR (126 MHz, $CDCl_3$) of **9l**

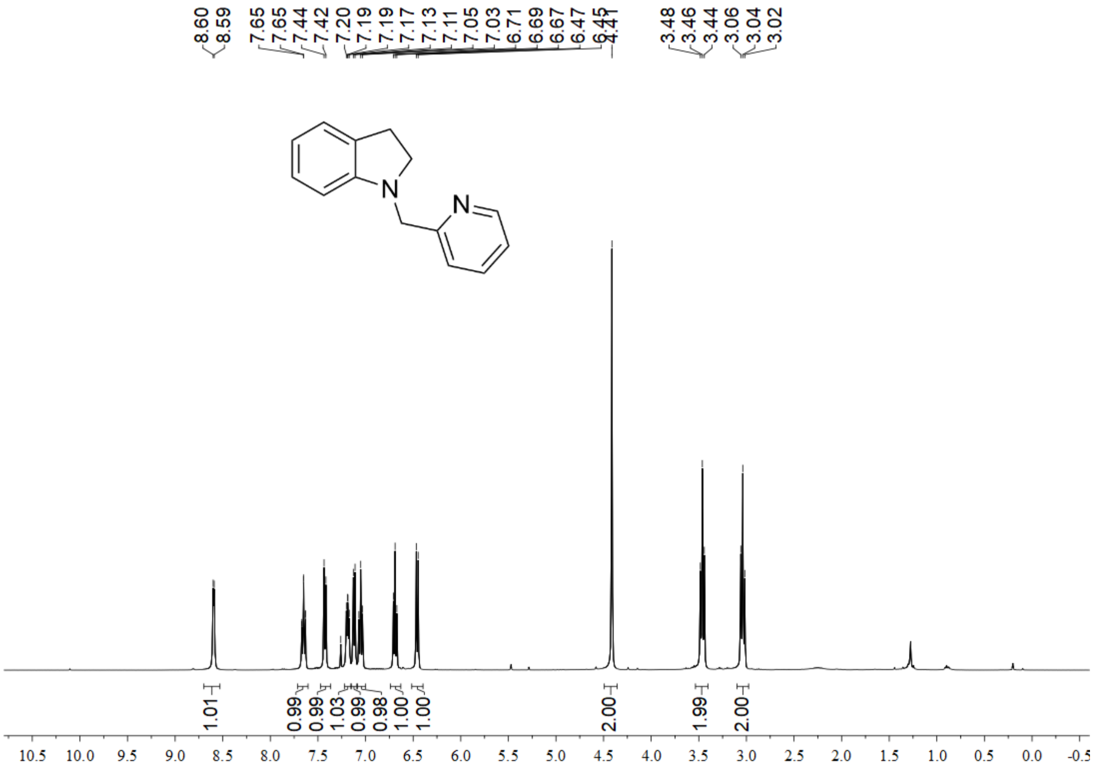

Figure S29 - ^{1}H NMR (400 MHz, $CDCl_3$) of **9m**

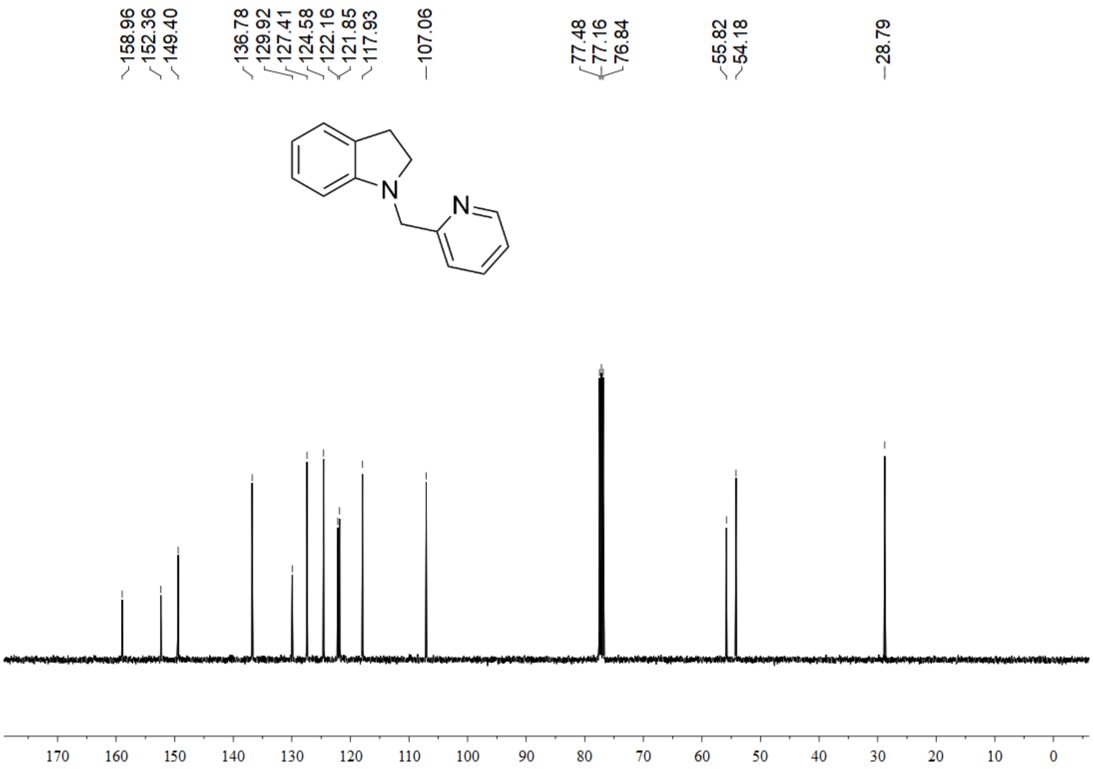

Figure S30 - $^{13}C\{^{1}H\}$ NMR (101 MHz, $CDCl_3$) of **9m**

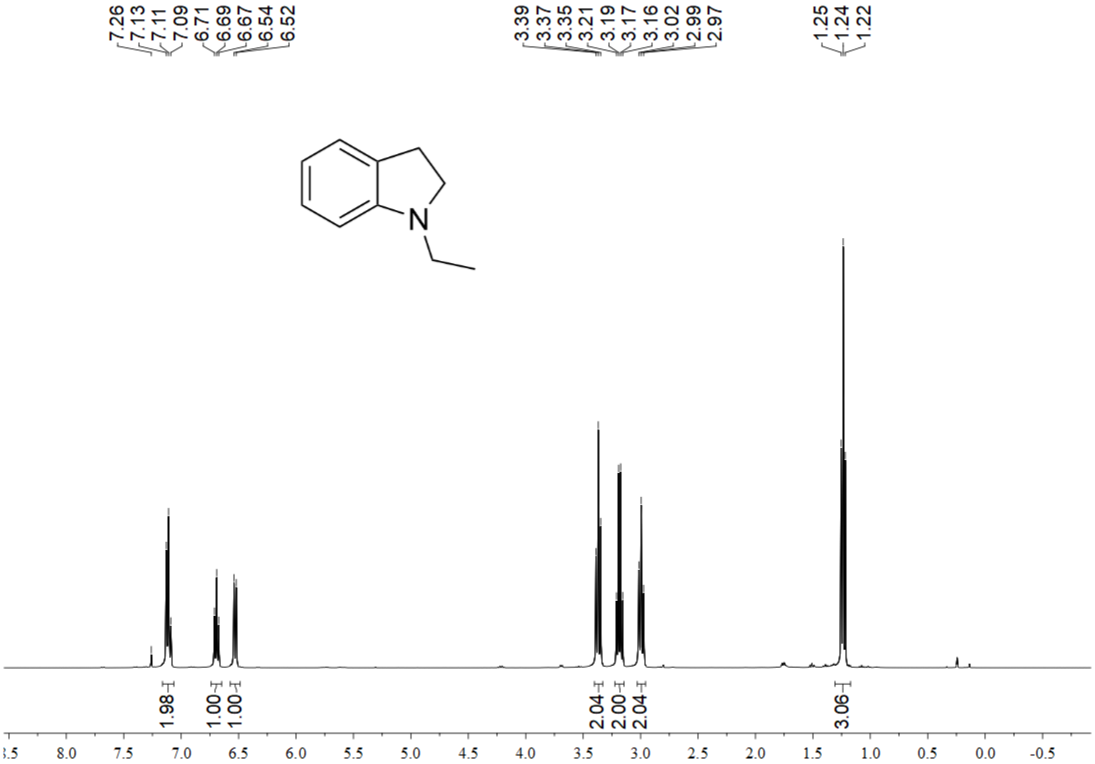

Figure S31 - ^{1}H NMR (400 MHz, $CDCl_3$) of **9n**

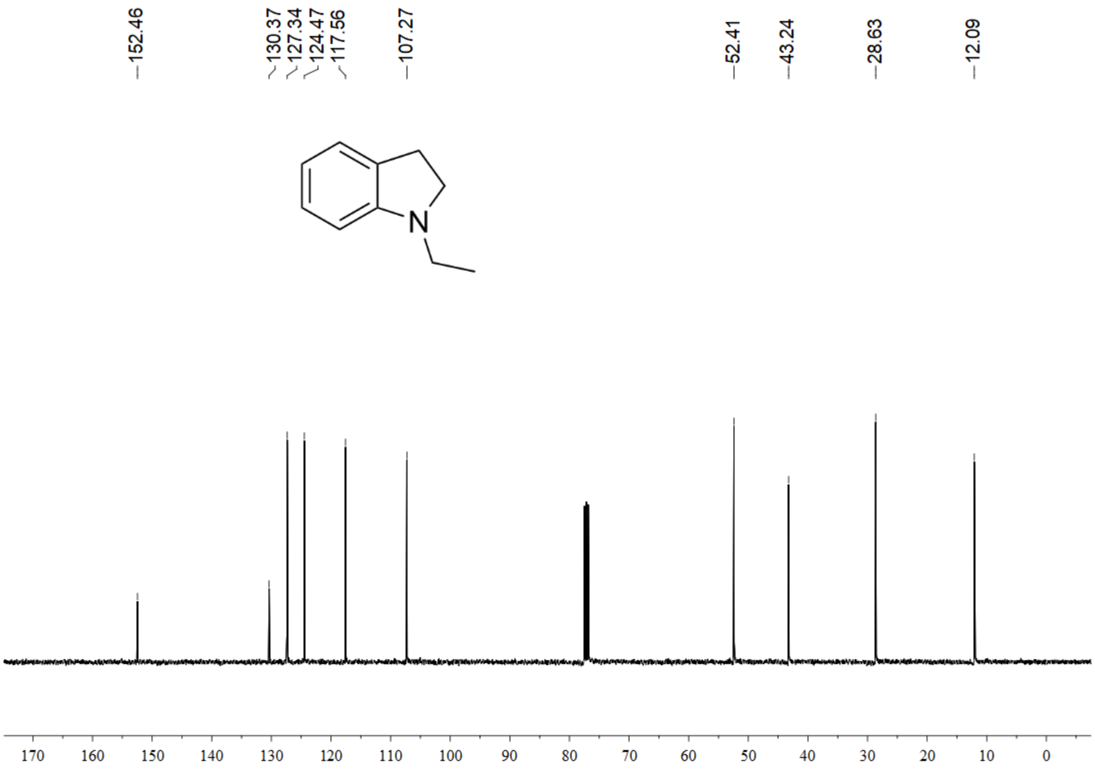

Figure S32 - $^{13}C\{^{1}H\}$ NMR (101 MHz, $CDCl_3$) of **9n**

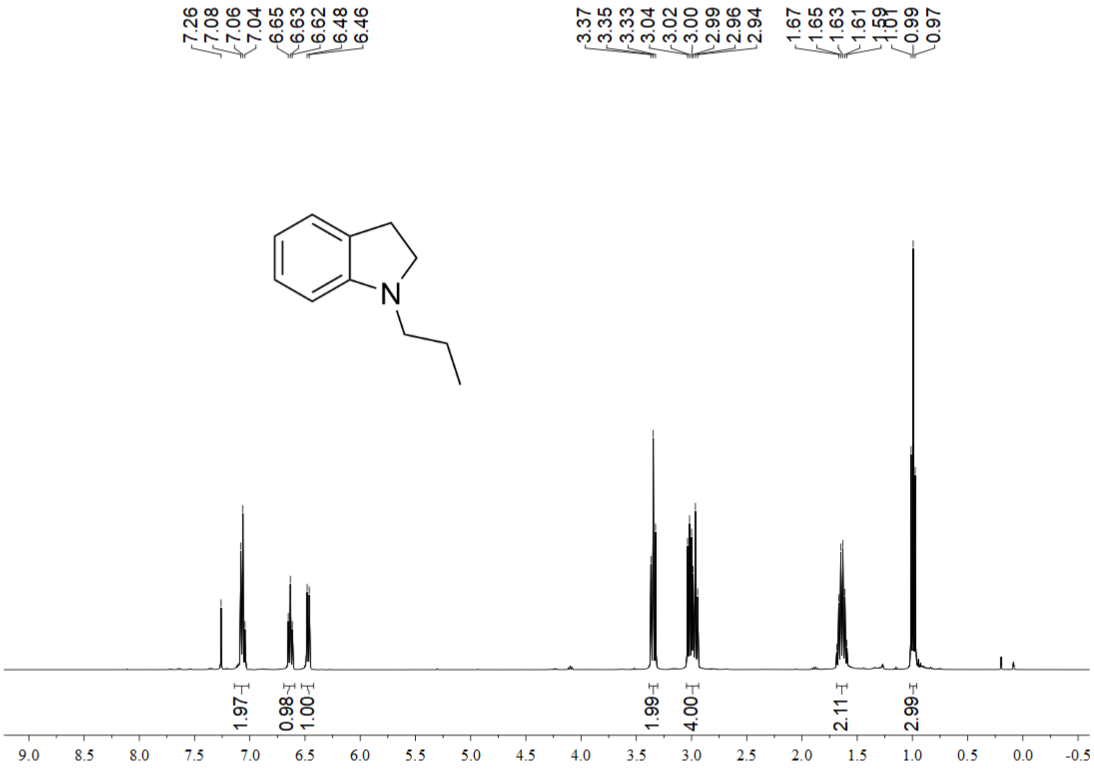

Figure S33 - ^{1}H NMR (400 MHz, $CDCl_3$) of **9o**

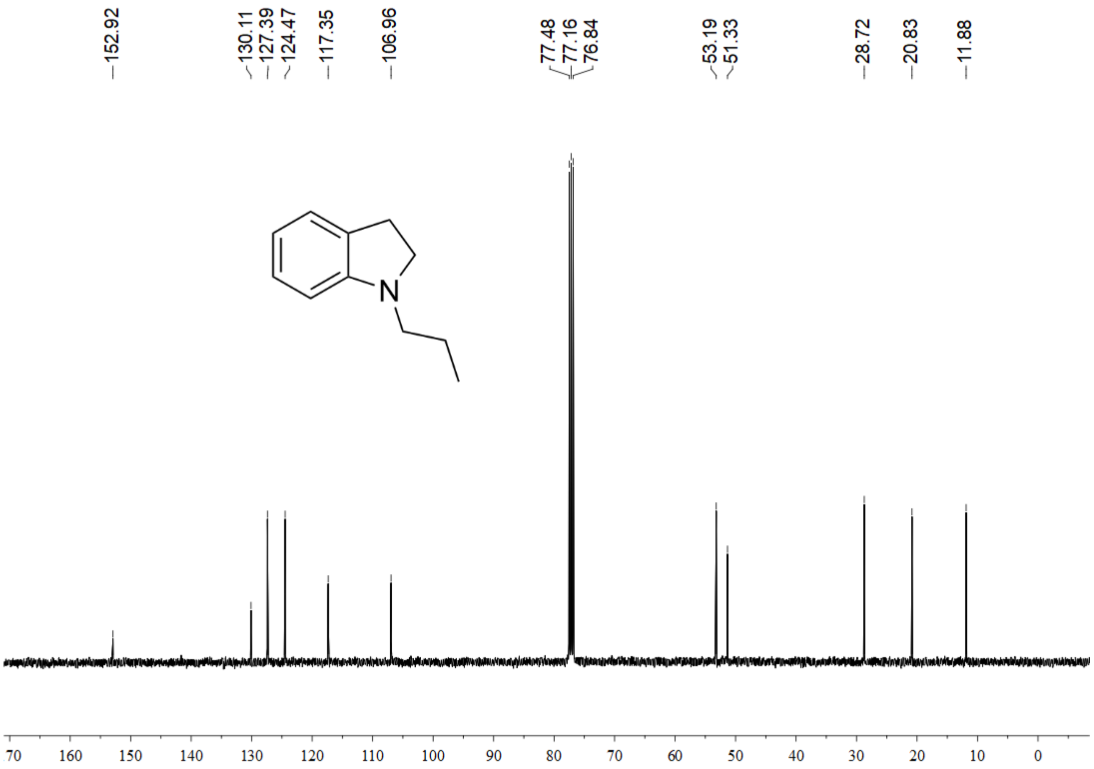

Figure S34 - $^{13}C\{^1H\}$ NMR (101 MHz, $CDCl_3$) of **9o**

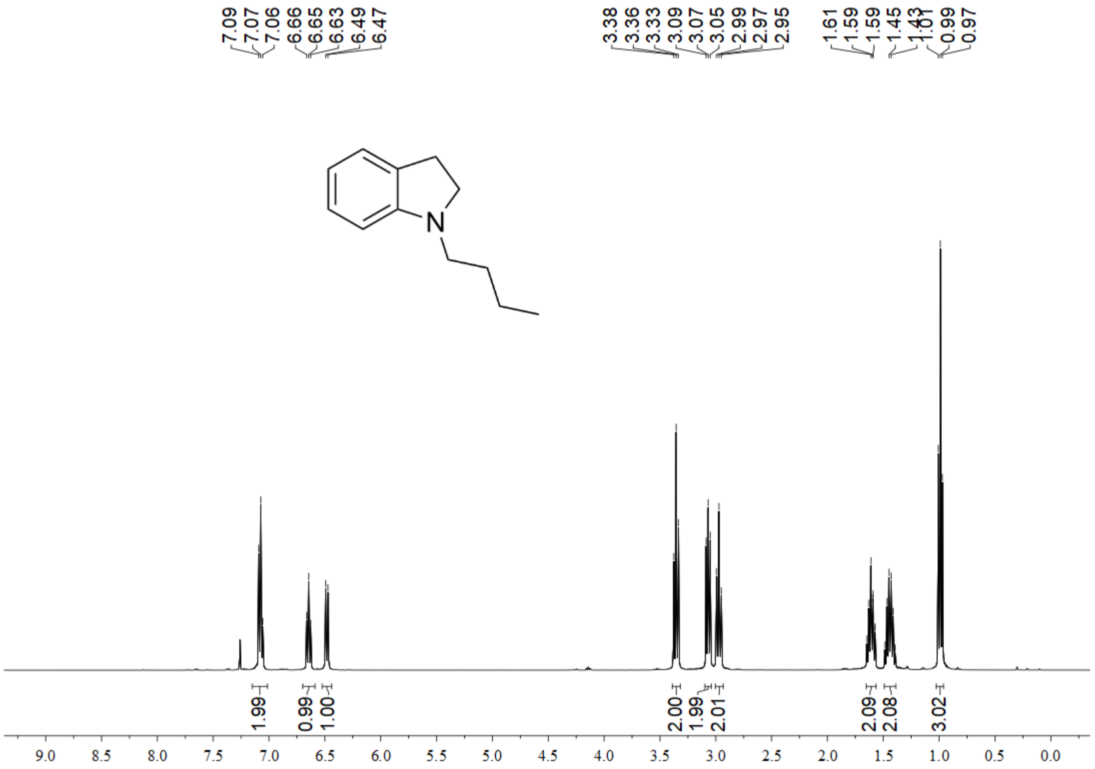

Figure S35 - ^1H NMR (400 MHz, $CDCl_3$) of **9p**

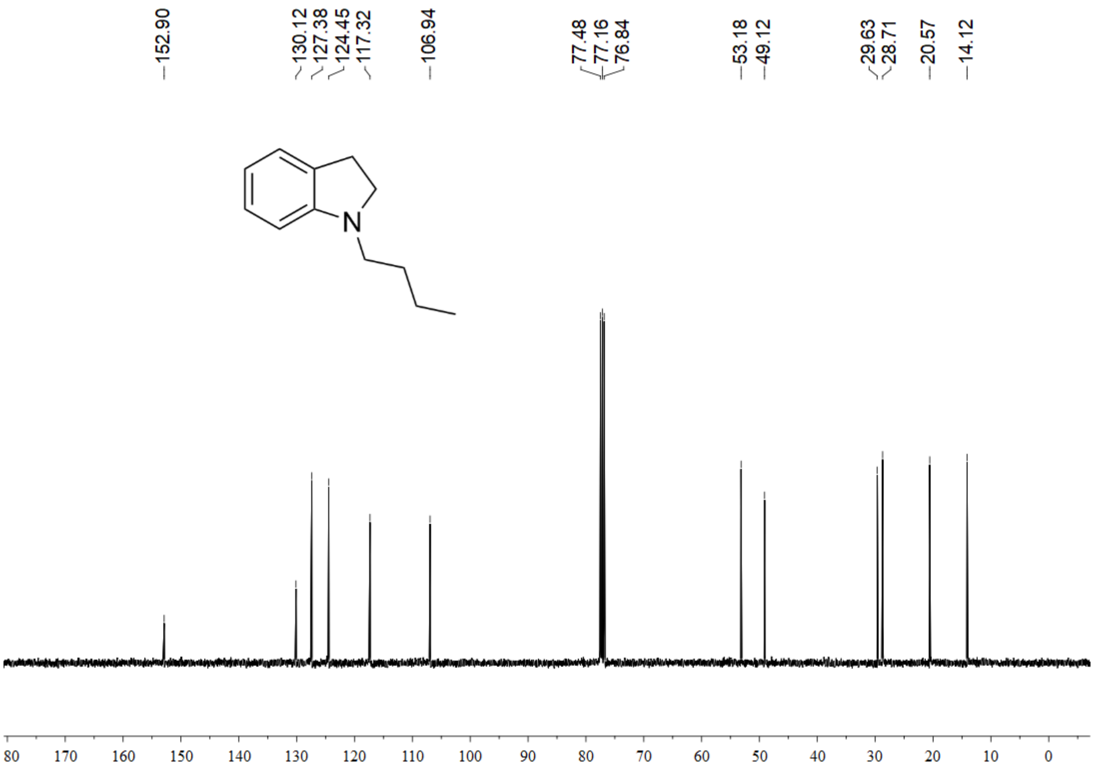

Figure S36 - $^{13}C\{^{1}H\}$ NMR (101 MHz, $CDCl_3$) of **9p**

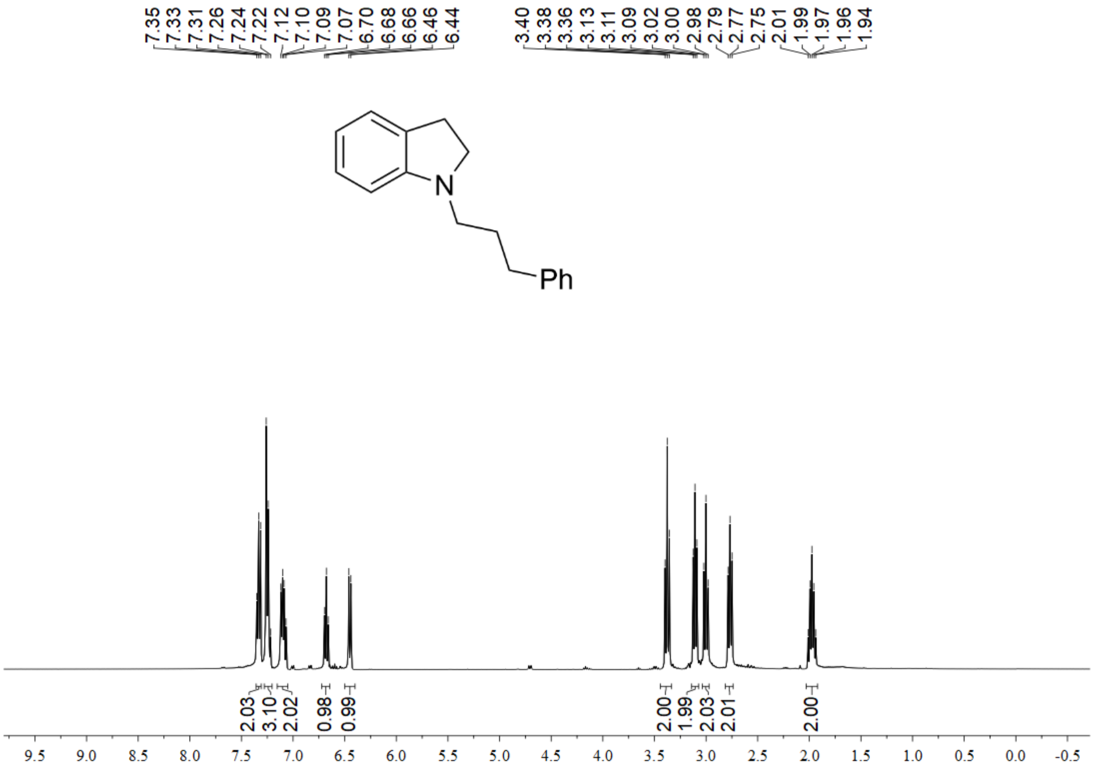

Figure S37 - ^{1}H NMR (400 MHz, $CDCl_3$) of **9q**

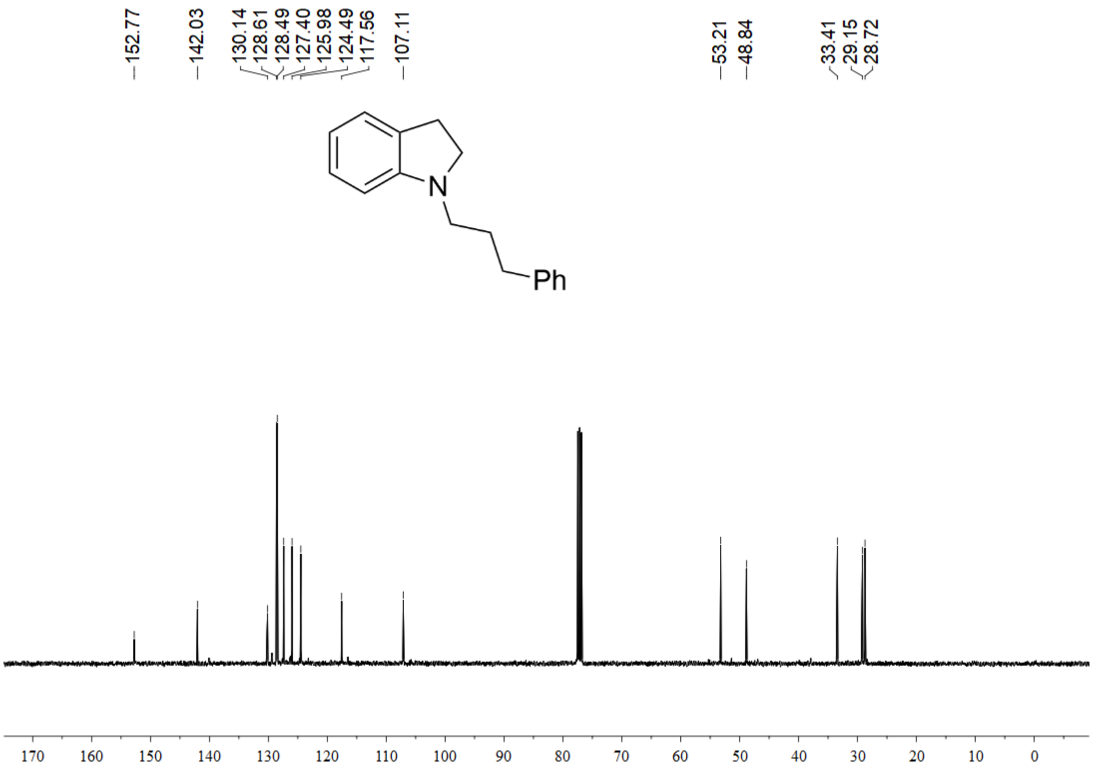

Figure S38 - $^{13}C\{^{1}H\}$ NMR (101 MHz, $CDCl_3$) of **9q**

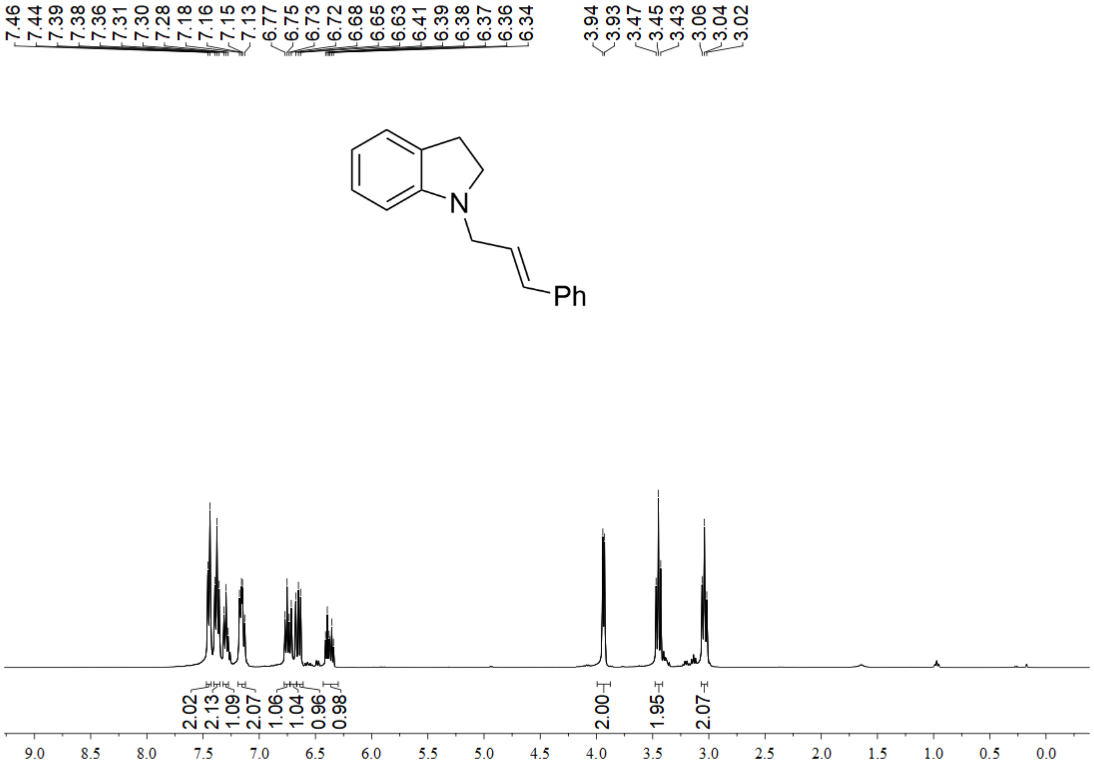

Figure S39 - ^{1}H NMR (400 MHz, $CDCl_3$) of **9r**

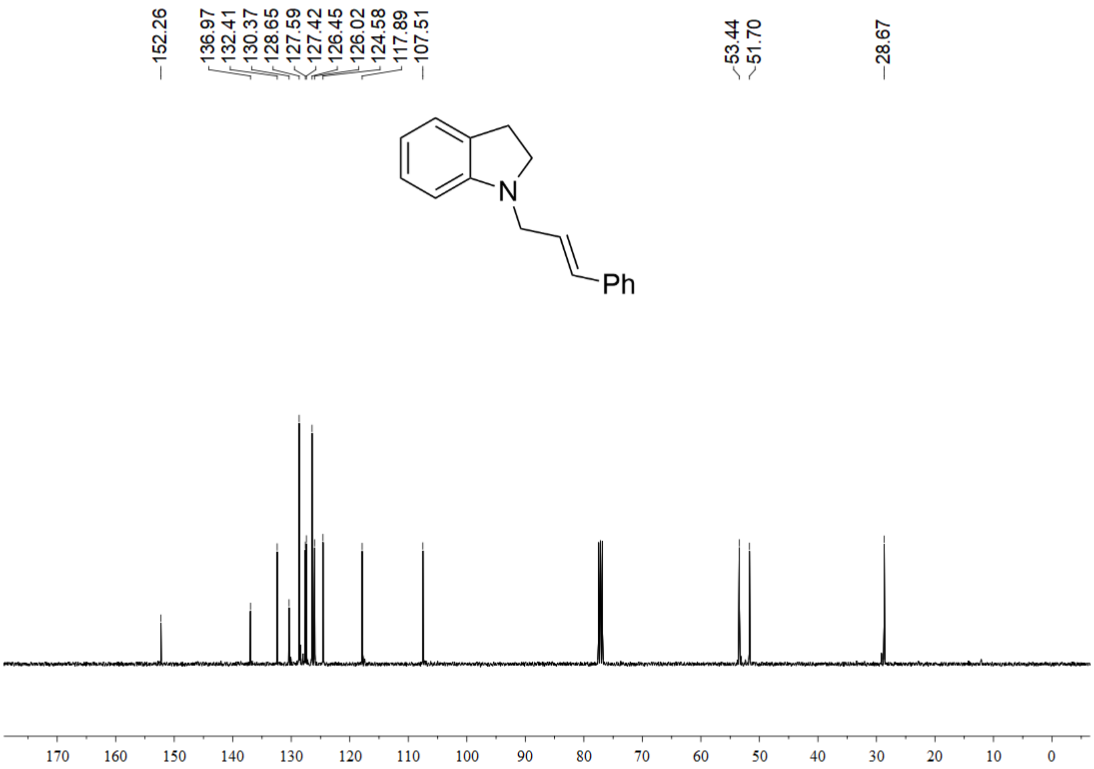

Figure S40 - $^{13}C\{^{1}H\}$ NMR (101 MHz, $CDCl_3$) of **9r**

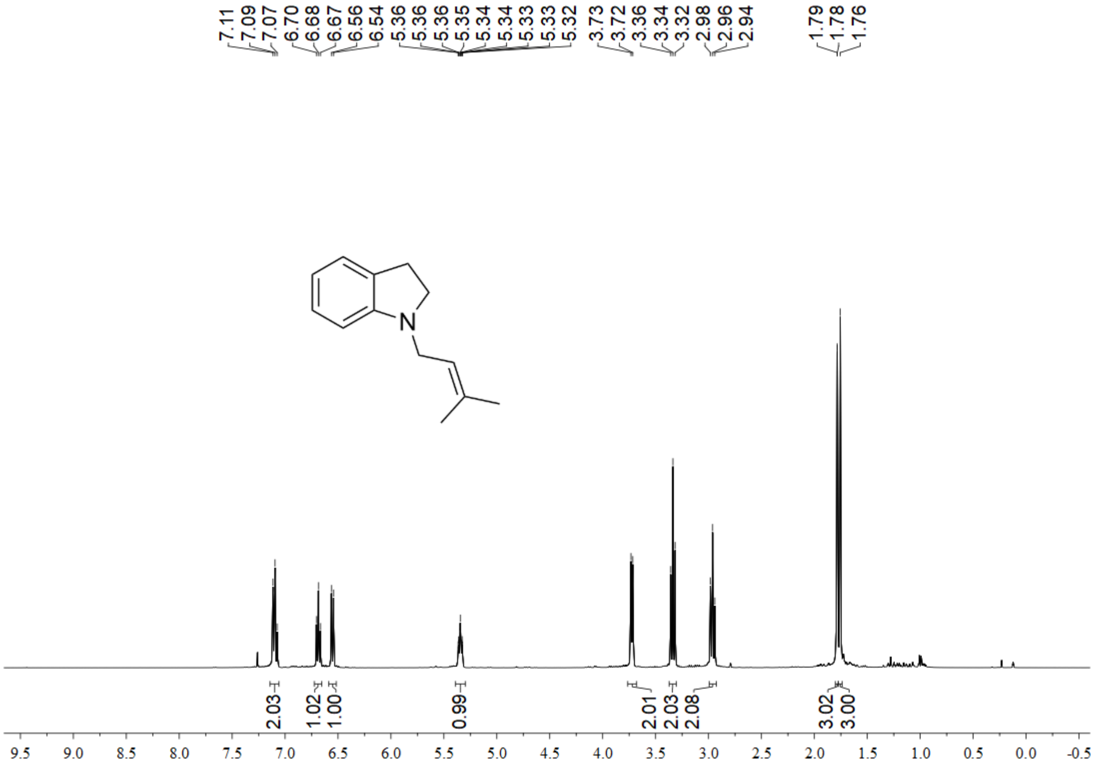

Figure S41 - ^{1}H NMR (400 MHz, $CDCl_3$) of **9s**

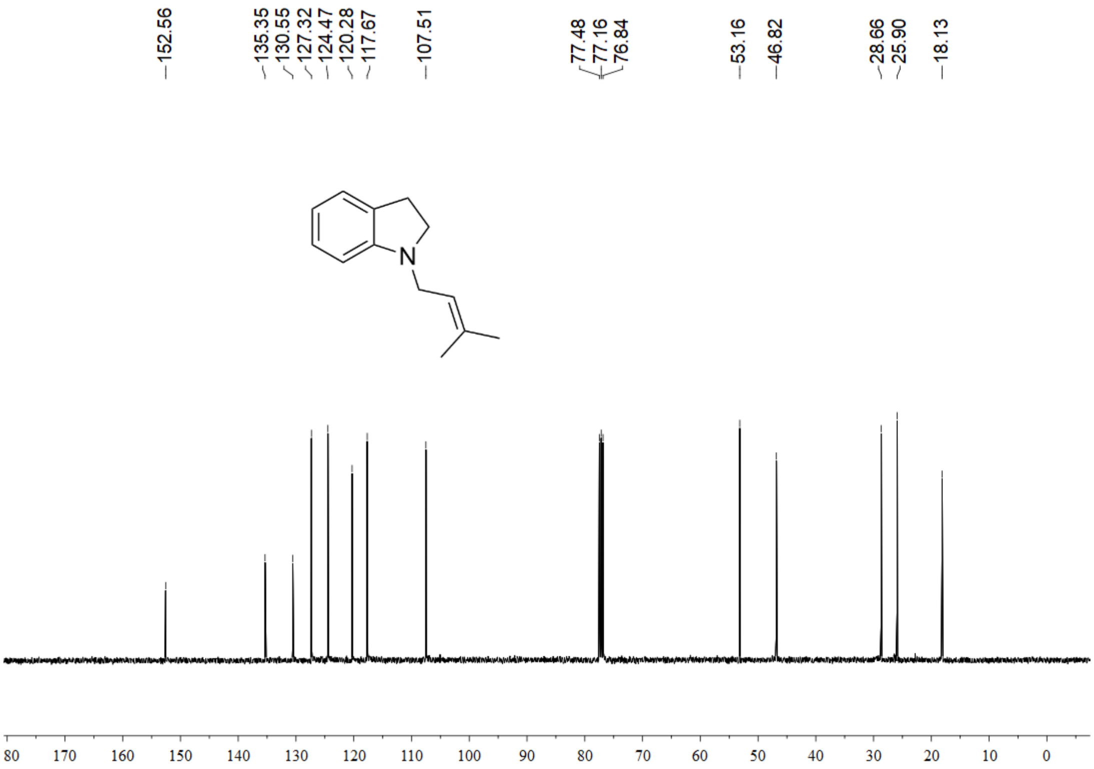

Figure S42 - $^{13}C\{^{1}H\}$ NMR (101 MHz, $CDCl_3$) of **9s**

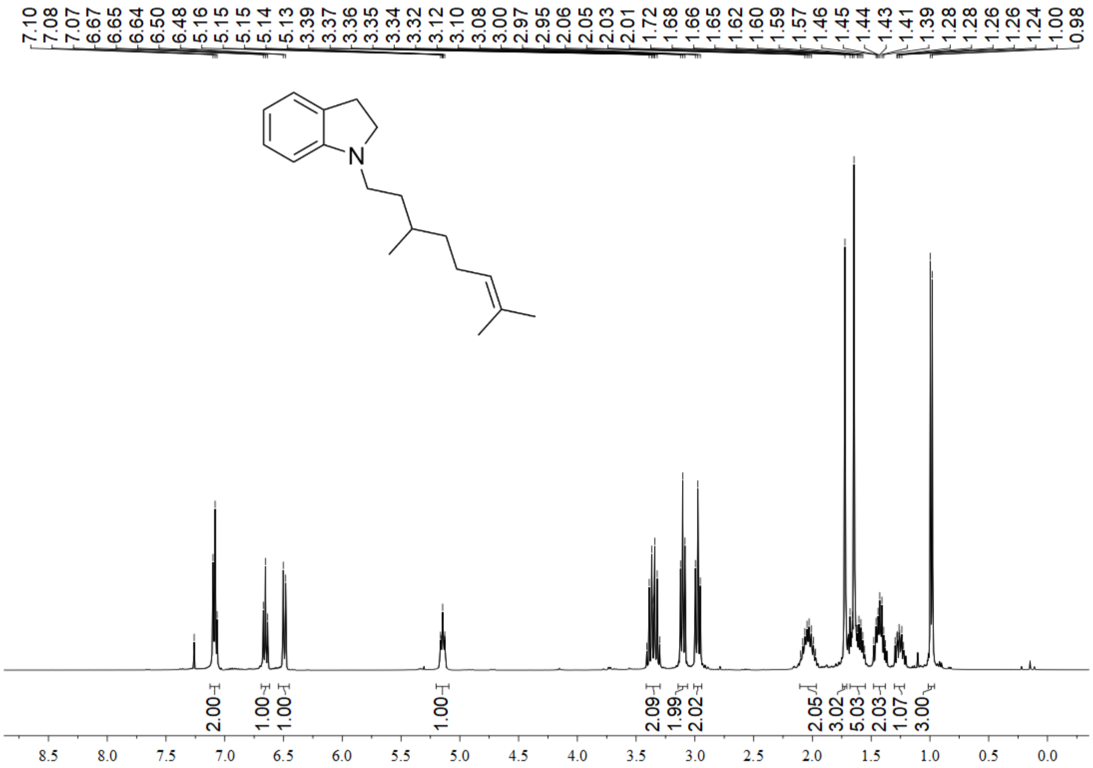

Figure S43 - ^{1}H NMR (400 MHz, $CDCl_3$) of **9t**

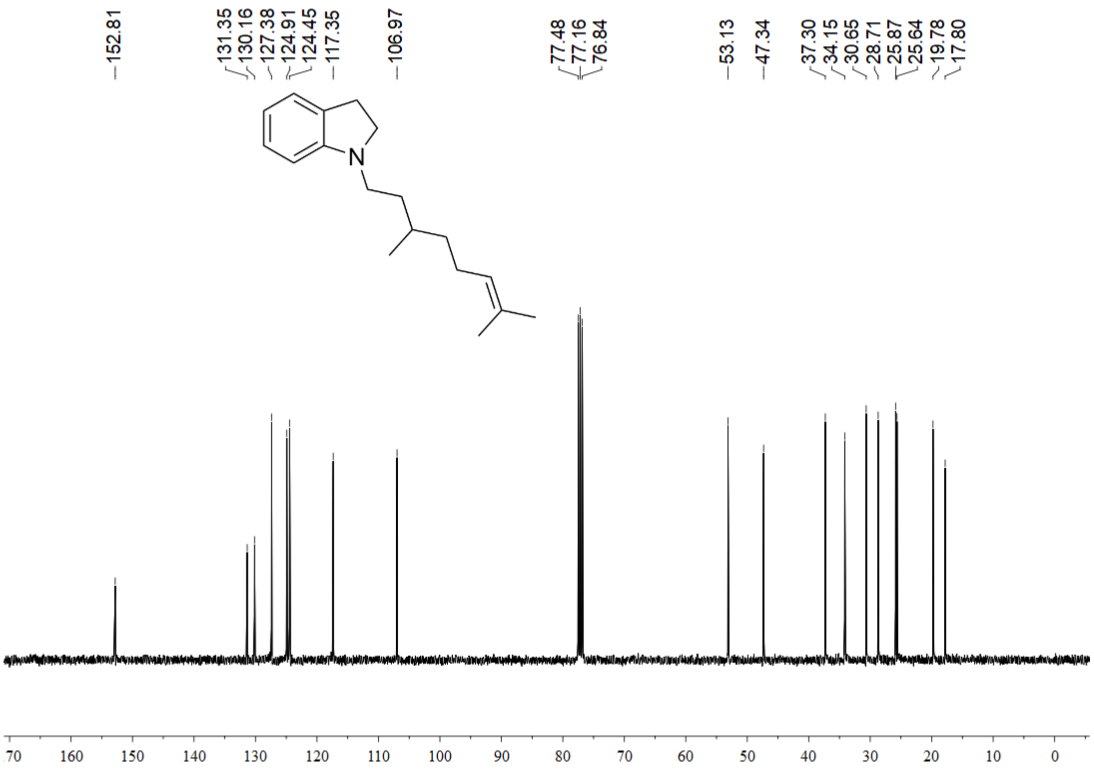

Figure S44 - $^{13}C\{^1H\}$ NMR (101 MHz, $CDCl_3$) of **9t**

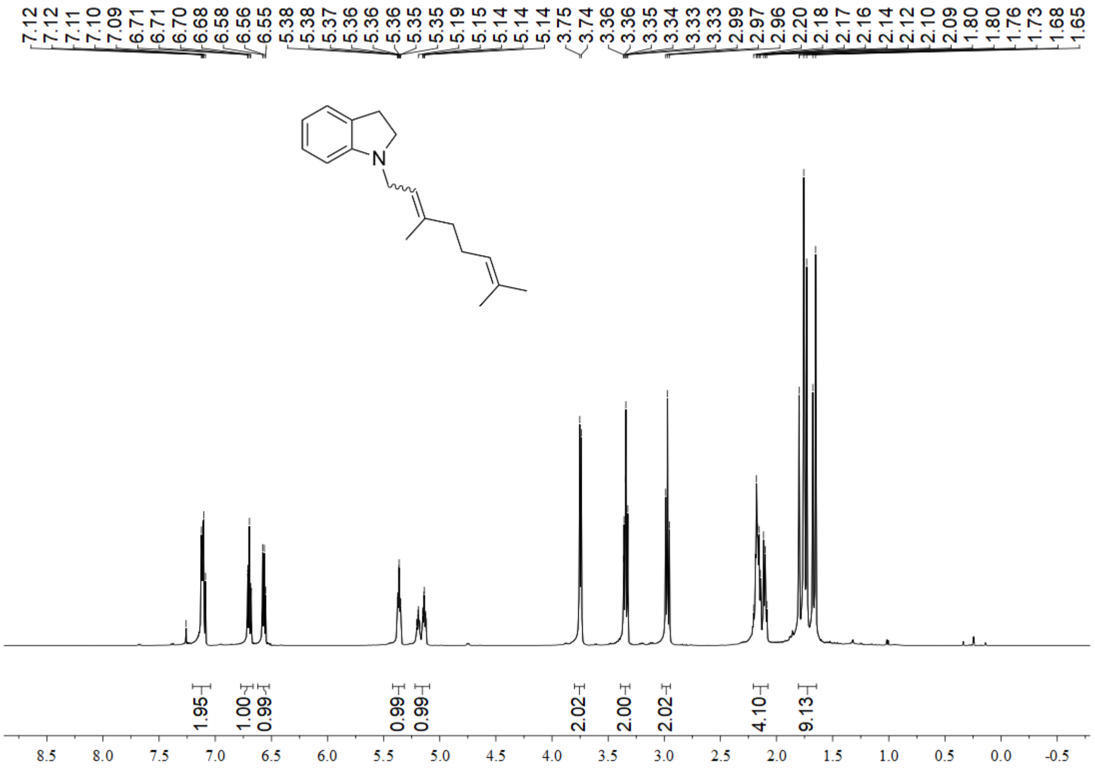

Figure S45 - ^1H NMR (500 MHz, $CDCl_3$) of **9u**

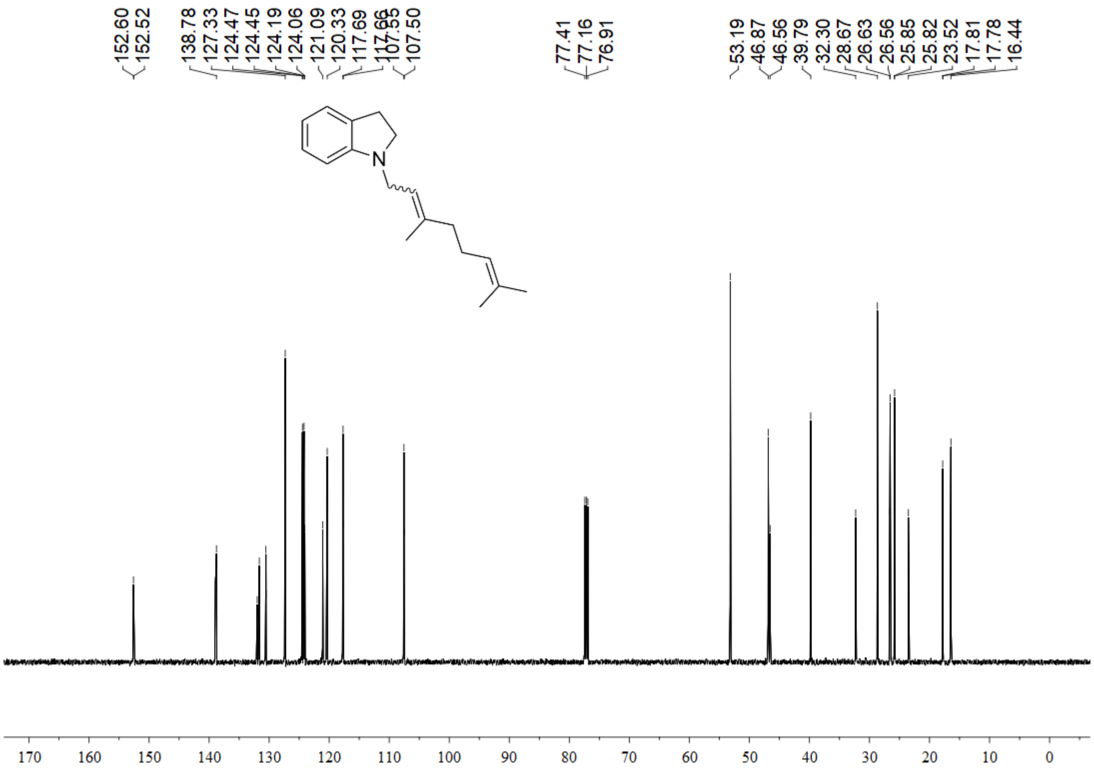

Figure S46 - $^{13}C\{^{1}H\}$ NMR (126 MHz, $CDCl_3$) of **9u**

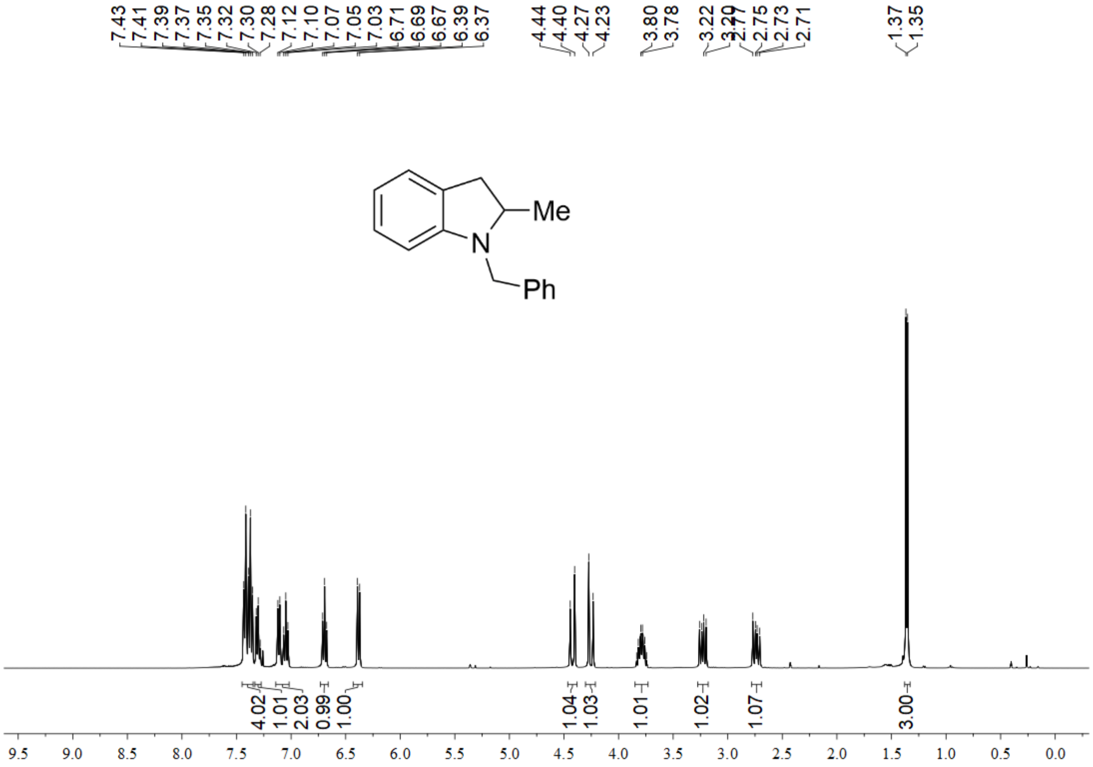

Figure S47 - ^{1}H NMR (400 MHz, $CDCl_3$) of **9v**

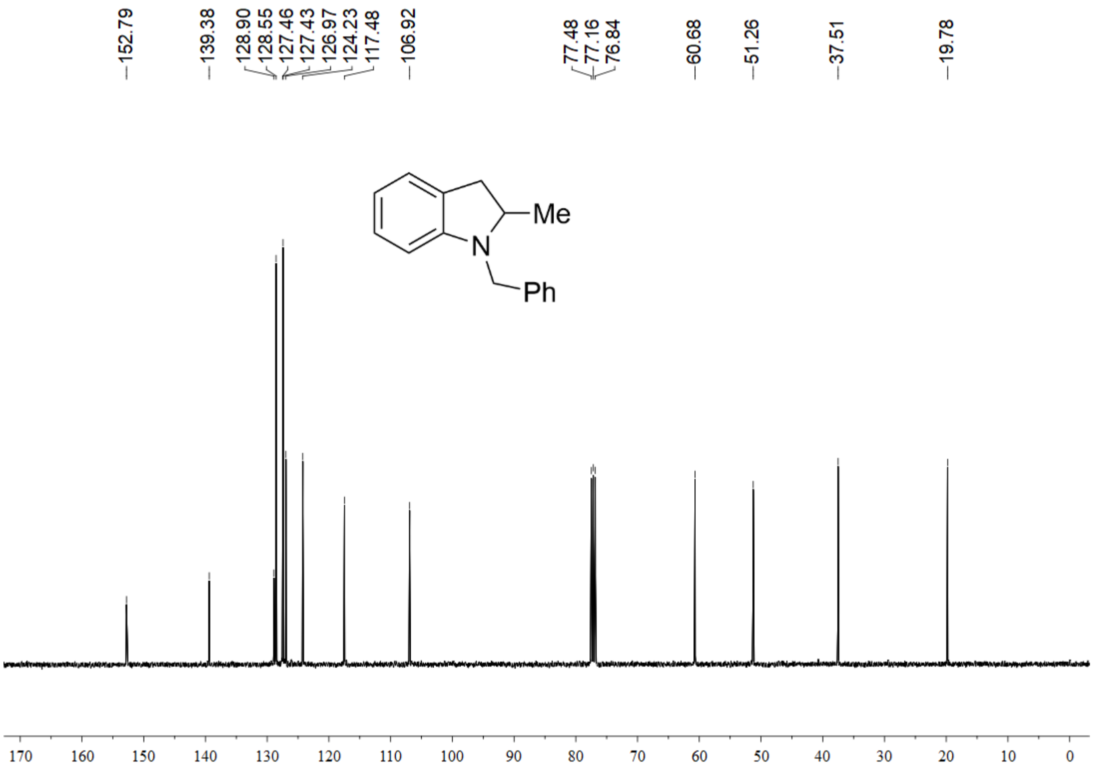

Figure S48 - $^{13}C\{^{1}H\}$ NMR (101 MHz, $CDCl_3$) of **9v**

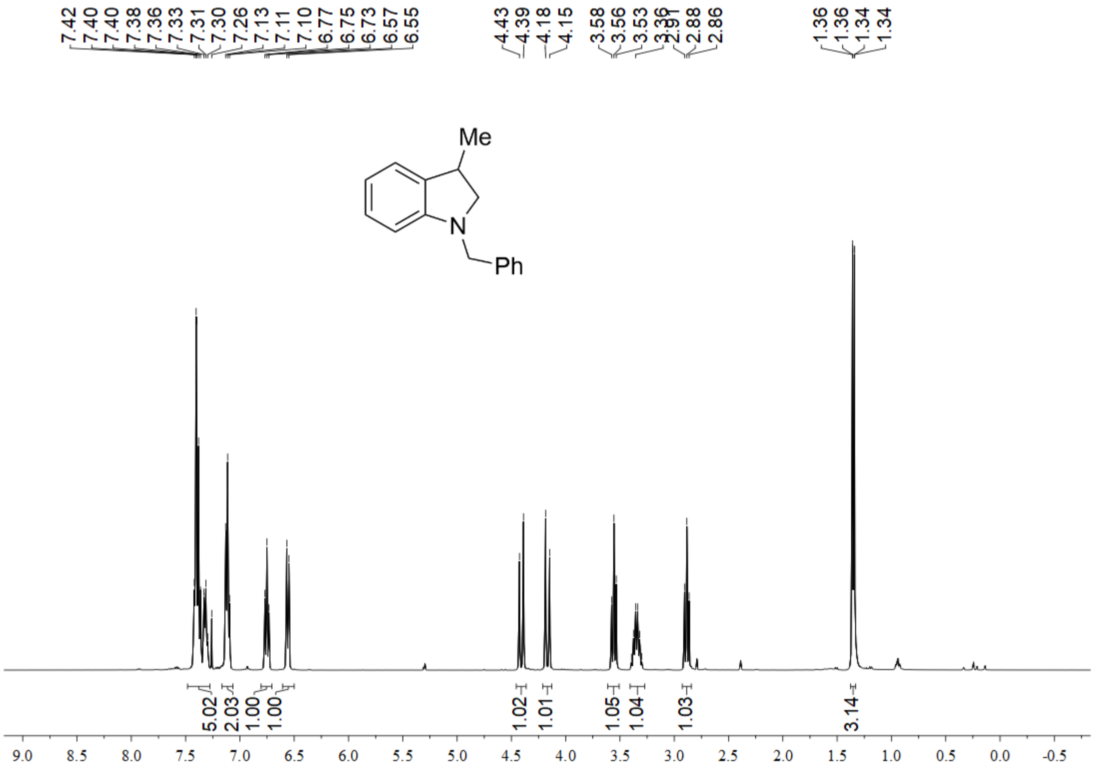

Figure S49 - ^{1}H NMR (400 MHz, $CDCl_3$) of **9w**

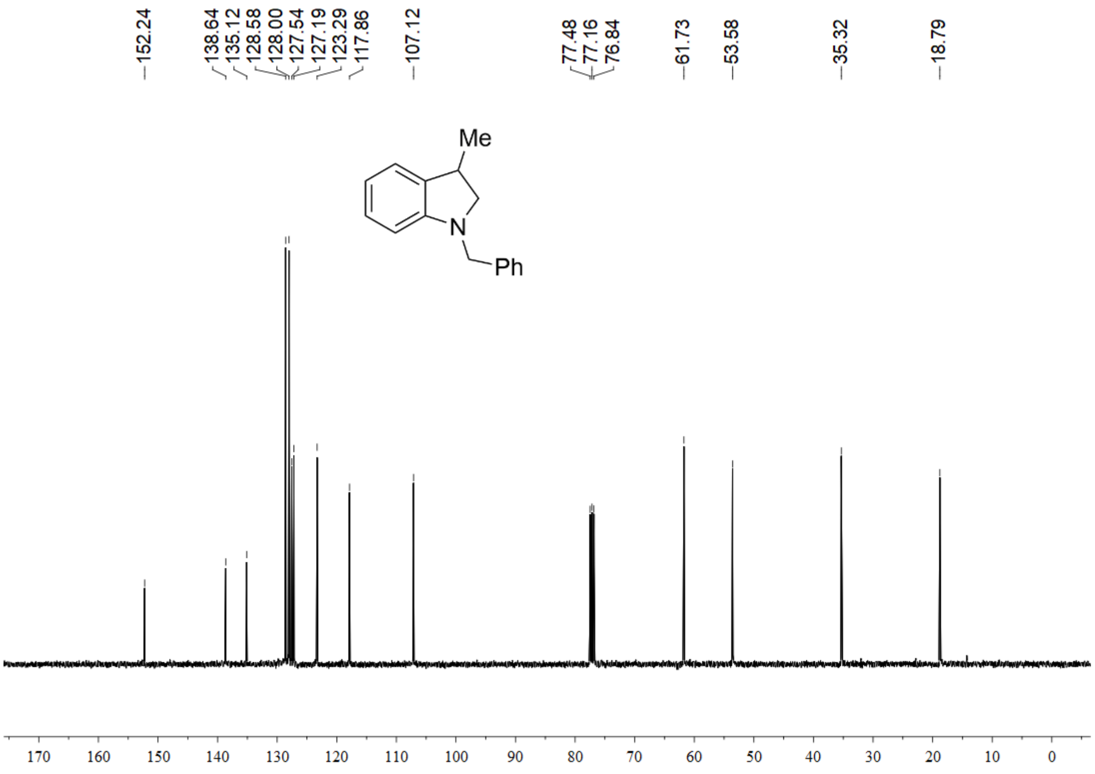

Figure S50 - $^{13}C\{^{1}H\}$ NMR (101 MHz, $CDCl_3$) of **9w**

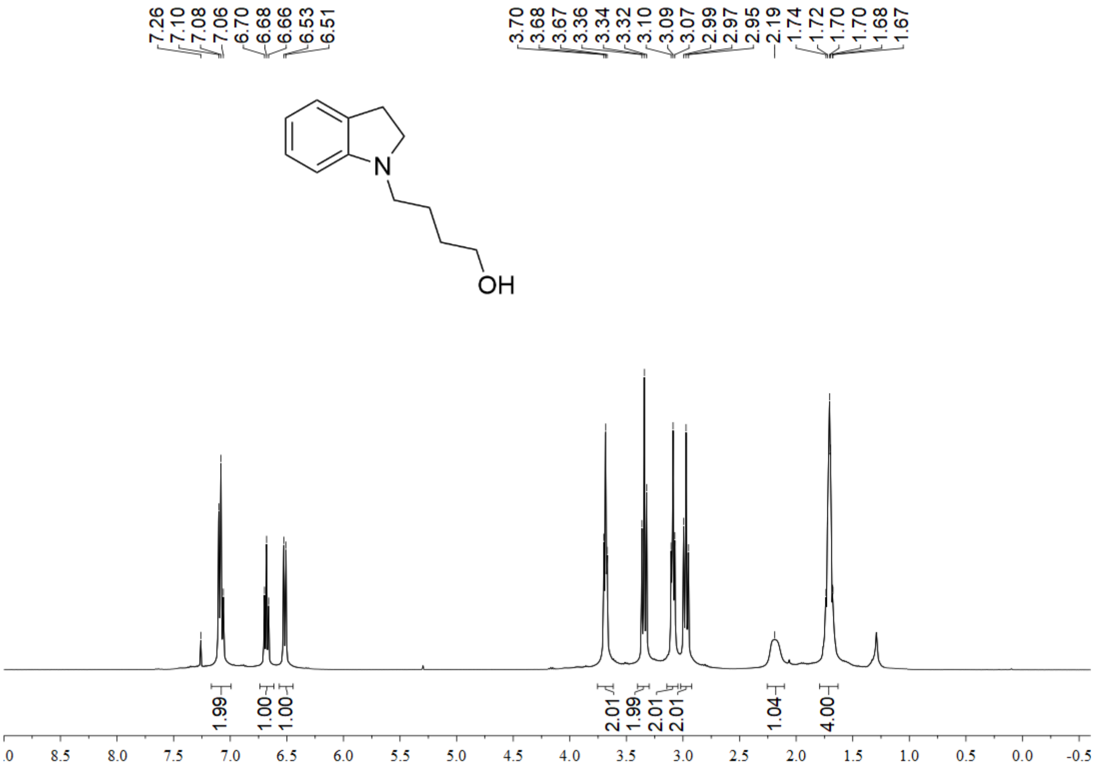

Figure S51 - ^{1}H NMR (400 MHz, $CDCl_3$) of **9x**

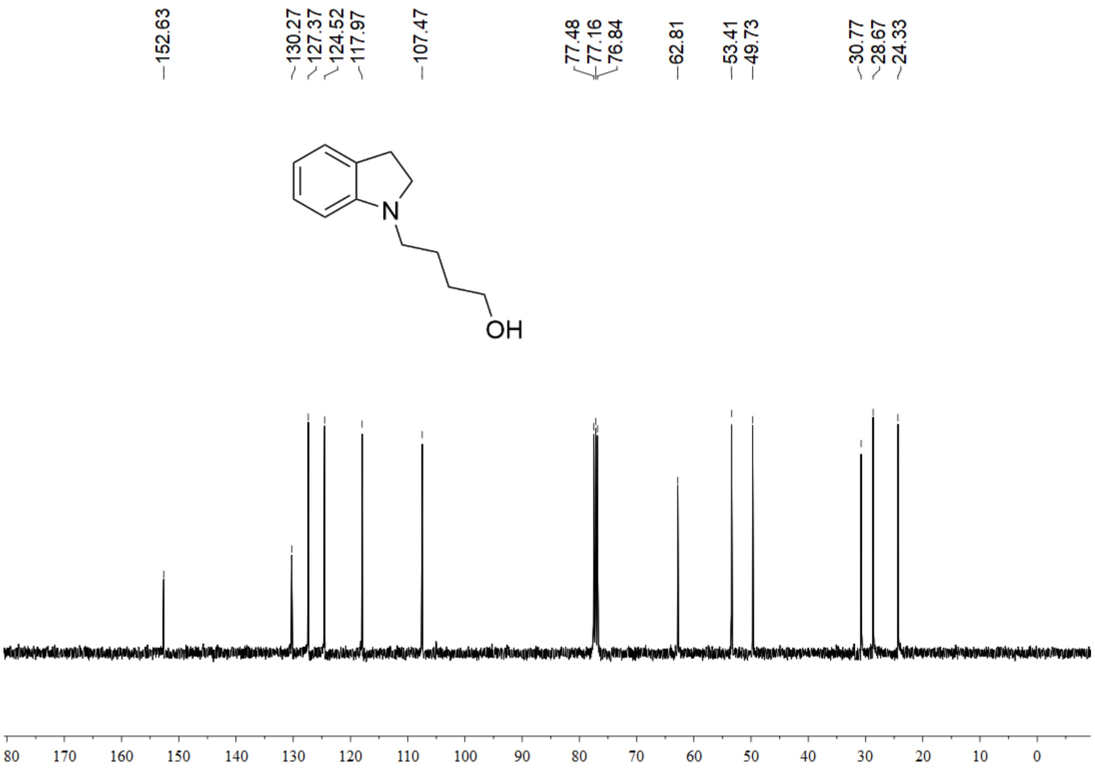

Figure S52 - $^{13}C\{^{1}H\}$ NMR (101 MHz, $CDCl_3$) of **9x**

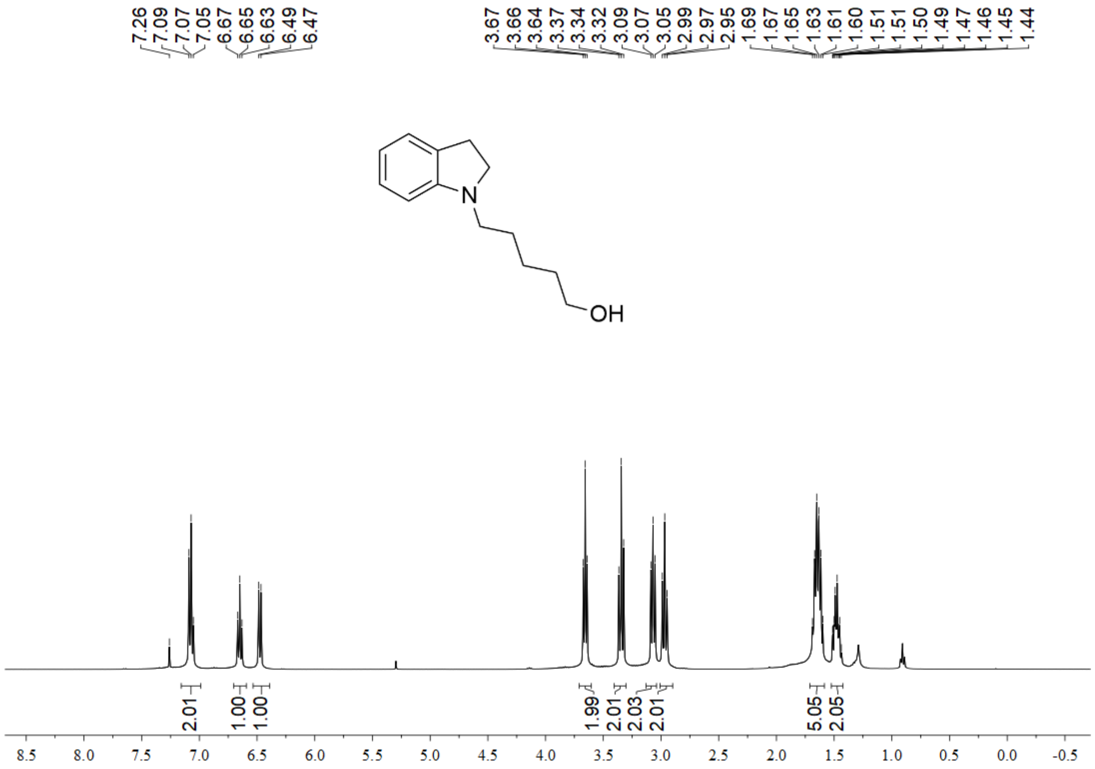

Figure S53 - ^{1}H NMR (400 MHz, $CDCl_3$) of **9y**

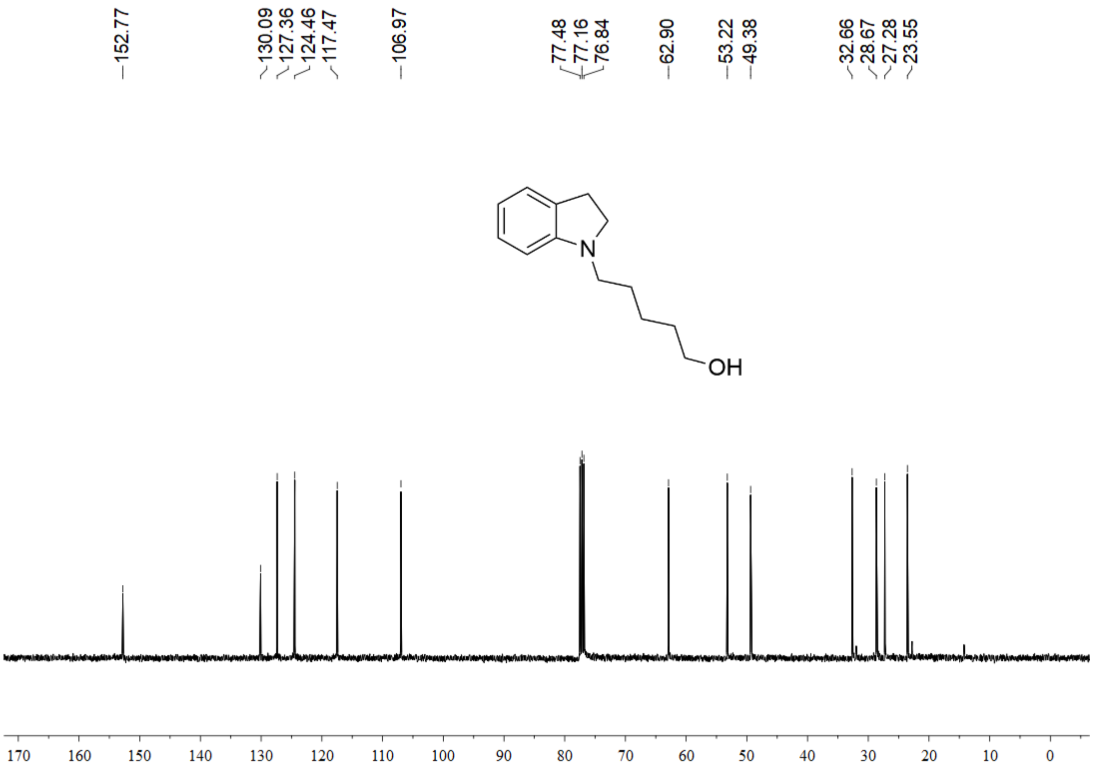

Figure S54 - $^{13}C\{^1H\}$ NMR (101 MHz, $CDCl_3$) of **9y**

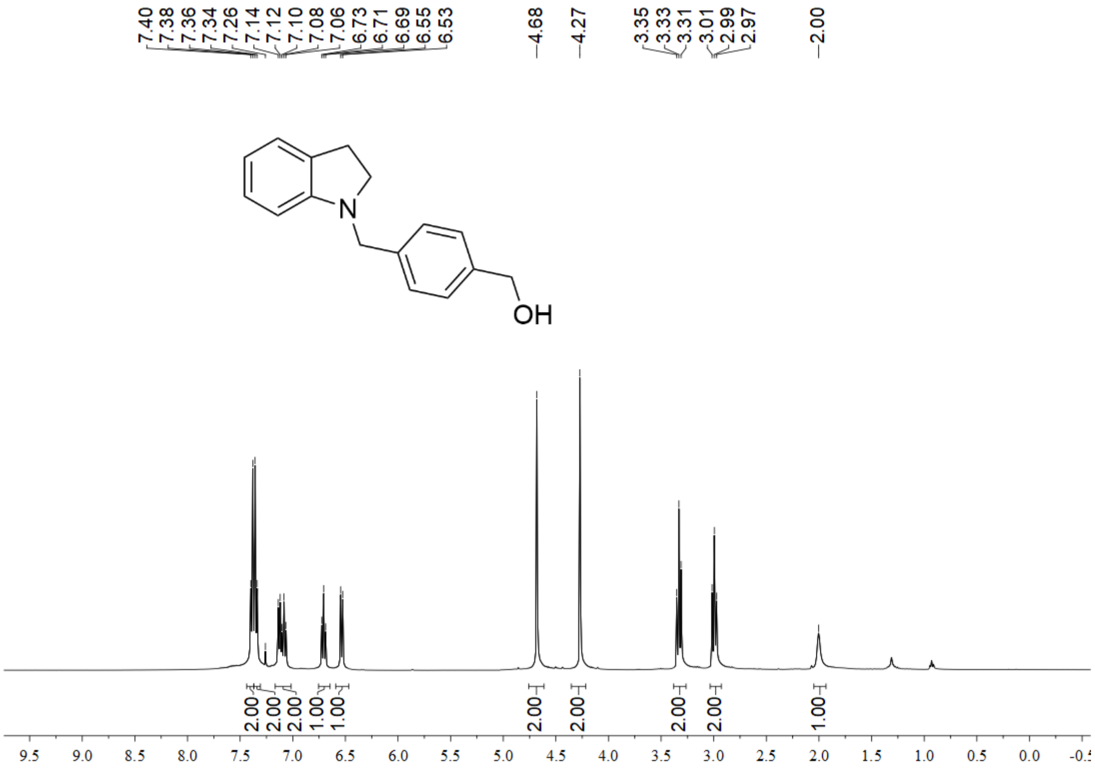

Figure S55 - ^1H NMR (400 MHz, $CDCl_3$) of **9z**

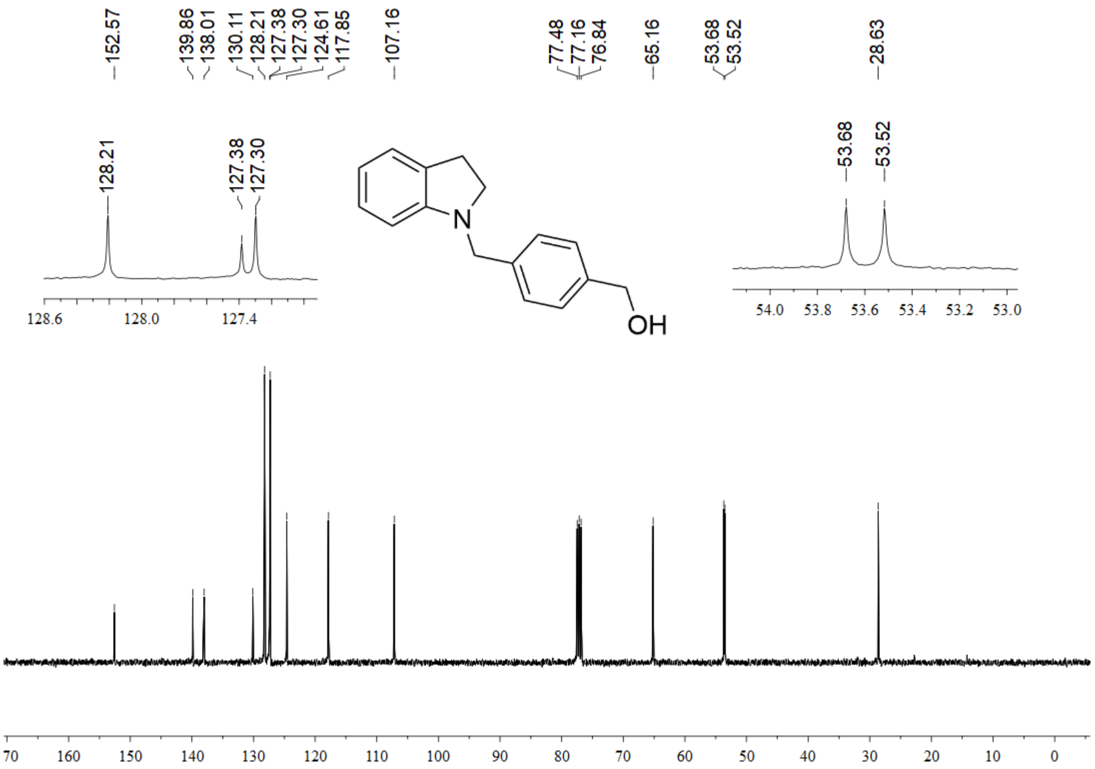

Figure S56 - $^{13}C\{^{1}H\}$ NMR (101 MHz, $CDCl_3$) of **9z**

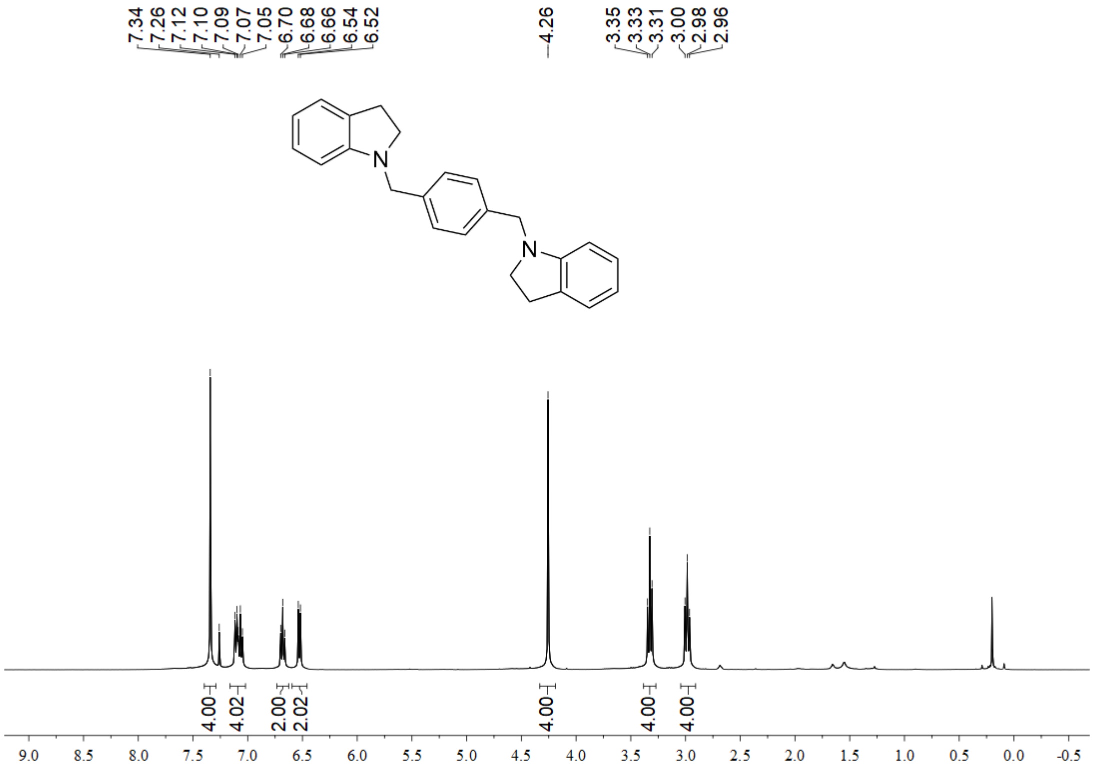

Figure S57 - ^{1}H NMR (400 MHz, $CDCl_3$) of **9aa**

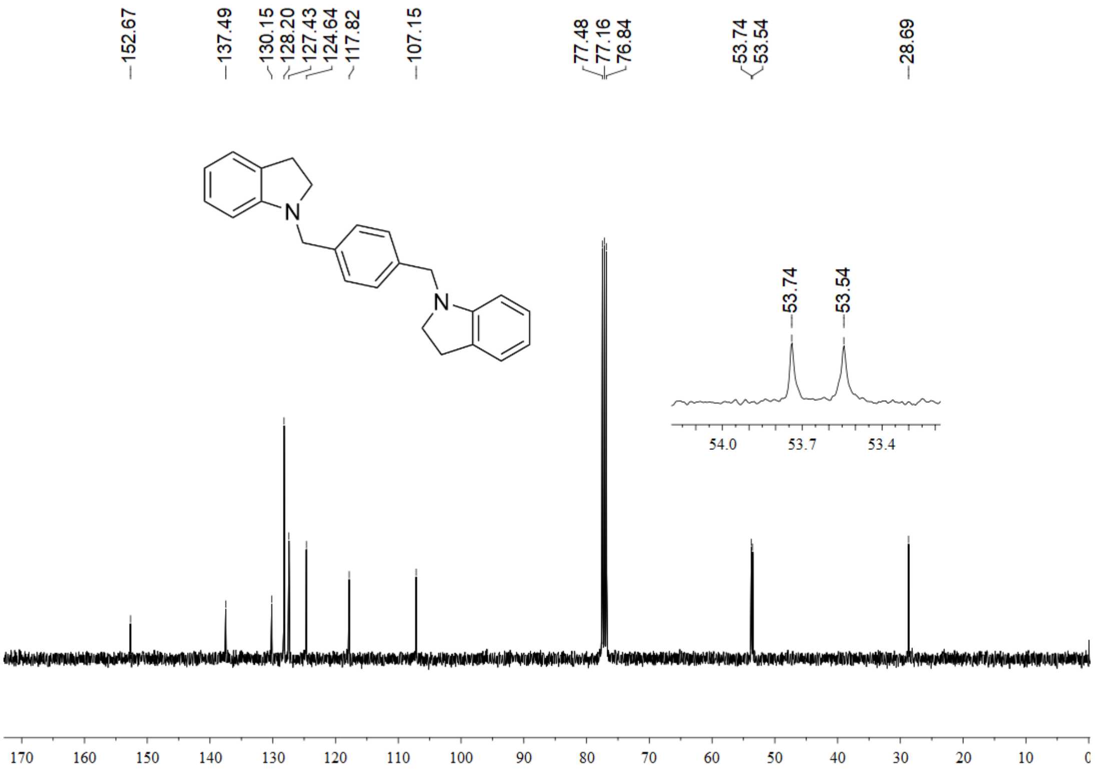

Figure S58 - $^{13}C\{^1H\}$ NMR (101 MHz, $CDCl_3$) of **9aa**

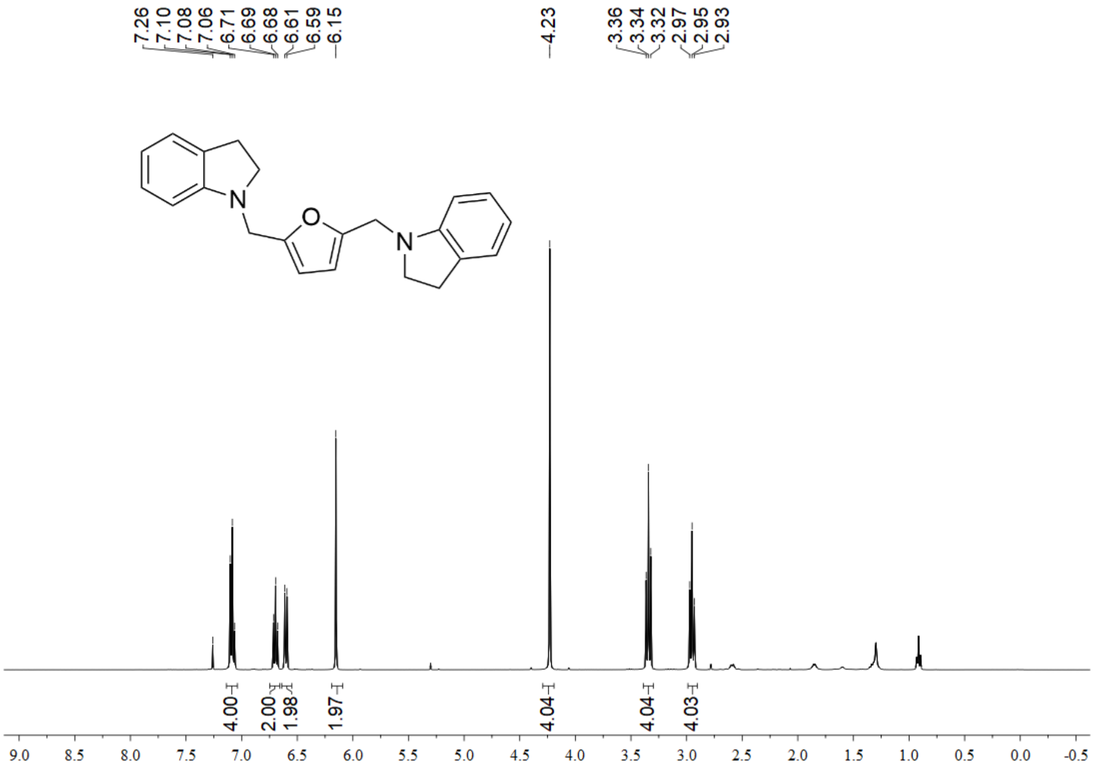

Figure S59 - ^1H NMR (400 MHz, $CDCl_3$) of **9ab**

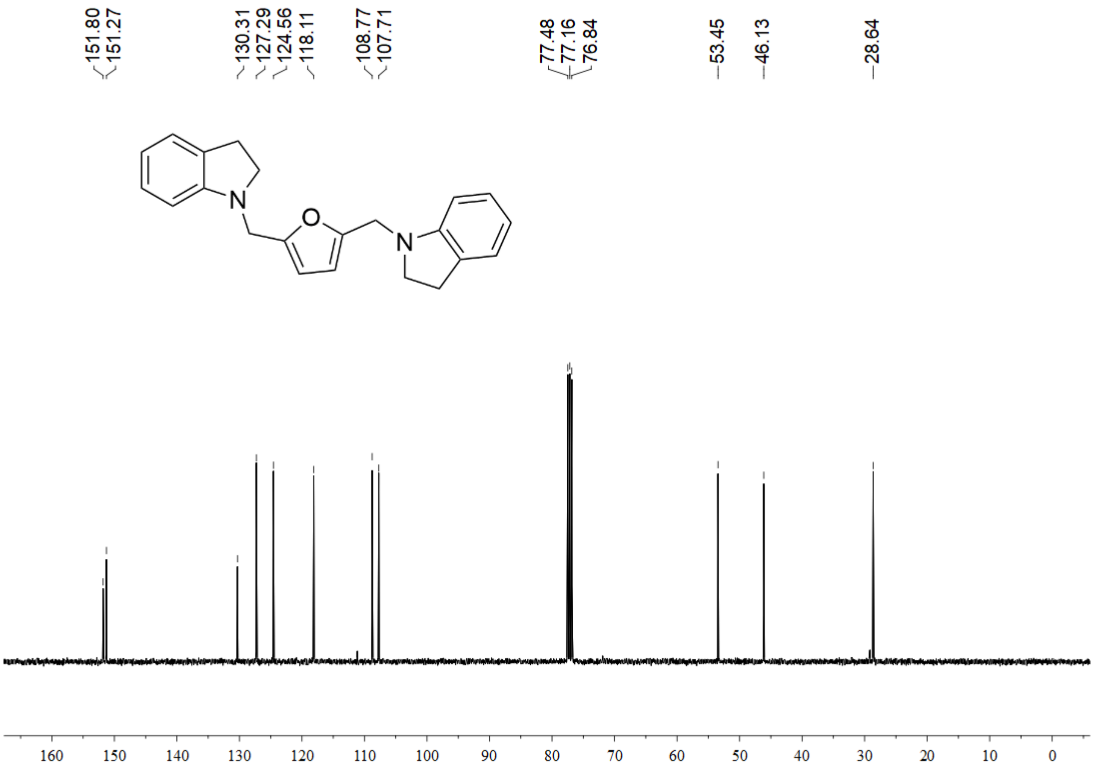

Figure S60 - $^{13}C\{^{1}H\}$ NMR (101 MHz, $CDCl_3$) of **9ab**

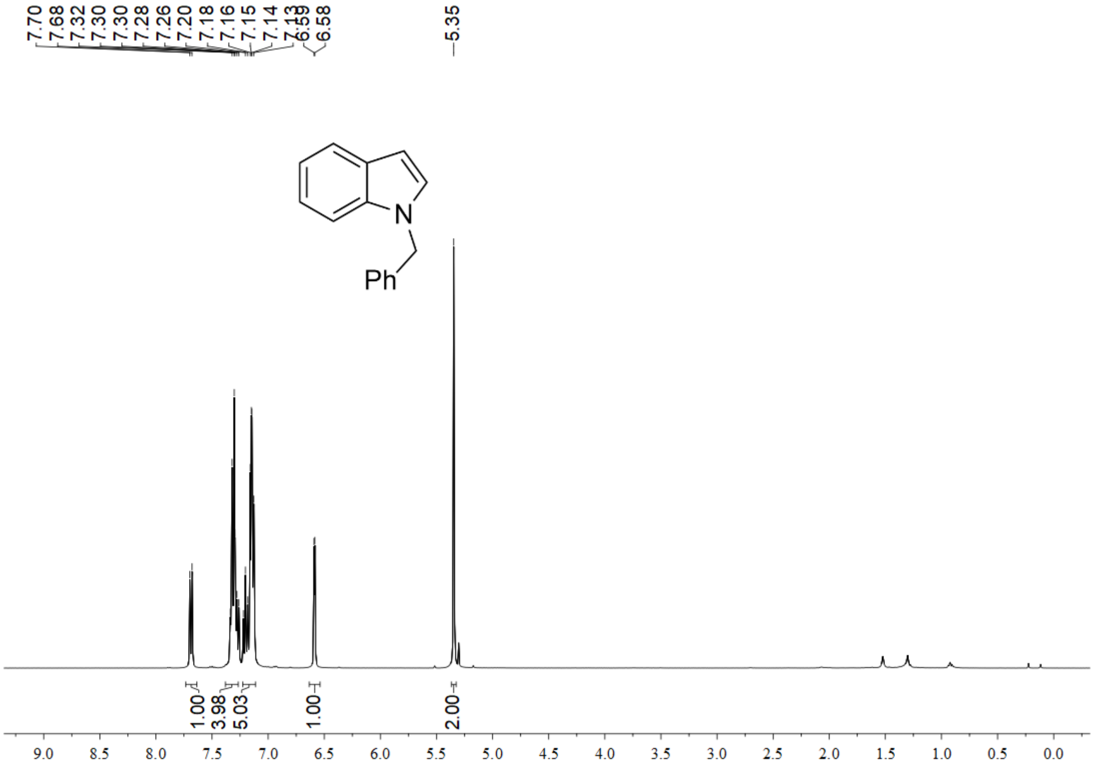

Figure S61 - ^{1}H NMR (400 MHz, $CDCl_3$) of **10a**

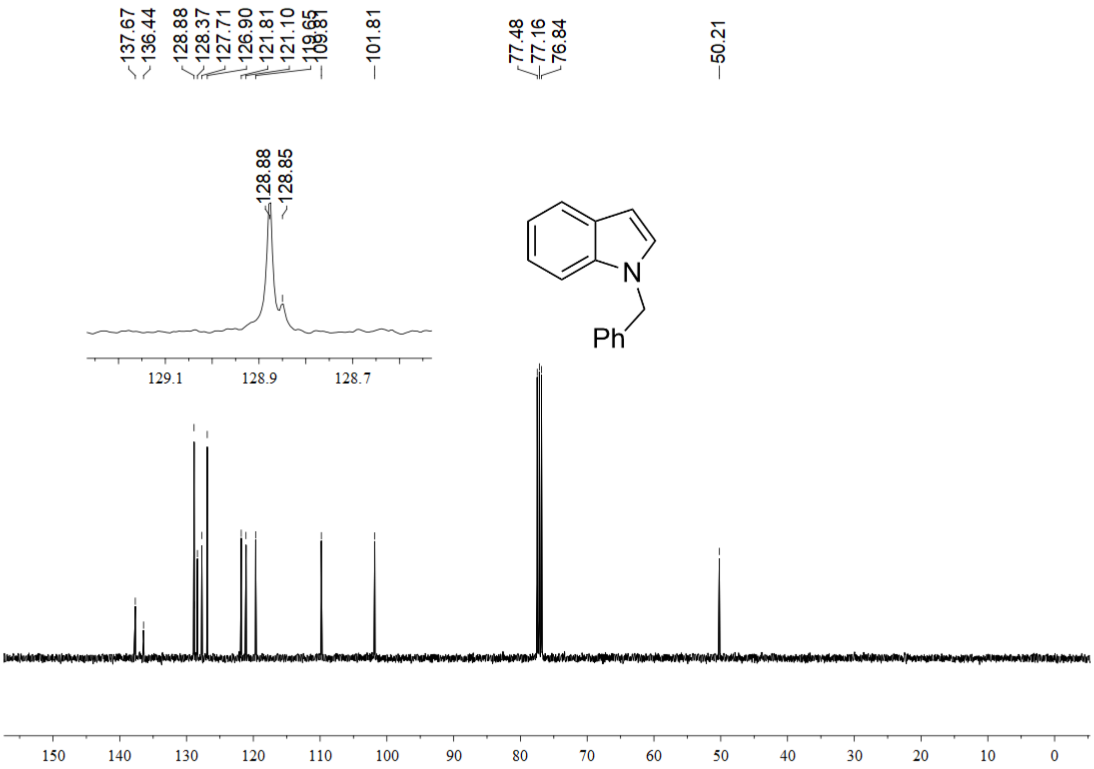

Figure S62 - $^{13}C\{^{1}H\}$ NMR (101 MHz, $CDCl_3$) of **10a**

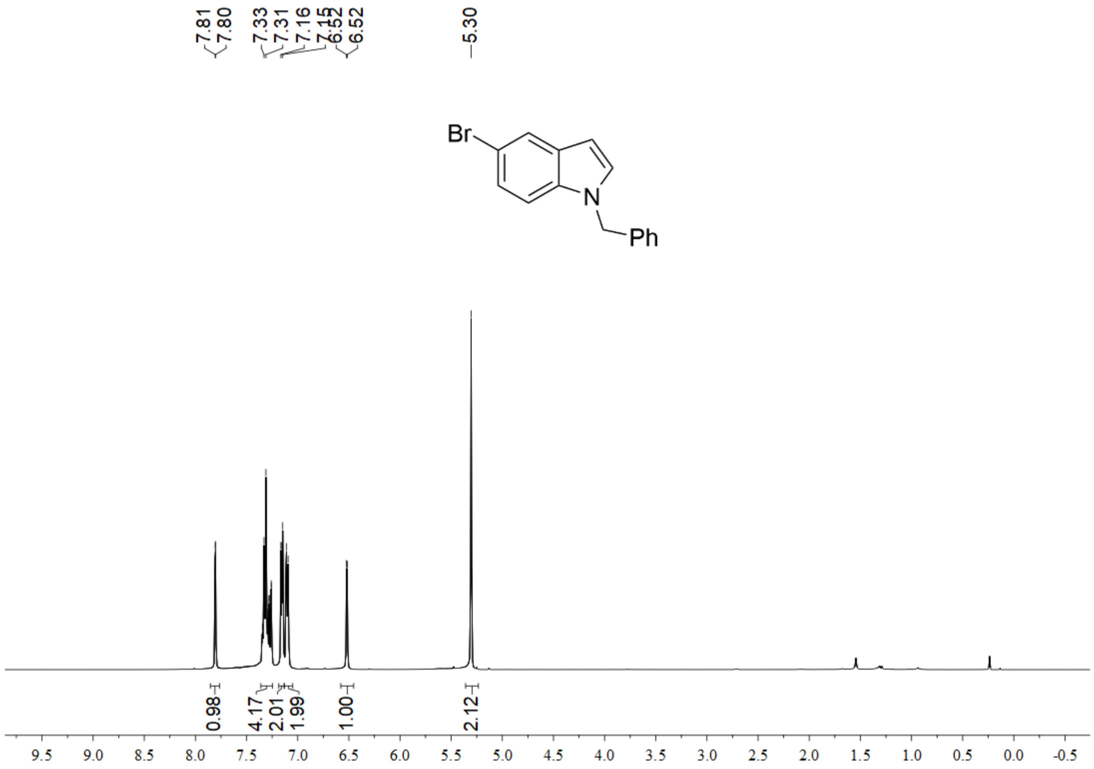

Figure S63 - ^{1}H NMR (400 MHz, $CDCl_3$) of **10b**

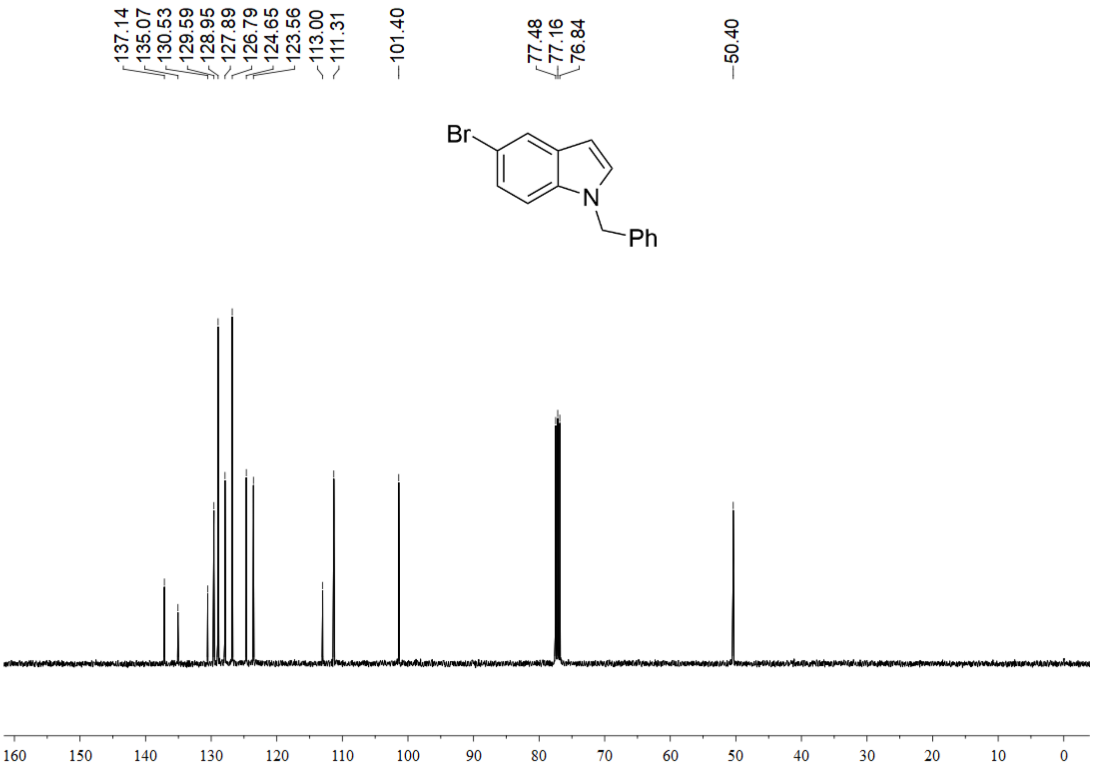

Figure S64 - $^{13}C\{^1H\}$ NMR (101 MHz, $CDCl_3$) of **10b**

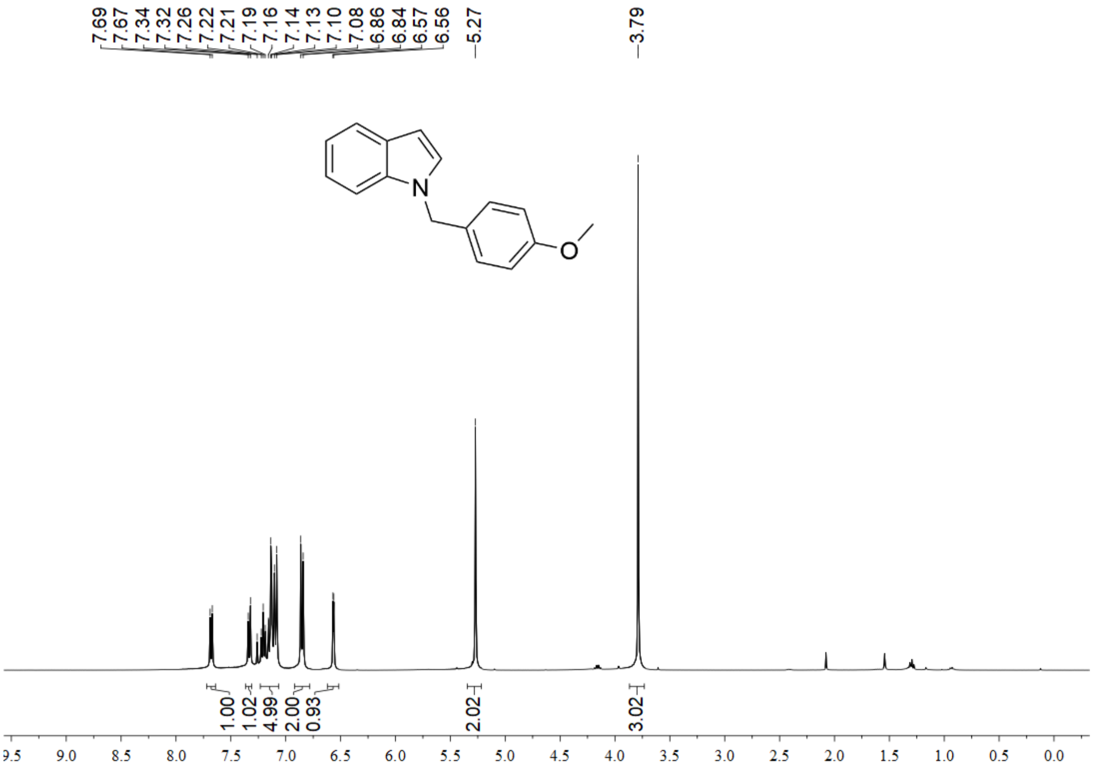

Figure S65 - ^1H NMR (400 MHz, $CDCl_3$) of **10e**

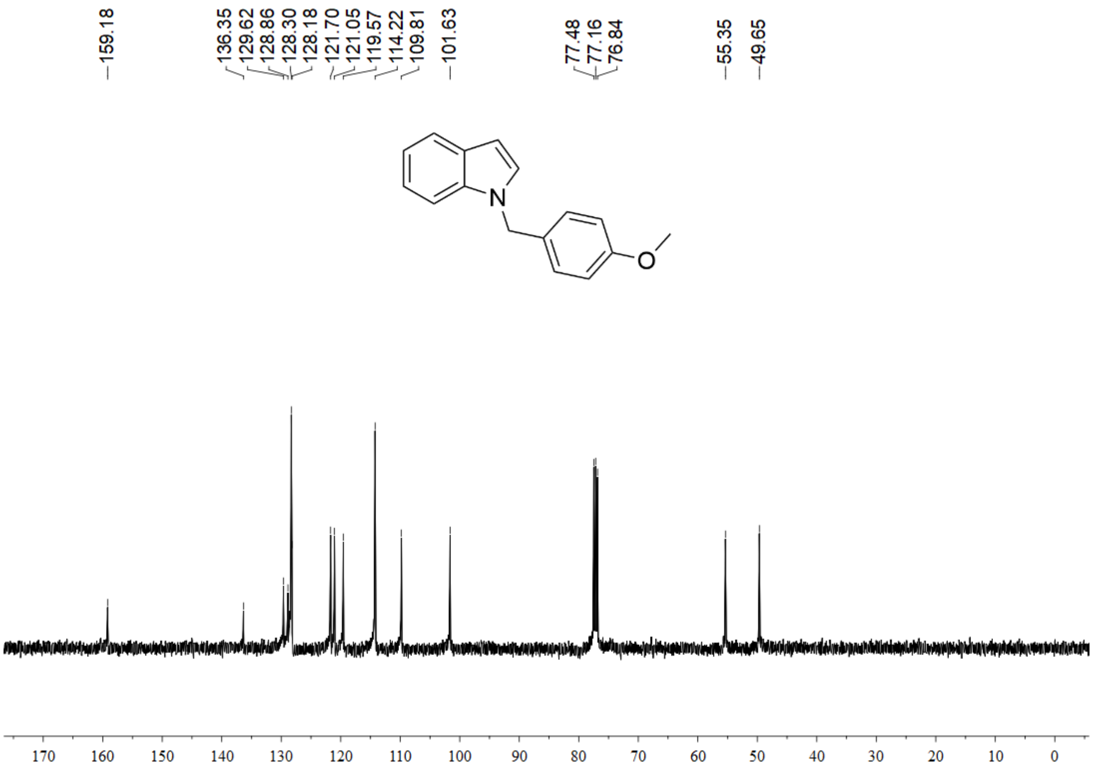

Figure S66 - $^{13}C\{^{1}H\}$ NMR (101 MHz, $CDCl_3$) of **10e**

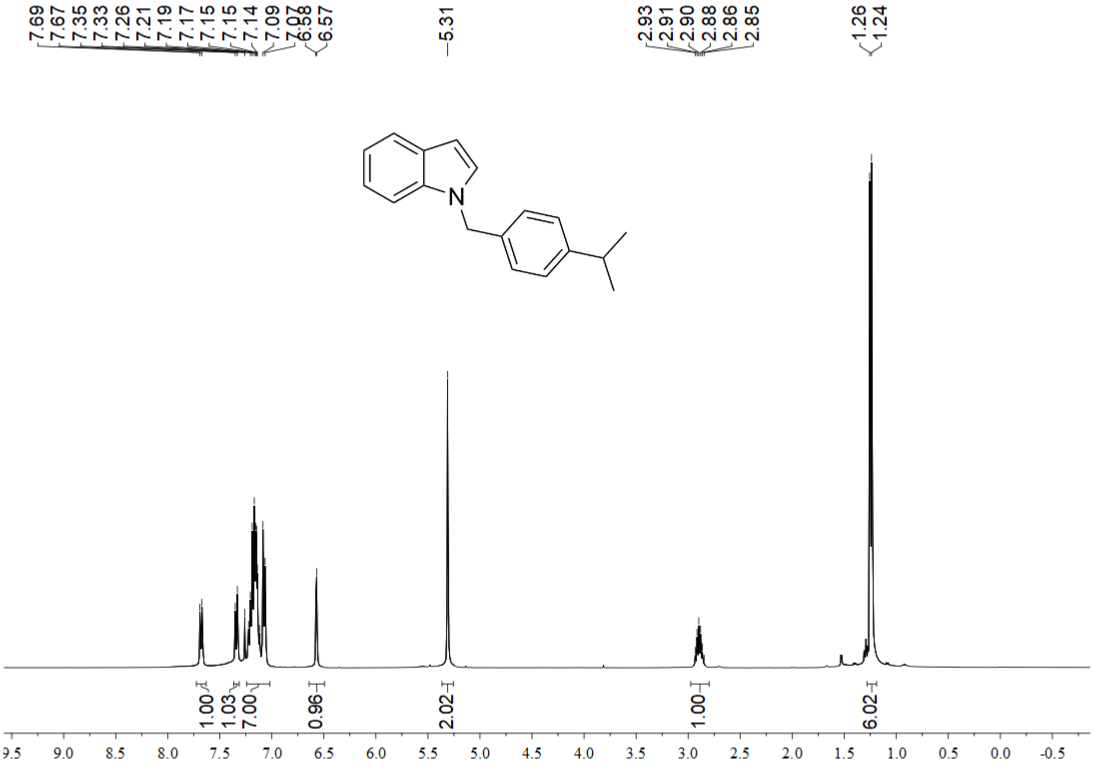

Figure S67 - ^{1}H NMR (400 MHz, $CDCl_3$) of **10f**

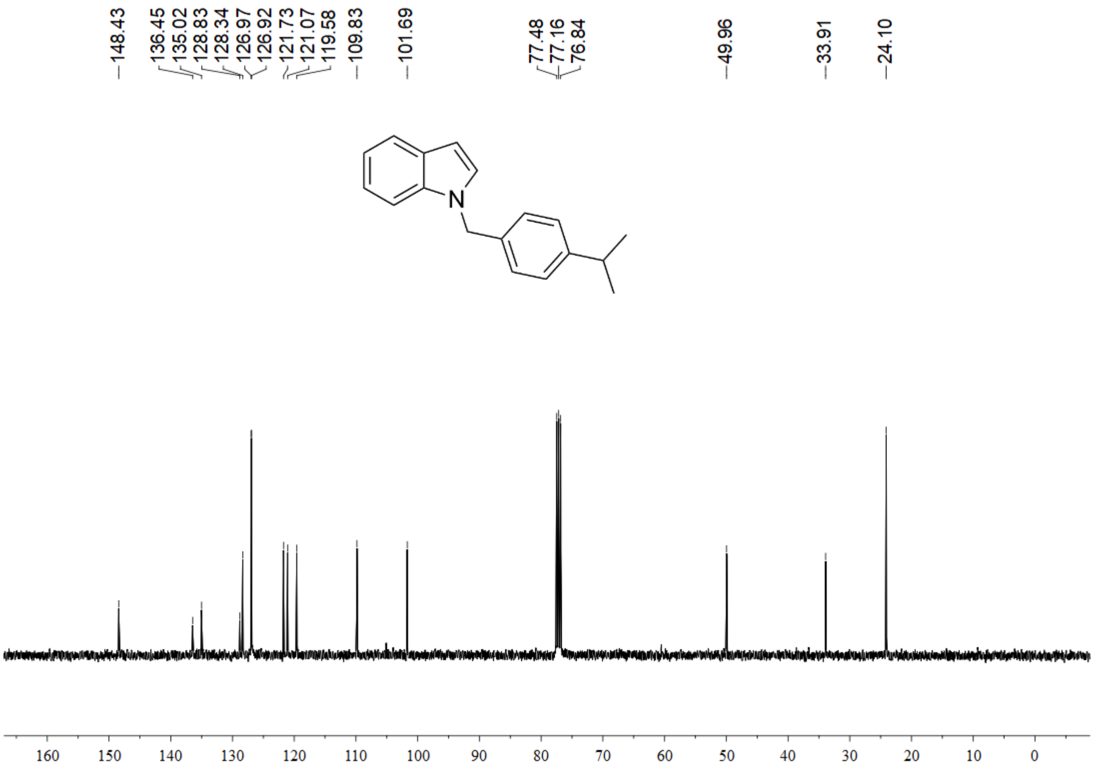

Figure S68 - $^{13}C\{^{1}H\}$ NMR (101 MHz, $CDCl_3$) of **10f**

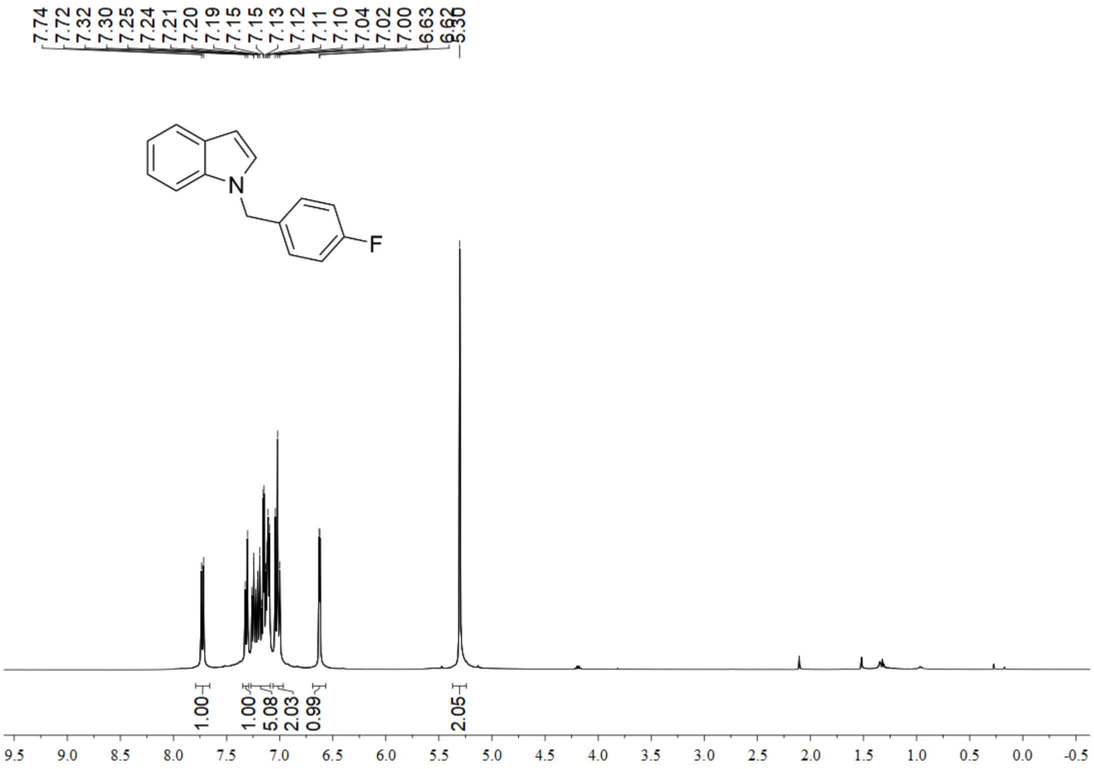

Figure S69 - ^{1}H NMR (400 MHz, $CDCl_3$) of **10g**

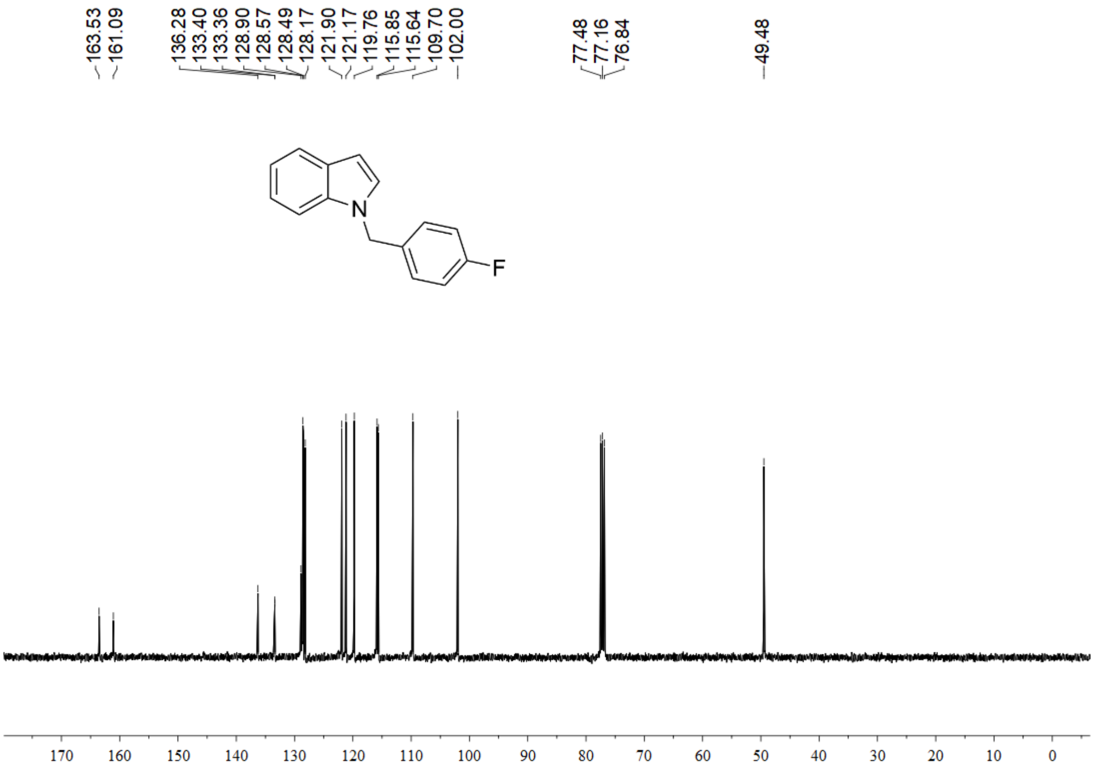

Figure S70 - $^{13}C\{^{1}H\}$ NMR (101 MHz, $CDCl_3$) of **10g**

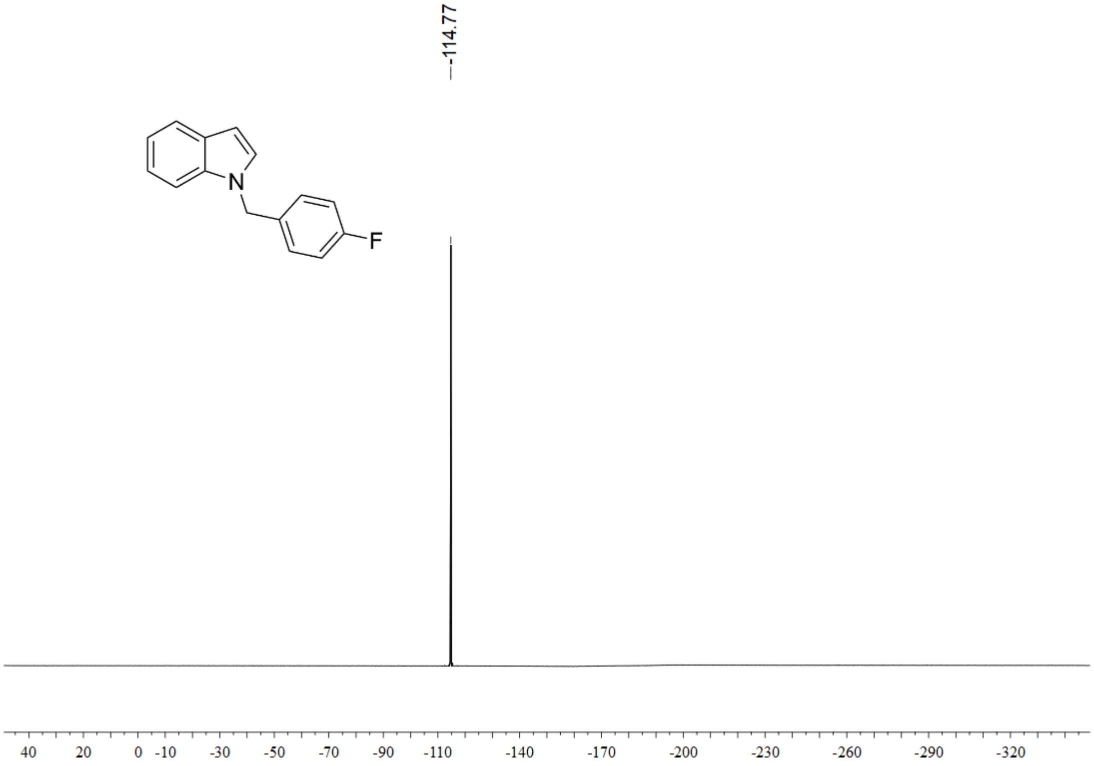

Figure S71 - ^{19}F NMR (376 MHz, $CDCl_3$) of **10g**

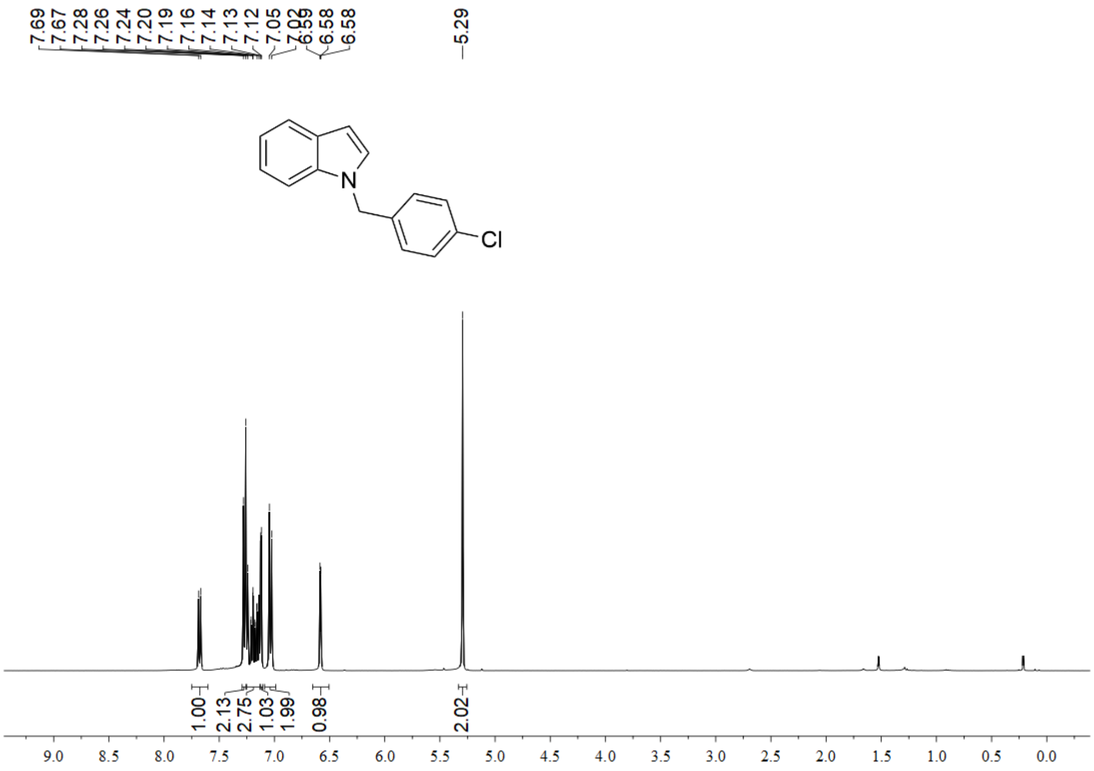

Figure S72 - ^{1}H NMR (400 MHz, $CDCl_3$) of **10h**

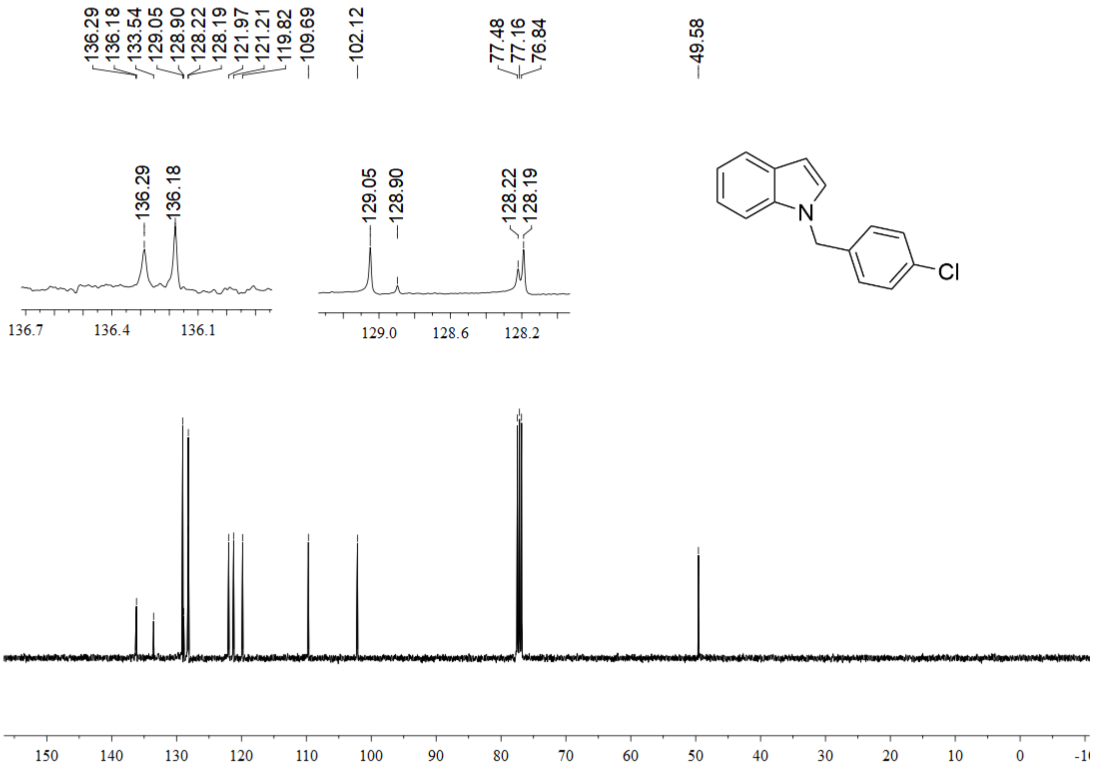

Figure S73 - $^{13}C\{^1H\}$ NMR (101 MHz, $CDCl_3$) of **10h**

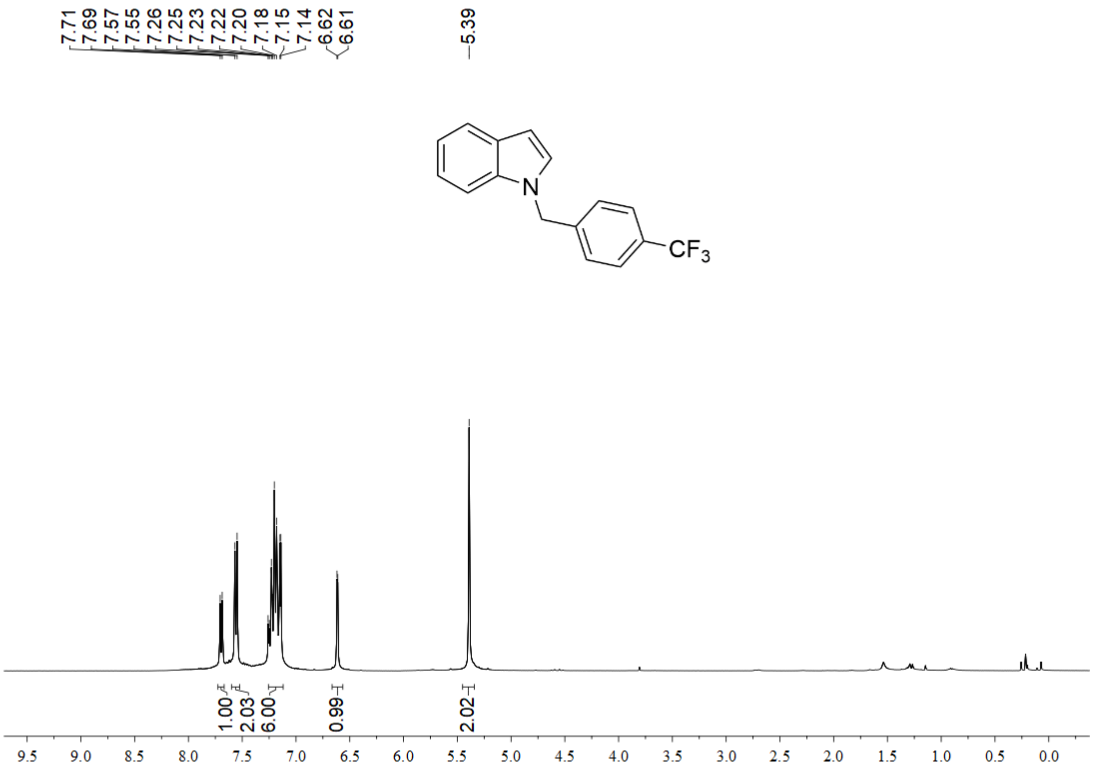

Figure S74 - ^{1}H NMR (400 MHz, $CDCl_3$) of **10j**

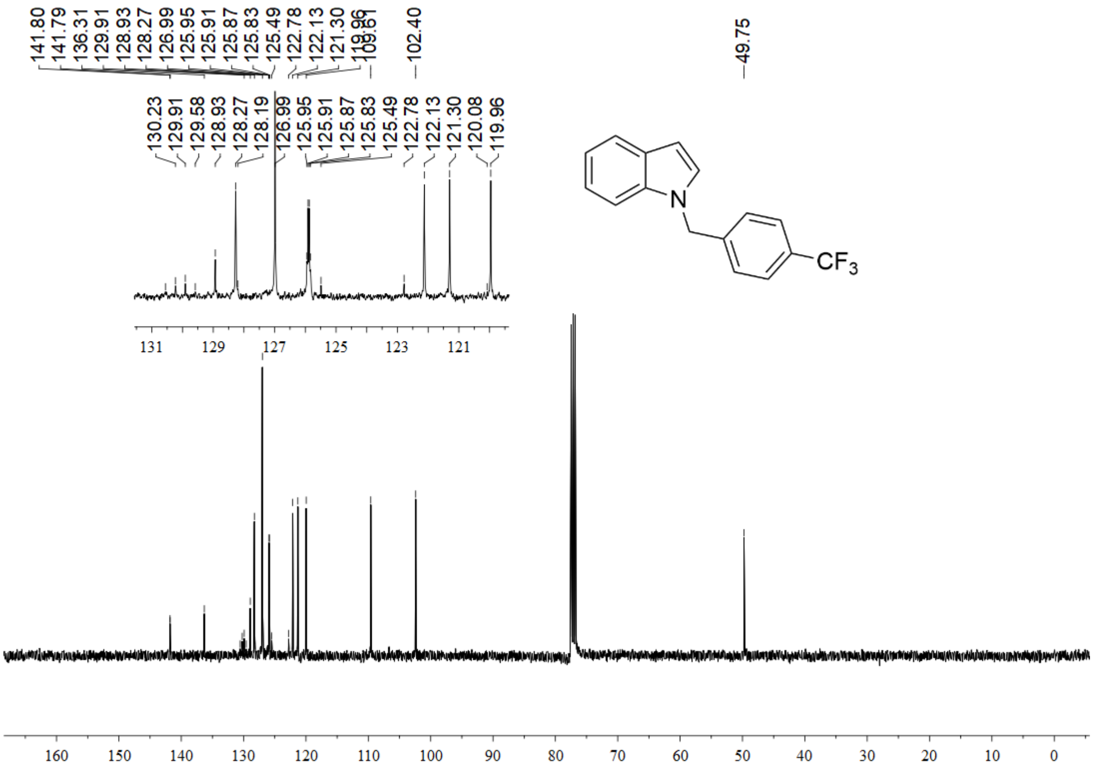

Figure S75 - $^{13}C\{^{1}H\}$ NMR (101 MHz, $CDCl_3$) of **10j**

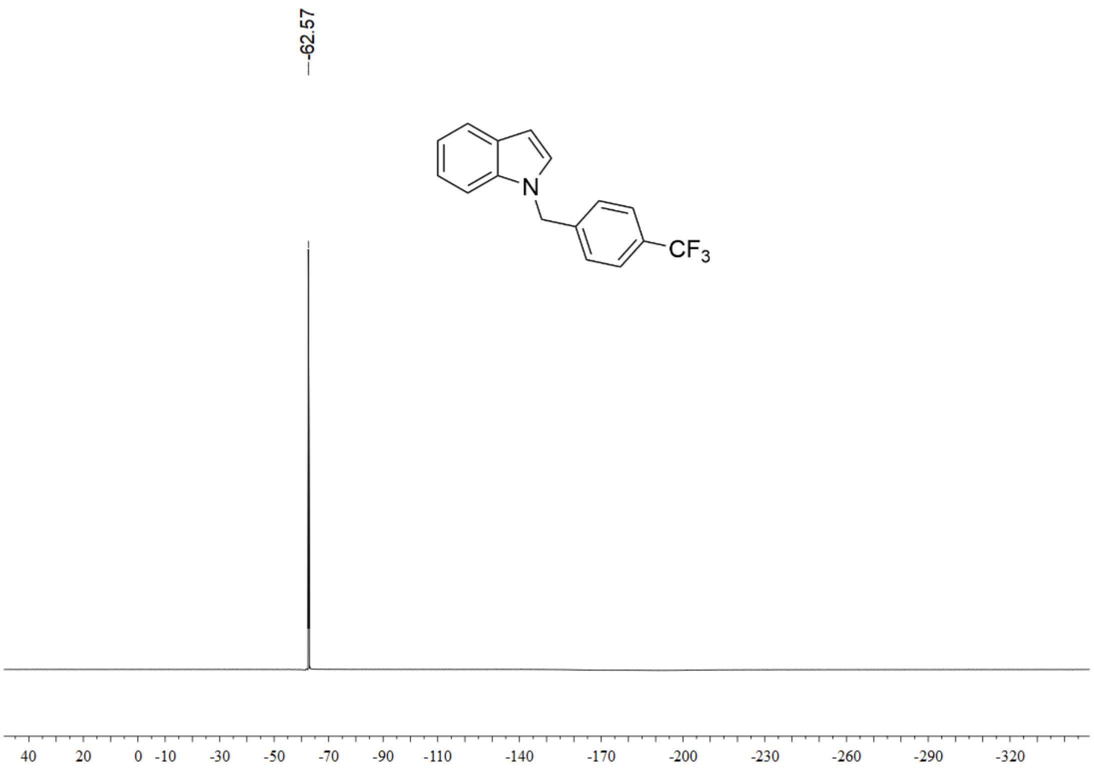

Figure S76 - ^{19}F NMR (376 MHz, $CDCl_3$) of **10j**

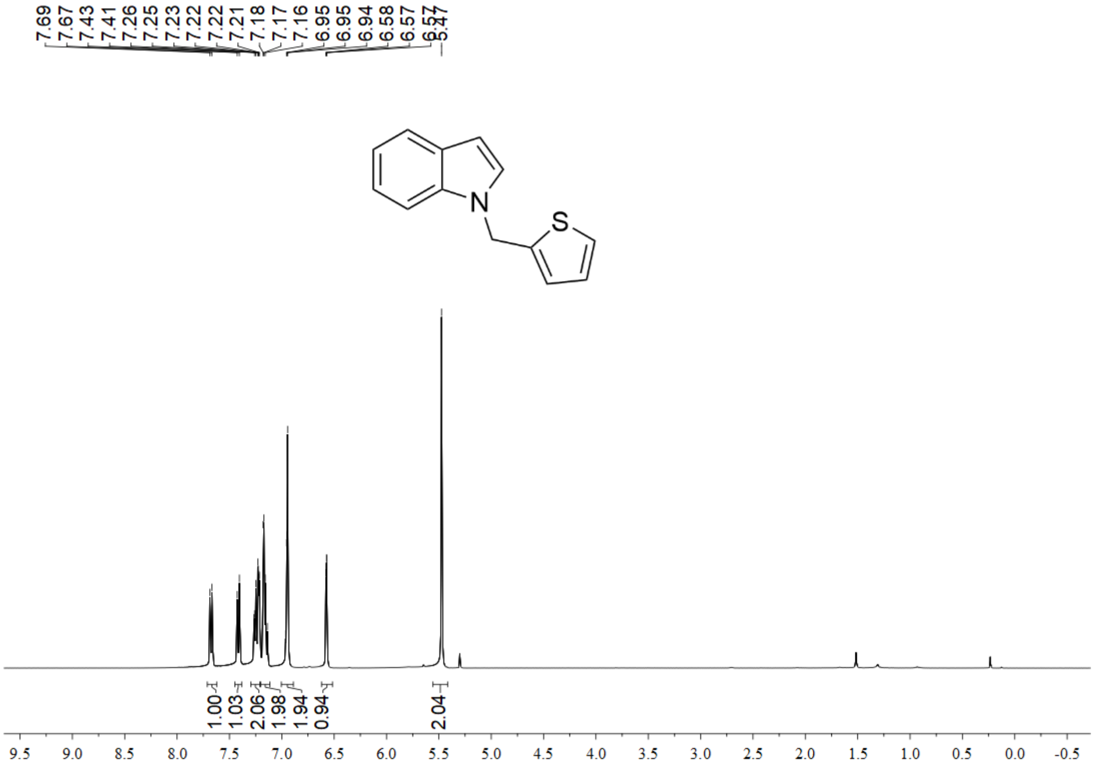

Figure S77 - ^{1}H NMR (400 MHz, $CDCl_3$) of **10l**

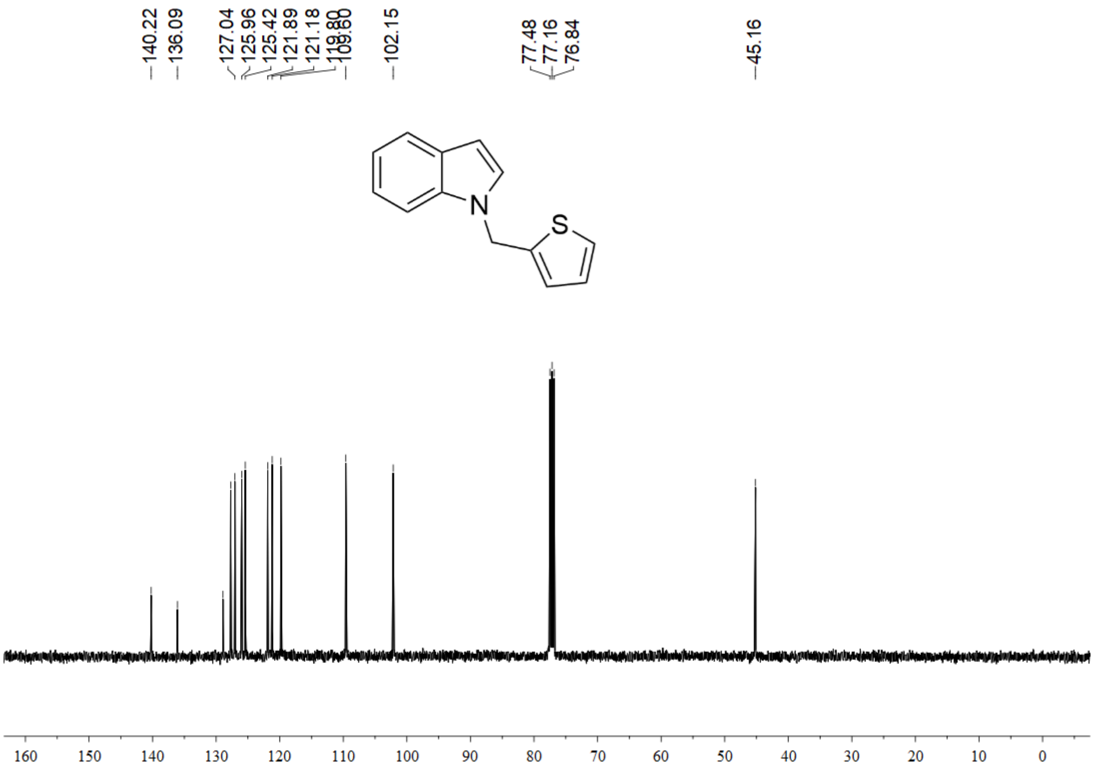

Figure S78 - $^{13}C\{^{1}H\}$ NMR (101 MHz, $CDCl_3$) of **10l**

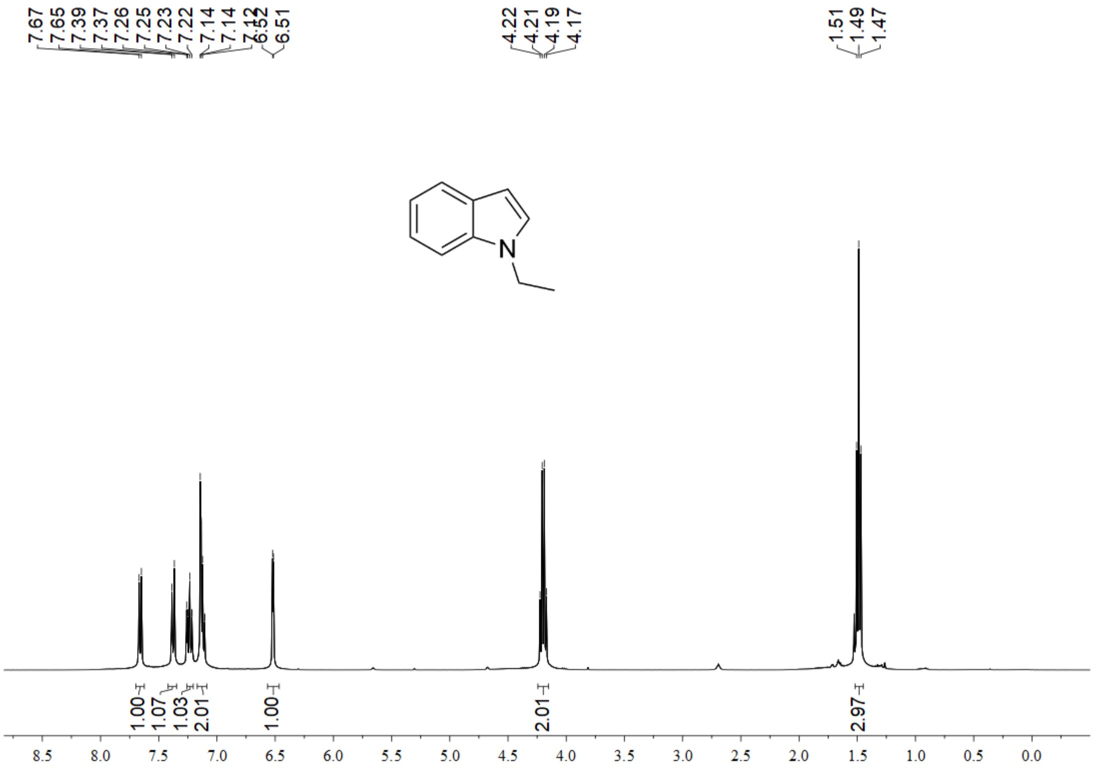

Figure S79 - ^{1}H NMR (400 MHz, $CDCl_3$) of **10n**

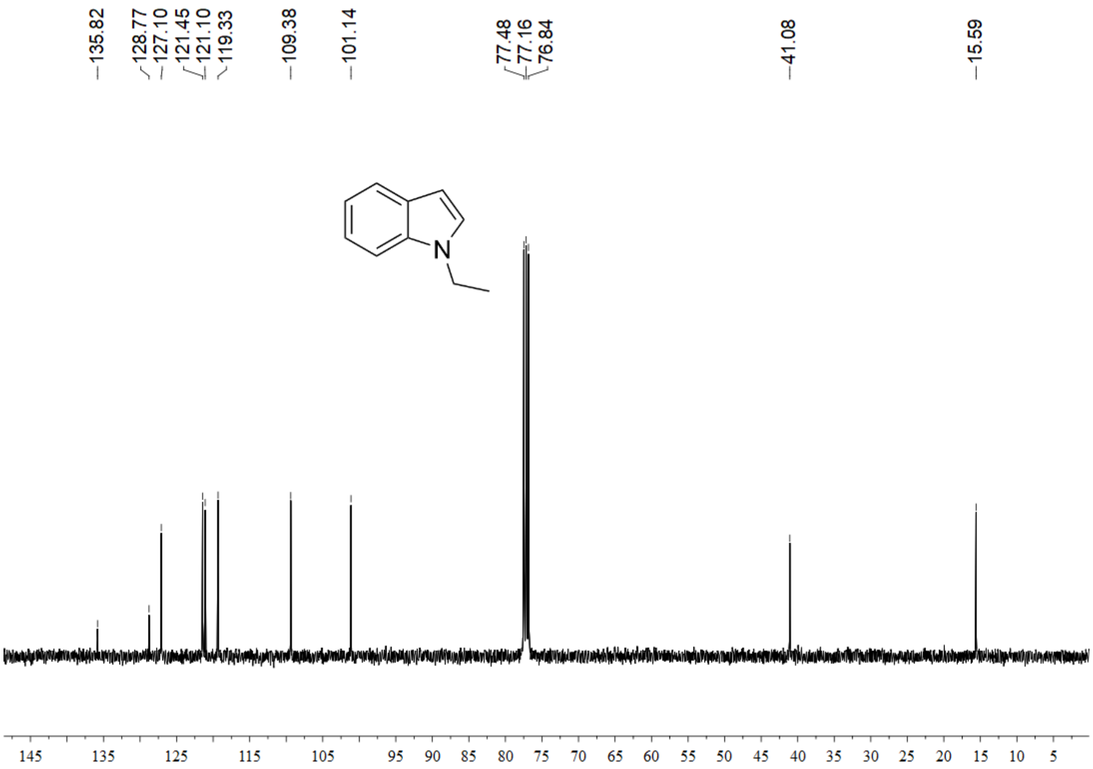

Figure S80 - $^{13}C\{^{1}H\}$ NMR (101 MHz, $CDCl_3$) of **10n**

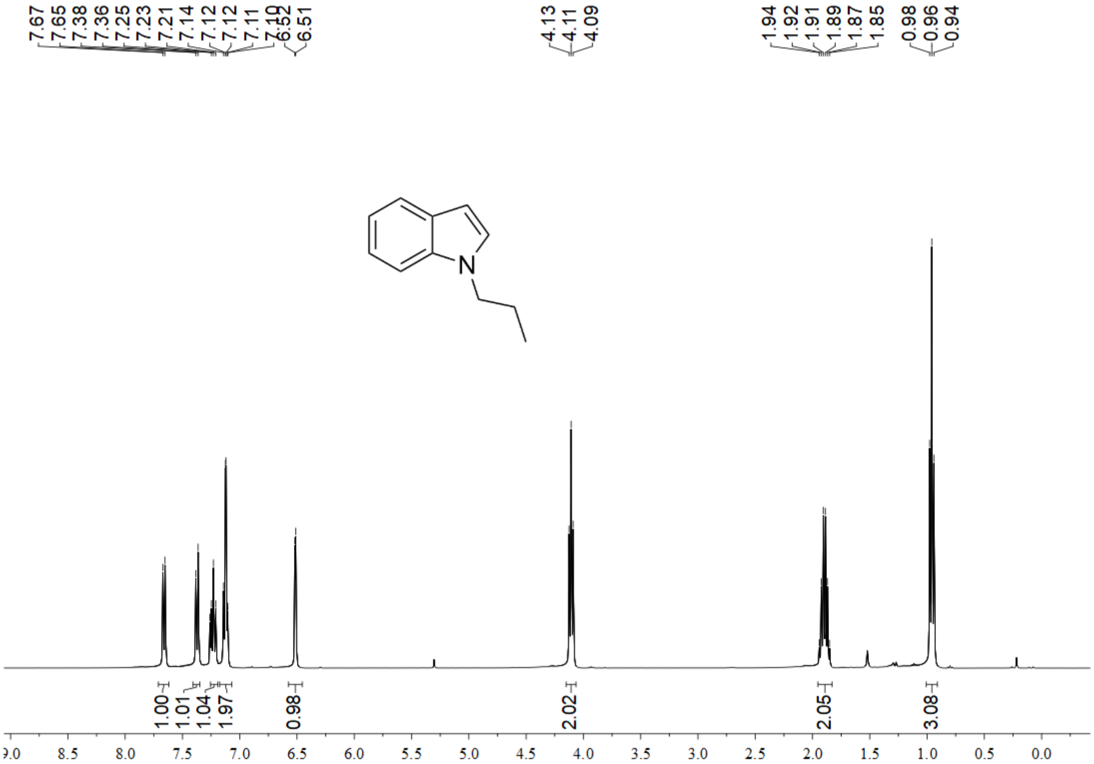

Figure S81 - ^{1}H NMR (400 MHz, $CDCl_3$) of **10o**

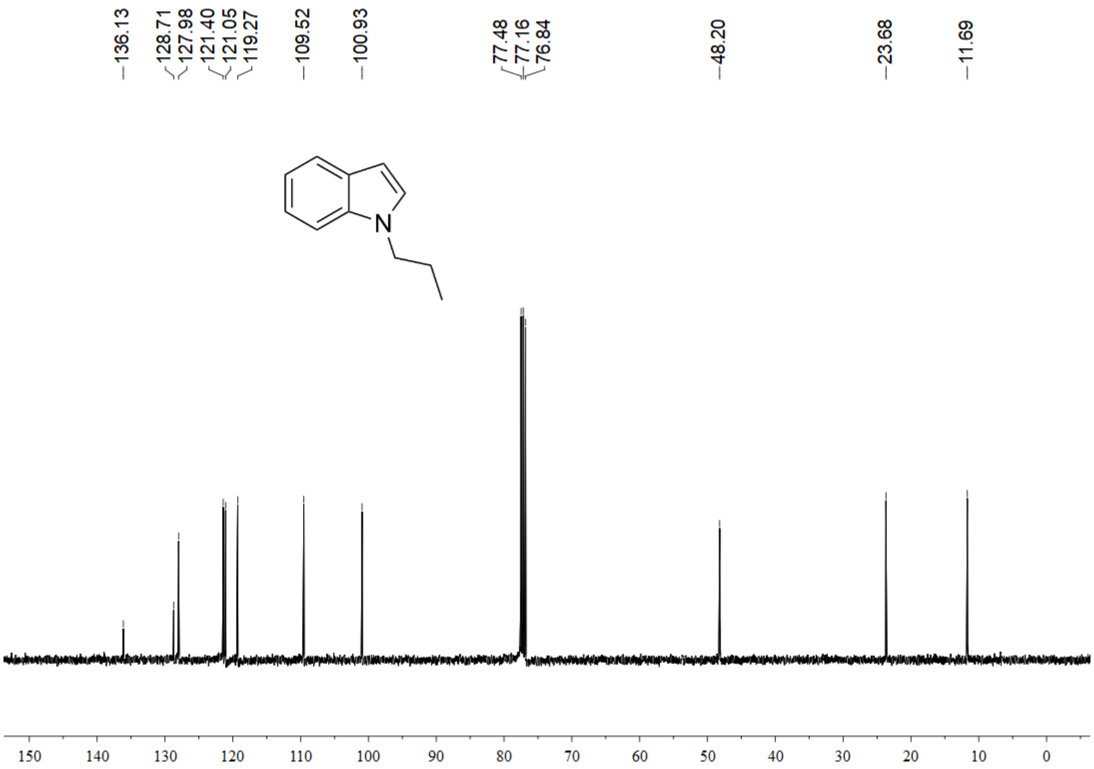

Figure S82 - $^{13}C\{^{1}H\}$ NMR (101 MHz, $CDCl_3$) of **10o**

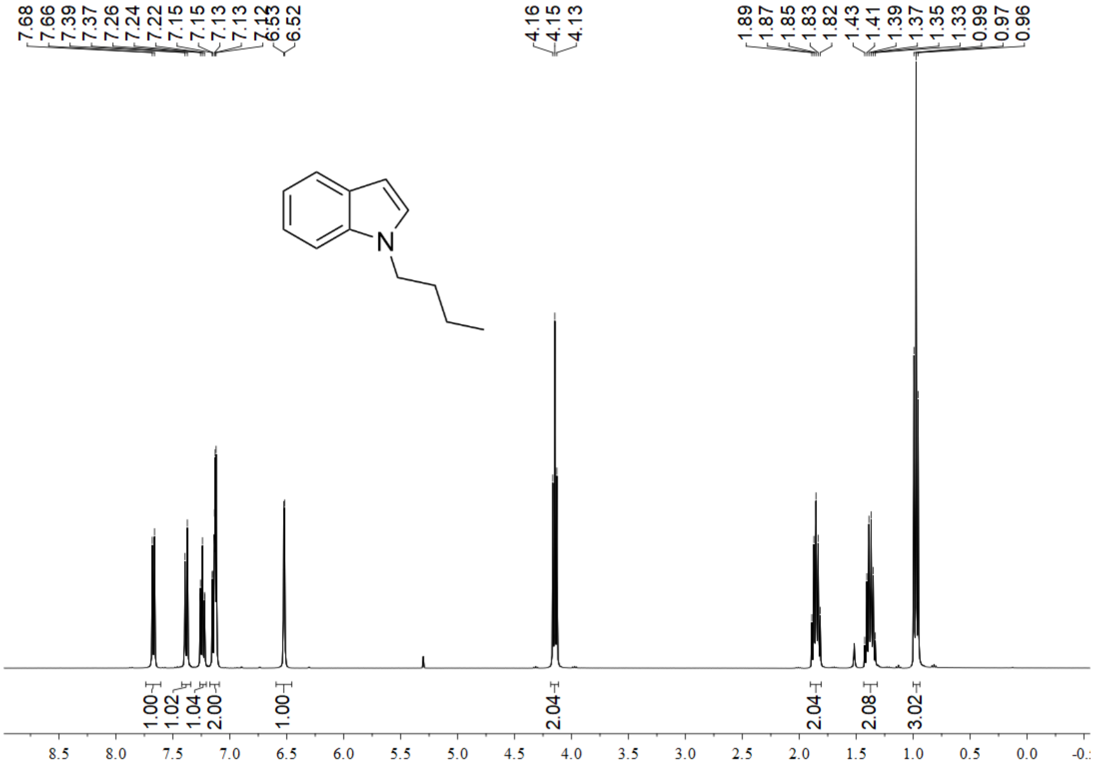

Figure S83 - ^{1}H NMR (400 MHz, $CDCl_3$) of **10p**

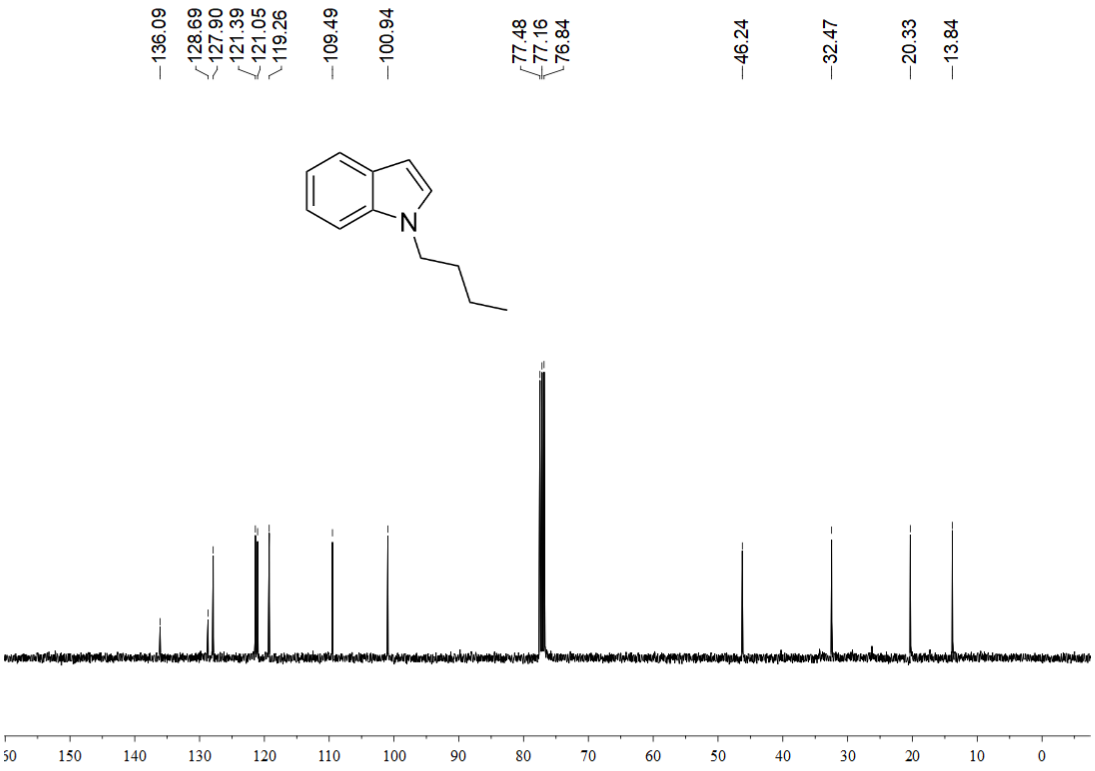

Figure S84 - $^{13}C\{^{1}H\}$ NMR (101 MHz, $CDCl_3$) of **10p**

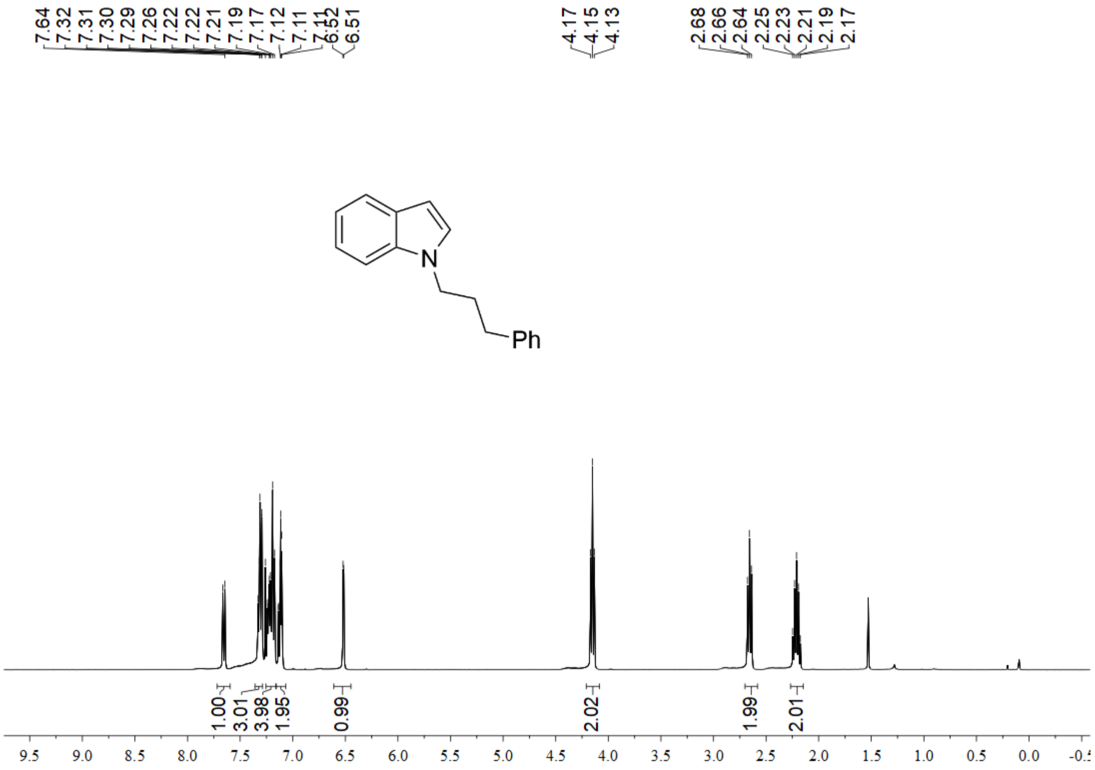

Figure S85 - ^{1}H NMR (400 MHz, $CDCl_3$) of **10q**

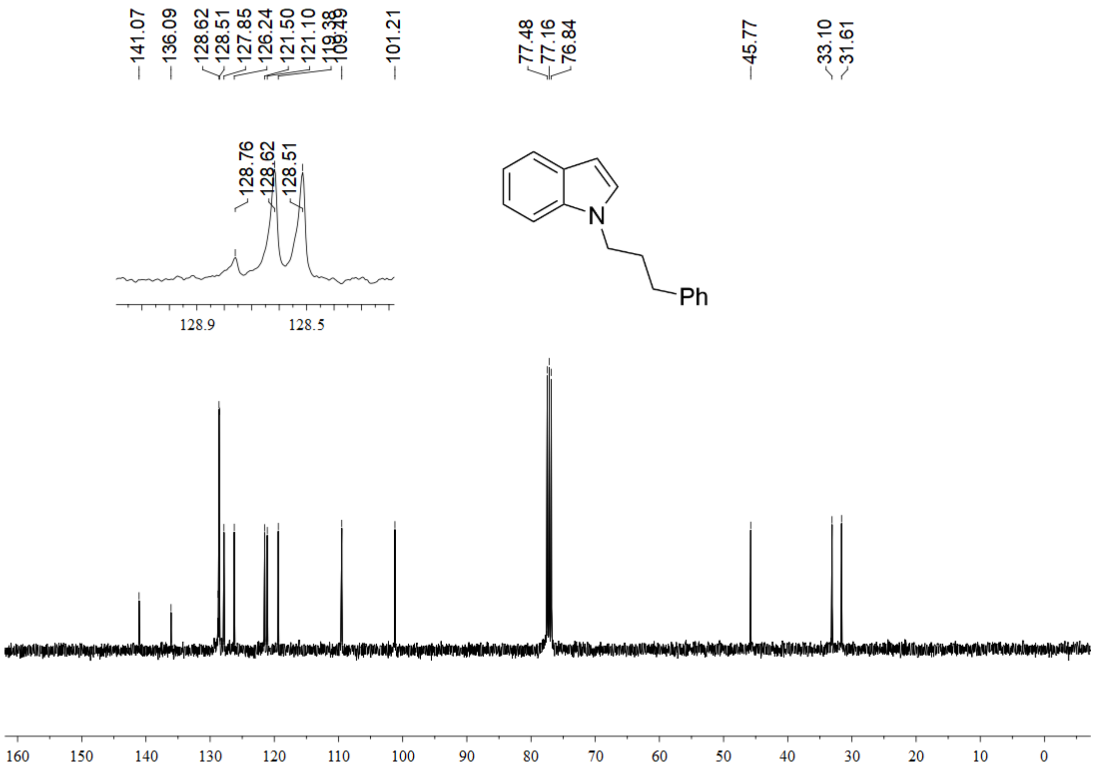

Figure S86 - $^{13}C\{^{1}H\}$ NMR (101 MHz, $CDCl_3$) of **10q**

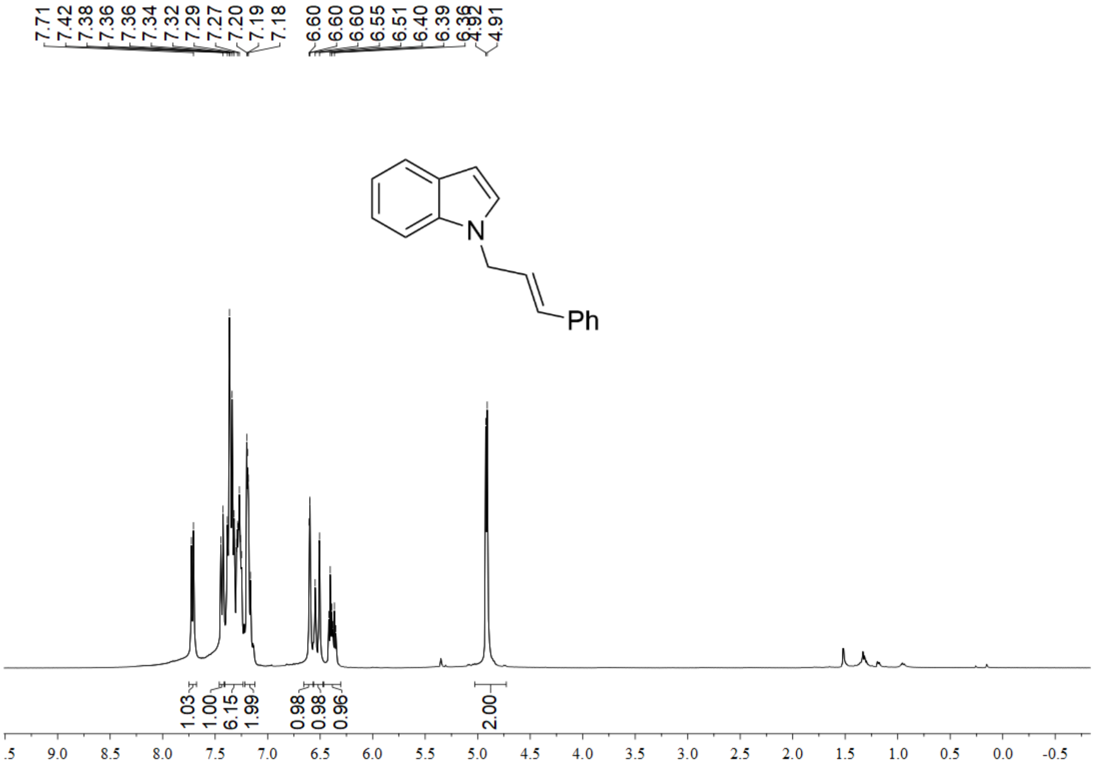

Figure S87 - ^{1}H NMR (400 MHz, $CDCl_3$) of **10r**

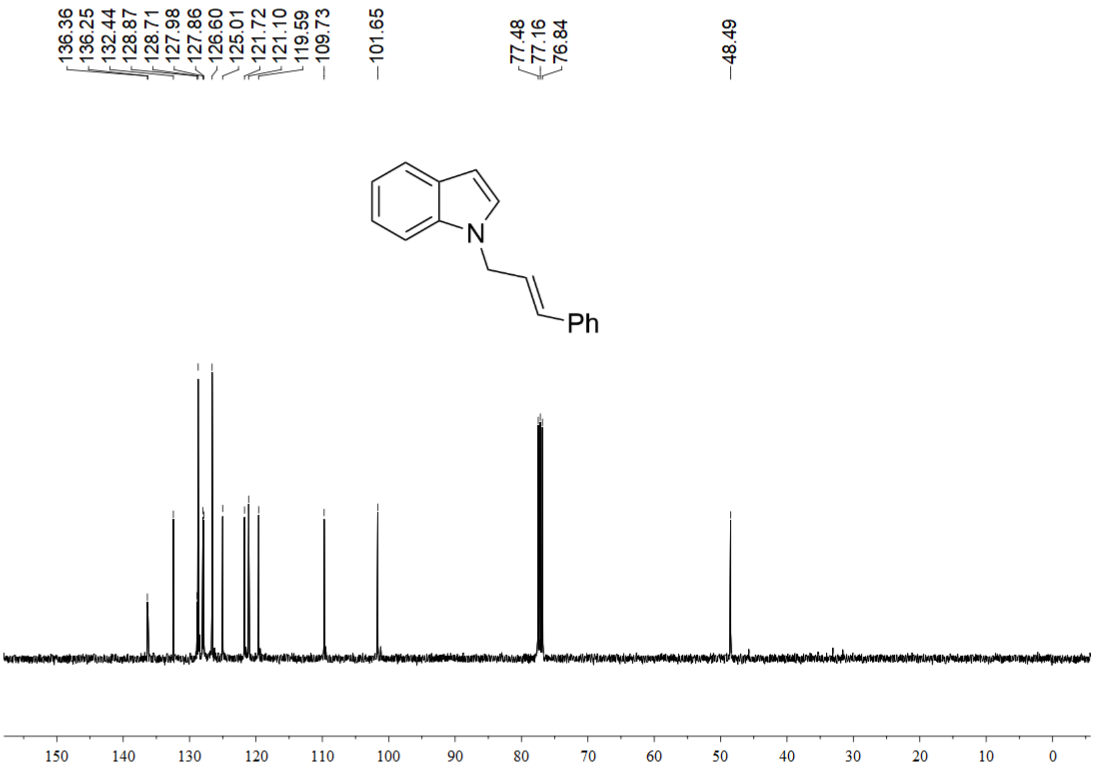

Figure S88 - $^{13}C\{^{1}H\}$ NMR (101 MHz, $CDCl_3$) of **10r**

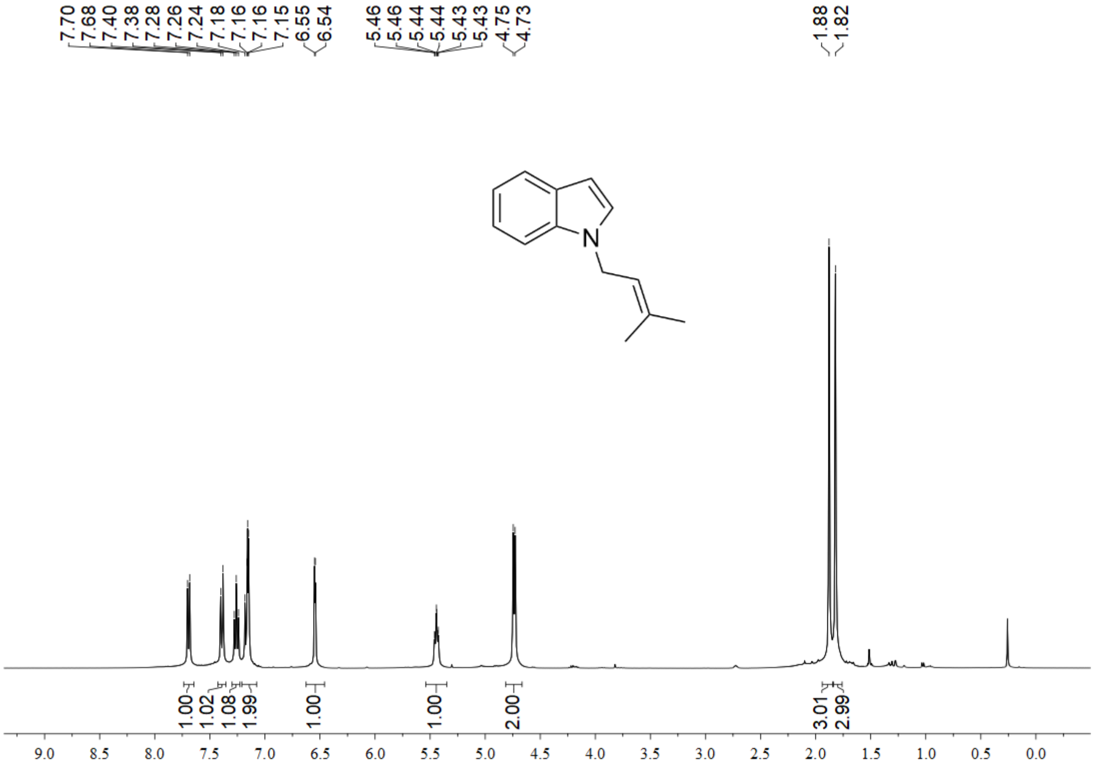

Figure S89 - ^{1}H NMR (400 MHz, $CDCl_3$) of **10s**

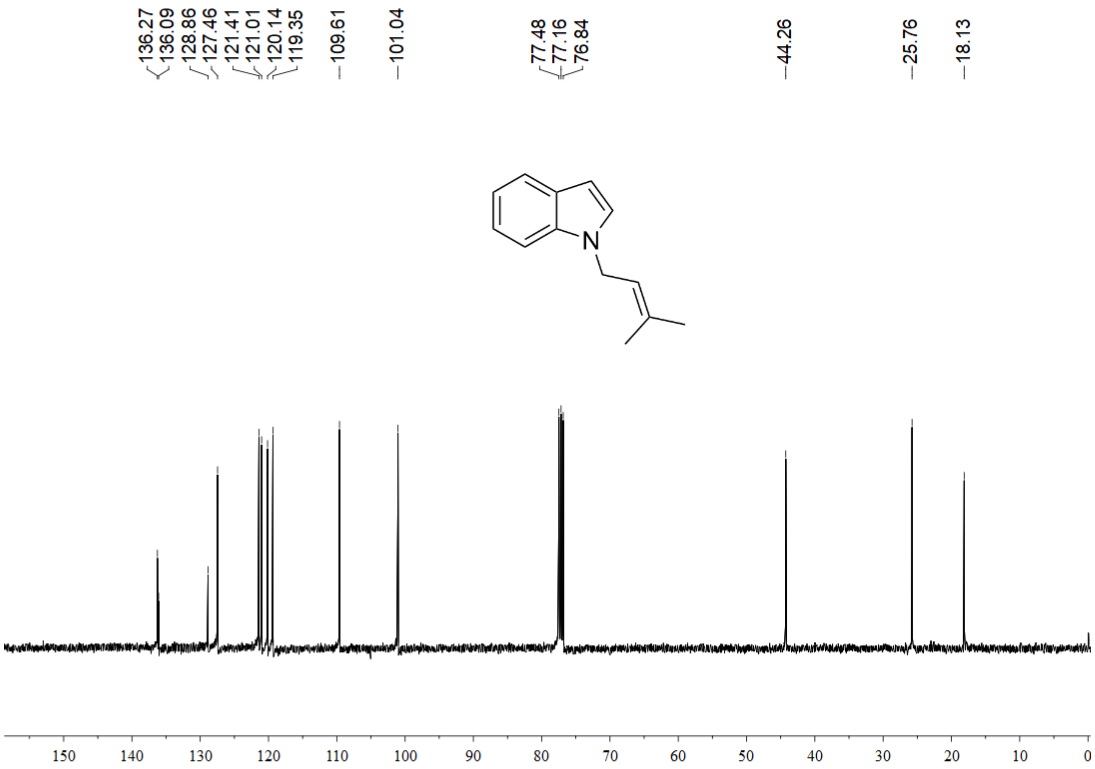

Figure S90 - $^{13}C\{^{1}H\}$ NMR (101 MHz, $CDCl_3$) of **10s**

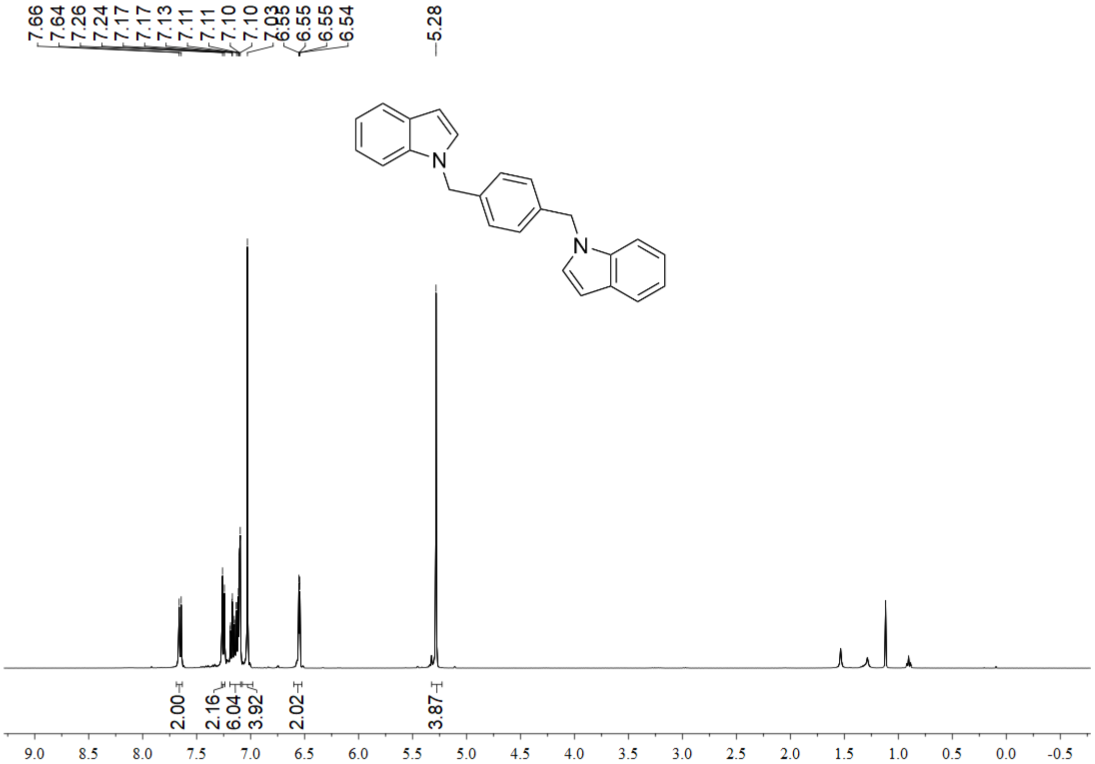

Figure S91 - ^{1}H NMR (400 MHz, $CDCl_3$) of **10aa**

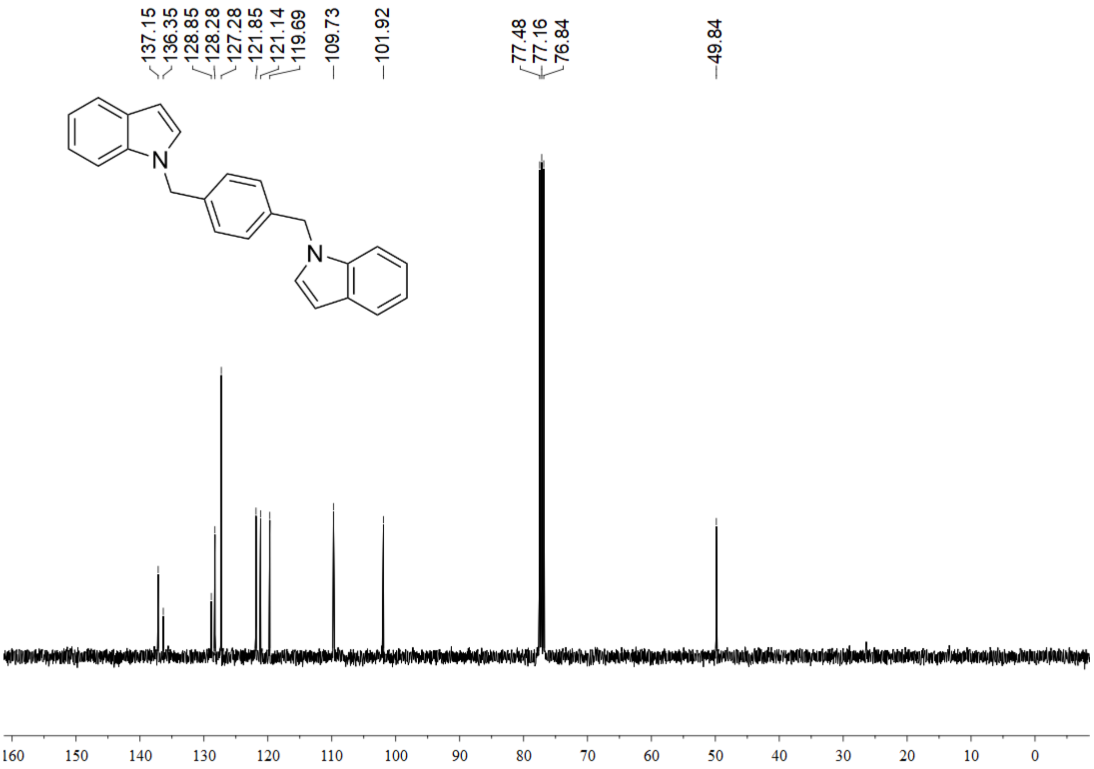

Figure S92 - $^{13}C\{^1H\}$ NMR (101 MHz, $CDCl_3$) of **10aa**

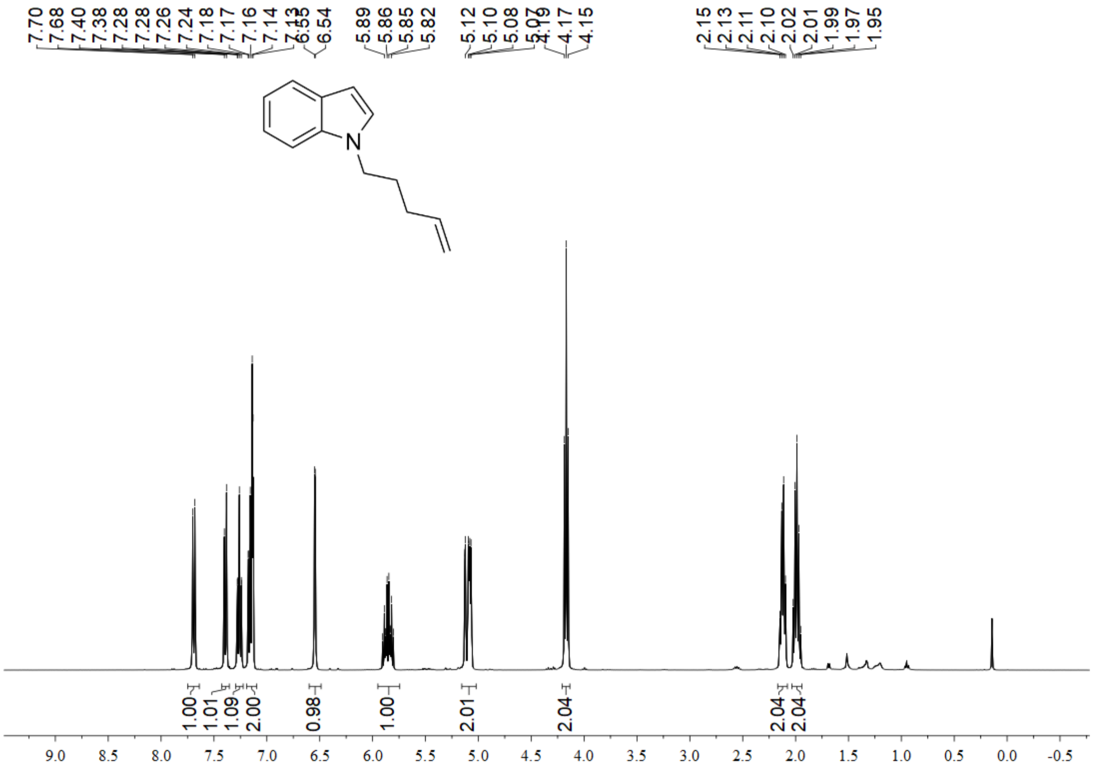

Figure S93 - ^1H NMR (400 MHz, $CDCl_3$) of **10-1**

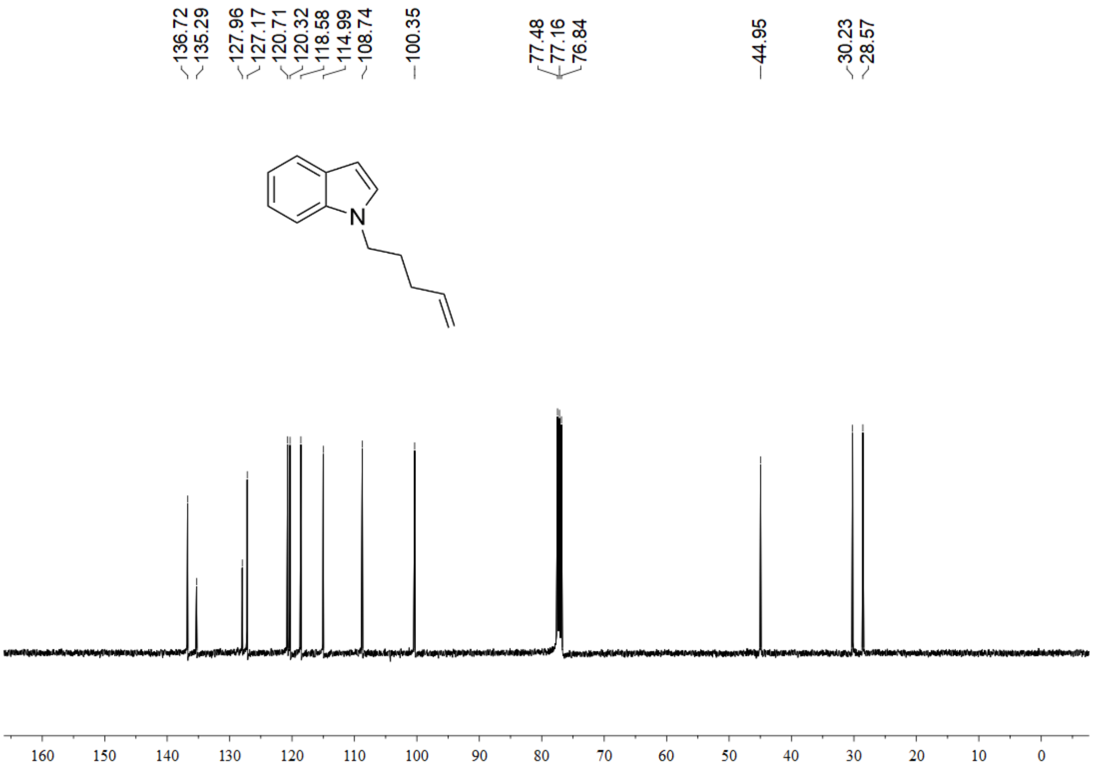

Figure S94 - $^{13}C\{^{1}H\}$ NMR (101 MHz, $CDCl_3$) of **10-1**

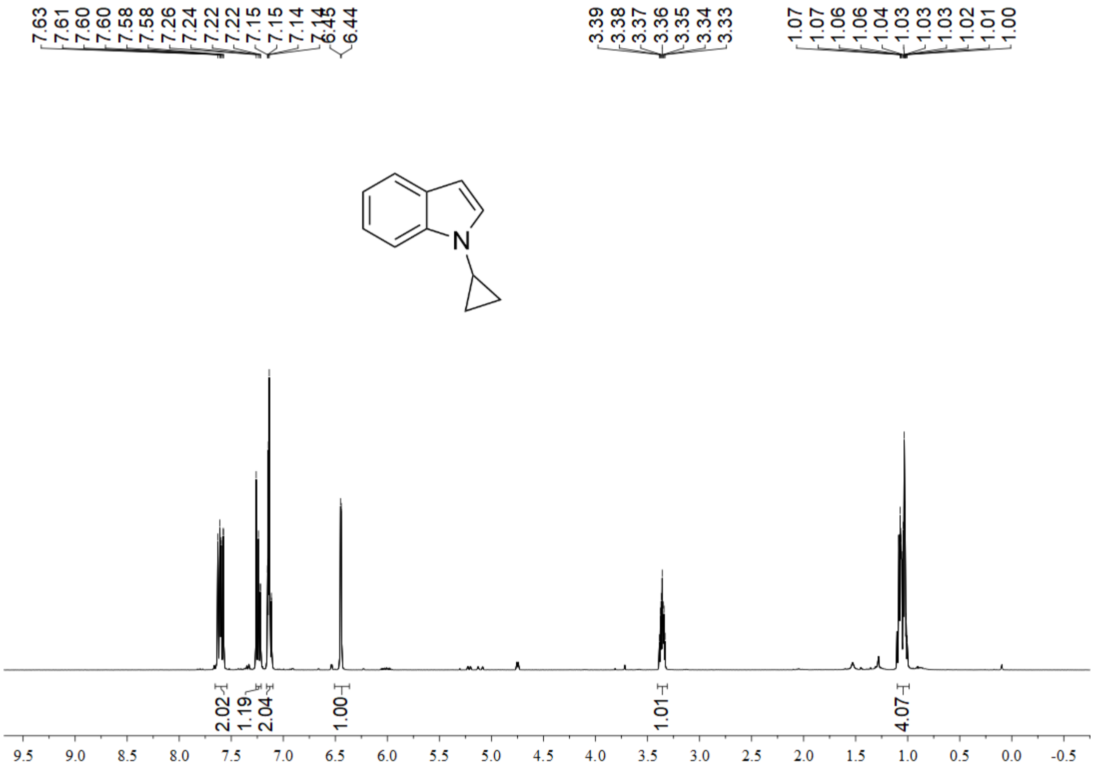

Figure S95 - ^{1}H NMR (400 MHz, $CDCl_3$) of **10-2**

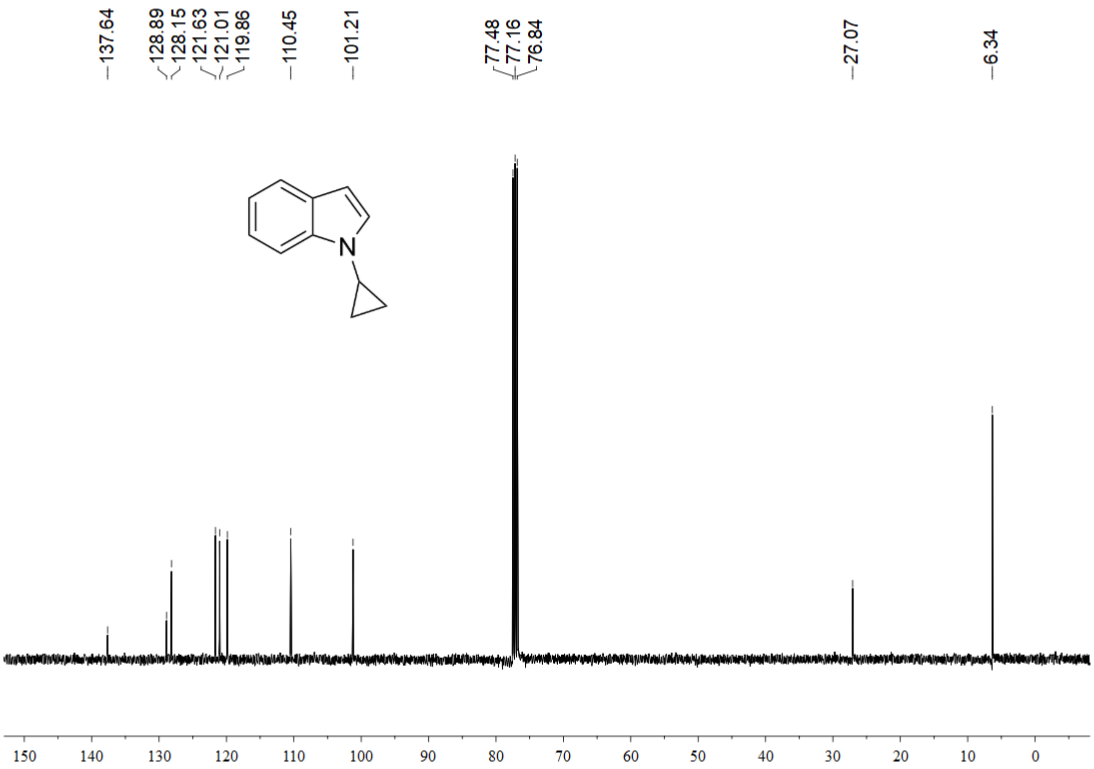

Figure S96 - $^{13}C\{^1H\}$ NMR (101 MHz, $CDCl_3$) of **10-2**

9) Reference

[1] R. Omar-Amrani, A. Thomas, E. Brenner, R. Schneider, Y. Fort, *Org. Lett.* **2003**, *5*, 2311.

[2] A. F. G. Maier, S. Tussing, T. Schneider, U. Flörke, Z-W. Qu, S. Grimme, J. Paradies, *Angew. Chem. Int. Ed.* **2016**, *55*, 12219.

[3] M. J. Tomaszewski, J. Warkentin, N. H. Werstiuk, *Aust. J. Chem*. **1995**, *48*, 291.

[4] A. Kaga, H. Hayashi, H. Hakamata, M. Oi, M. Uchiyama, R. Takita, S. Chiba, *Angew. Chem. Int. Ed.* **2017**, *56*, 11807.

[5] C. Lu, Z. Qiu, M. Xuan, Y. Huang, Y. Lou, Y. Zhu, H. Shen, B-L. Lin, *Adv. Synth. Catal.* **2020**, *362*, 4151.

[6] K. Okamoto, S. Nagahara, Y. Imada, R. Narita, Y. Kitano, K. Chiba, *J. Org. Chem.* **2021**, *86*, 15992.

[7] Y. Wei, Q. Xuan, Y. Zhou, Q. Song, *Org. Chem. Front*. **2018**, *5*, 3510.

[8] J. C. Borghs, V. Zubar, L. M. Azofra, J. Sklyaruk, M. Rueping, *Org. Lett*. **2020**, *22*, 4222.

[9] G. Hirata, H. Satomura, H. Kumagae, A. Shimizu, G. Onodera, M. Kimura, *Org. Lett*. **2017**, *19*, 6148.

[10] X. Zeng, M. Soleilhavoup, G. Bertrand, *Org. Lett*. **2009**, *11*, 3166.

[11] B. Ertugrul, H. Kilic, F. Lafzi, N. Saracoglu, *J. Org. Chem.* **2018**, *83*, 9018.

[12] T. Torigoe, T. Ohmura, M. Suginome, *Angew. Chem. Int. Ed.* **2017**, *56*, 14272.

[13] S. Tamura, A. Sugawara, E. Sato, F. Sato, K. Sato, T. Kawano, *Tetrahedron Lett.* **2020**, *61*, 151919.

[14] G. A. Molander, D. Ryu, M. Hosseini-Sarvari, R. Devulapally, D. Seapy, *J. Org. Chem.* **2013**, *78*, 6648.

[15] X. Jiang, W. Tang, D. Xue, J. Xiao, C. Wang, *ACS Catal*. **2017**, *7*, 1831.

[16] C-Y. Chang, Y-H. Lin, Y-K. Wu, *Chem. Commun*. **2019**, *55*, 1116.

[17] E. A Petrushkina, S. A. Chae, S. C. Shim, *Inorg. Chem. Commun*. **1998**, *1*, 284.

[18] J. Bloxham, C. J. Moody, A. M. Z. Slawin, *Tetrahedron* **2002**, *58*, 3709.

[19] A. P. Dobbs, K. Jones, K. T. Veal, *Tetrahedron* **1998**, *54*, 2149.

[20] T. Tsuritani, N. A. Strotman, Y. Yamamoto, M. Kawasaki, N. Yasuda, T. Mase, *Org. Lett.* **2008**, *10*, 1653.
